# Investigating the ID3/SLC22A4 as immune-related signatures in ischemic stroke

**DOI:** 10.18632/aging.205308

**Published:** 2023-12-14

**Authors:** Dading Lu, Heng Cai, Yugang Li, Wenyuan Chang, Xiu Liu, Qiwei Dai, Wanning Yu, Wangli Chen, Guomin Qiao, Haojie Xie, Xiong Xiao, Zhiqing Li

**Affiliations:** 1 Department of Stroke Center, The First Hospital of China Medical University, Heping, Shenyang, Liaoning, China; 2 Department of Neurology, The First Hospital of China Medical University, Heping, Shenyang, Liaoning, China; 3 Department of Neurosurgery, The First Hospital of China Medical University, Heping, Shenyang, Liaoning, China; 4 Department of Neurosurgery, Shengjing Hospital, Shenyang, China medical University, Heping, Shenyang, China; 5 The First Clinical College, China Medical University, Shenbei, Shenyang, China

**Keywords:** ischemic stroke, ID3, SLC22A4, bioinformation, immune

## Abstract

Background: Ischemic stroke (IS) is a fearful disease that can cause a variety of immune events. Nevertheless, precise immune-related mechanisms have yet to be systematically elucidated. This study aimed to identify immune-related signatures using machine learning and to validate them with animal experiments and single cell analysis.

Methods: In this study, we screened 24 differentially expressed genes (DEGs) while identifying immune-related signatures that may play a key role in IS development through a comprehensive strategy between least absolute shrinkage and selection operation (LASSO) regression, support vector machine (SVM) and immune-related genes. In addition, we explored immune infiltration using the CIBERSORT algorithm. Finally, we performed validation in mouse brain tissue and single cell analysis.

Results: We identified 24 DEGs for follow-up analysis. ID3 and SLC22A4 were finally identified as the better immune-related signatures through a comprehensive strategy among DEGs, LASSO, SVM and immune-related genes. RT-qPCR, western blot, and immunofluorescence revealed a significant decrease in ID3 and a significant increase in SLC22A4 in the middle cerebral artery occlusion group. Single cell analysis revealed that ID3 was mainly concentrated in endothelial_2 cells and SLC22A4 in astrocytes in the MCAO group. A CIBERSORT finds significantly altered levels of immune infiltration in IS patients.

Conclusions: This study focused on immune-related signatures after stroke and ID3 and SLC22A4 may be new therapeutic targets to promote functional recovery after stroke. Furthermore, the association of ID3 and SLC22A4 with immune cells may be a new direction for post-stroke immunotherapy.

## INTRODUCTION

Ischemic stroke accounts for more than 80% of clinical strokes worldwide [1]. It kills millions of people each year, leaving most survivors with permanent disability, placing a huge burden on individuals and society [2]. Therefore, early diagnosis and treatment for IS will be a top priority in modern healthcare. A review of the development of stroke reveals that disruption of the blood-brain barrier may lead to a disturbance of the central immune microenvironment, which may invite more inflammatory cells to infiltrate and cause a more intense immune response, which may further aggravate the condition [3]. Now, immunomodulation has been shown to effectively delay ischemic stroke and promote neurological recovery [4]. This again emphasises the importance of maintaining the balance of the immune microenvironment for the protection of the central nervous system.

Therefore, to better guide therapeutic interventions, such as drug development and repurposing, it is necessary to deepen our understanding about immune mechanisms associated with stroke to demonstrate target genes and pathways better [5]. Analysis of Disease-related gene expression or transcriptomic data can yield valuable insights. In this process, *in silico*, gene expression data can identify heterogeneous cell populations in a sample, including immune activating subpopulations [6]. Furthermore, with the constant iteration of gene microarray technology, machine learning has become increasingly involved in modern biomedicine, specifically, whether it is analysing huge amounts of gene expression profiles or finding relevant biological features, machine learning is well equipped to do and accomplish this [7].

Now there have been studies applying machine learning to the biomedical field [8–10], however, while we rejoice in the medical advances brought about by technology, we have to admit that there is a certain degree of limitation in the existing studies. First, some studies only incorporate a single dataset, which will inevitably introduce a certain amount of data bias. Second, there are multiple algorithms in machine learning, and some studies use only a single algorithm, which may lead to a certain amount of error. Finally, some studies only perform analytical predictions without corresponding experimental validation, which will lead us to not know the accuracy of the prediction in time.

In this study, we used multiple datasets including GSE58294 and GSE22255 as the discovery set, GSE16561, GSE37587 and GSE110993 as the validation set, and GSE174574 for further single-cell level validation. The ceRNA network associated with DEGs was further constructed using online databases and tools such as the R package. Meanwhile, DEGs were enriched and analysed using multiple methods such as GO, KEGG and GSEA to reveal underlying biological processes and pathways. To further investigate immune-related signatures, the study employed a comprehensive strategy including DEGs, LASSO regression model, SVM, and immune-related genes to identify better immune-related signatures and confirm their ability to distinguish IS from controls using ROC. We also used a mouse model of middle cerebral artery occlusion for corresponding RT-qPCR, western blot, immunofluorescence validation, and the CIBERSORT technique to study immune infiltration features, thus further investigating the role of immunity in the stroke process. Additionally, the study used single cell analysis techniques to reveal precise transcriptional changes during disease progression. Taken together, this research may trigger new insights into the pathogenesis of stroke, thus further enriching the understanding of stroke disease mechanisms and thus providing a theoretical basis for the development of new therapeutic approaches.

## MATERIALS AND METHODS

### Data sources and preprocessing

Datasets (GSE58294; GSE22255; GSE16561; GSE37587; GSE110993; GSE174574) were downloaded from Gene Expression Omnibus (GEO) (https://www.ncbi.nlm.nih.gov/geo/). It’s worth noting that GSE58294, GSE22255, GSE16561, GSE37587 are expression profiling datasets by array. GSE110993 is RNA profiling by high throughput sequencing. GSE174574 is expression profiling by high throughput sequencing. As discovery datasets, GSE58294 and GSE22255 are part of the same GPL570 array platform (HG-U133_Plus_2) Affymetrix Human Genome U133 Plus 2.0. With GSE58294 contains a sample of 69 IS patients and 23 controls [11]. GSE22255 contains 20 IS samples and 20 gender and age matched samples [12]. As validation datasets, GSE16561 and GSE37587 are part of the GPL6883 (Illumina HumanRef-8 v3.0 expression beadchip), with GSE16561 containing 39 IS patient samples and 24 healthy controls [13], GSE37587 containing 68 IS patient samples [14]. As validation datasets, GSE110993 are part of the GPL15456 (Illumina HiScanSQ), with totally 20 ischemic stroke patients and 20 matched healthy controls [15]. As validation datasets, GSE174574 are part of the GPL21103 (Illumina HiSeq 4000) with 3 mouses middle cerebral artery occlusion samples and 3 sham samples [16]. For more detailed information on the six datasets, please refer to Table 1, as all datasets are publicly available, ethics committee approval is not required. The data source for this study was primarily from the GEO database and the data was analysed using R software (version 4.2.2). Before combining the samples, we carried out sufficient quality control. Probe level data were normalised and background corrected to gene expression values, using the average expression value of multiple probes in a gene as the gene expression value. We used Combat functions from the SVA and R packages to eliminate batch differences [17]. The GPL570-based data were grouped to include 89 stroke patients and 43 healthy controls, while the GPL6883-based data included 107 stroke patients and 24 healthy controls. The principal component analysis (PCA) method allowed us to observe the distribution patterns between the disease and control samples, further analysing the data and providing a more reliable data base for the study. We also placed the corrected before and after box plots and UMAP plots in Supplementary Figure 1.

**Table 1:** Basic information of gene expression profiling.

**GEO Accession ID**	**Platform**	**Examples**	**Number of cases**	**Number of controls**	**Year**
**Discovery set**					
GSE58294	GPL570	Blood samples (92)	69 ischemic stroke patients	23 controls	2014
GSE22255	GPL570	Peripheral blood mononuclear cells (40)	20 ischemic stroke patients	20 sex- and age- matched controls	2011
**Validation set**					
GSE16561	GPL6883	Blood samples (63)	39 ischemic stroke patients	24 healthy controls	2010
GSE37587	GPL6883	Blood samples (68)	68 ischemic stroke patients		2015
GSE110993	GPL15456	Plasma samples (40)	20 ischemic stroke patients	20 matched healthy control subjects	2018
GSE174574	GPL21103	Brain tissues of mice (6)	3 middle cerebral artery occlusion samples	3 sham samples	2021

### DEmRNAs, DEmiRNAs, and DElncRNAs identified in IS

For difference analysis, we used the Linear Model for Microarray Data (LIMMA) method in the R′Bioconductor package after normalization and log_2_ conversion. A gene with a P-value of 0.05 and a Fold change of 1.5 was considered differentially expressed. In the data prediction process, the GSE58294, GSE22255 datasets were used to recognize differentially expressed lncRNAs (DElncRNAs) and the Diana database (DIANA-LncBase - Database Commons (cncb.ac.cn)) was used to predict potential miRNAs corresponding to DElncRNAs. In the target prediction process, the “multiMiR” package (v1.16.0) [18] is used to acquire downstream target mRNAs of the miRNAs and find the overlap with the DEmRNAs as the final DEGs. For data visualisation, in R, heat maps and volcano maps can be generated using the “pheatmap” package (v1.0.12), while the use of Venn diagrams can better reveal the prediction process. DEmiRNAs are identified in GSE110993.

### IS triple ceRNA network construction

We constructed an IS-based triple ceRNA network designed to reveal potential regulatory mechanisms between lncRNAs [19, 20], miRNAs and mRNAs; to build this network, we used the online database Diana and R package (multiMiR) to integrate lncRNA-miRNA pairs and miRNA-mRNA pairs and combine them to form an integrated ceRNA network structure. To better represent this network, we used cytoscape (v3.8.0) [21] for visualisation, showing the topology of the ceRNA network, key nodes and other information, thus contributing to a better understanding of the network and the complexity of the internal structure.

### GO, KEGG and GSEA enrichment analyses

In this study, we used “ggplot2 (v3.3.0)”, “clusterProfiler (v3.14.3)”, “org.hs.eg.db (v3.10.0)” and “enrichplot (v1.6.1)” in R software for GO, KEGG and GSEA pathway enrichment analysis. In addition [22, 23], we set thresholds of P<0.05 and Q<0.05 to screen against significantly enriched GO and KEGG entries. A GSEA approach [24] was used, based on the KEGG database for the analysis. During the enrichment analysis, we used the Normalised Enrichment Scale (NES) [25] to identify pathways that were up- or down-regulated in stroke samples compared to normal subjects. These results were ultimately used to present GSEA enrichment maps.

### Selection of the better DEIRG

LASSO regression [26] is a method for dimensionality reduction in the analysis of large-scale gene expression data to avoid overfitting. The method removes noisy features and retains meaningful gene features by introducing a penalty parameter (λ). To select the best penalty parameter, we use a five-fold cross-validation method for optimisation. In addition to this, the R package “glmnet (v4.1-2)” was used, a tool that removes genes that may have overfit the model and further optimises model performance. In a support vector machine (SVM), we set the parameters: kernel=“radial”, type=“eps-regression”, cross=5 to find the best variable [27]. In addition, 17,664 immune-related genes (See Supplementary Table 10 for more details) have been collated from the GeneCards website (v5.7, https://www.genecards.org), which can be effectively used for immune response-related analysis. Therefore, the LASSO regression model, SVM, DEGs, and immune-related gene sets will be evaluated together to find better differentially expressed immune response-related signatures (DEIRS) [28, 29].

### Discriminant ability evaluation and validation

To assess the ability of DEIRS to discriminate between stroke and healthy populations, we used the “ROCR (v1.0-11)” R package to plot ROC curves and visualize the AUC area (ID3 AUC=0.893, 95% CI=0.819-0.954; SLC22A4 AUC=0.891, 95% CI=0.819-0.951) of DEIRS in the discovery set. In addition, we validated this performance (ID3 AUC=0.898, CI=0.830-0.951; SLC22A4 AUC=0.940, 95%=0.874-0.988) using the validation set. Also, to test the accuracy of the LASSO model, we plotted ROC curves to assess its precision in the discovery set (AUC=0.955, 95% CI=0.901-0.992) and validation set (AUC=0.813, 95% CI=0.737-0.889). In addition, to test the accuracy of the SVM model, we also plotted ROC curves to assess its accuracy in the discovery set.

### Analysis of immune infiltration

CIBERSORT is a commonly used deconvolution algorithm that translates gene expression matrices into the composition of infiltrating immune cells [6]. We performed a CIBERSORT evaluation on the discovery dataset. In this study, the infiltrating immune cell composition of each sample was assessed by the CIBERSORT algorithm. The immune cells covered 22 types, including T cells, B cells, NK cells, monocytes, macrophages, dendritic cells, mast cells, eosinophils, and neutrophils. The scores of all 22 immune cell types were assessed in each sample sum to one, meaning that the relative proportions of each cell type can be compared and counted against each other.

### Animals and middle cerebral artery occlusion (MCAO) *in vivo* model

We purchased adult male C57NL/6 mice from SPF Biotechnology Co., Ltd. (Beijing, China). In Tianjin, China, the mice were housed in the Animal Experimental Center of the Fifth Central Hospital at a temperature of 20–25° C and humidity of 50% ± 5%. Firstly, a longitudinal incision of approximately 1 cm in length was made between the sternum and mandible of the mouse to allow for surgical manipulation through a stereomicroscope (Olympus Corporation, Tokyo, Japan). Next, we isolated the right common carotid artery and identified the external and internal carotids further. We then ligated the distal and proximal ends of the external carotid artery. Lastly, a modified nylon thread (silicone tip length 3-4 mm, silicone tip diameter 0.22-0.23 mm, thread body diameter 0.1 mm, total thread length 30 mm) was inserted from the carotid artery into the middle cerebral artery (length 10 ± 0.5 mm) and secured with surgical thread [30]. We divided all mice into MCAO and Sham groups, each with six mice.

### Immunofluorescent staining

The animals were anaesthetised and killed 24 h after the MCAO surgery. Brain tissue was submerged in 4% paraformaldehyde solution, fixed at 4° C for 24 h, dehydrated and paraffin-embedded. The brain was continuously sectioned (4 μm coronal section) at 1.0 to 5.0 mm posterior to bregma. 1 h in an oven at 80° C. Dewaxing with xylene for 5 min, 3 times. Gradient alcohol dehydration (anhydrous ethanol, 95% ethanol, 75%, 50%). Immersion in ultrapure water for 2 min. Soak in tap water for 2 min. Antigen repair by sodium citrate, autoclave heat repair for 5 min. Cooled for 1 h to room temperature. 0.3% Triton X-100 penetration for 10 min. PBST rinse for 5 min, 3 times. 10% BSA (No. A8010, Solarbio, China) blocked for 1 h. PBS rinse for 5 min, 3 times. Overnight incubation with primary antibody (ID3, No. MG750811, 1:500, mouse, Abmart, China; SLC22A4, No.TD9724, 1:500, rabbit, Abmart; NeuN, ab177487, 1:500, rabbit, Abcam, UK; Nestin, ab5320, 1:500, mouse, Abcam). Incubation with fluorescent secondary antibody for 1 h the next day. (Alexa Fluor® 488, ab150113, 1:500, Goat Anti-Mouse, Abcam; Alexa Fluor® 594, ab150080, 1:500, Goat Anti-Rabbit, Abcam). Sealing with a DAPI-containing sealing solution. Under a confocal laser scanning microscope (Olympus, Tokyo, Japan), slides were observed. Imaging and analysis are carried out using ZEN software.

### Real time quantitative PCR

RNAiso Plus (No. SD1412, Takara, Japan) was used to isolate total RNA from frozen brain tissue, followed by reverse transcription kits (No. AT351, TransGen Biotech, China) to synthesize cDNA. Next, the synthesised cDNA was amplified using a two-step qRT-PCR kit (AQ202, TransGen Biotech) to detect the gene of interest. Finally, to eliminate variability between samples, Using the 2-ΔΔC method, gene expression levels were normalised to U6. In Supplementary Table 1, primers are listed.

### Western blot

Frozen brain tissue was lysed with RIPA working solution mixed with protease inhibitors (No. ST505, Beyotime, China). Centrifuge for 15 minutes at 14,000 rpm (4° C). The supernatant was collected, a small portion was taken, and the concentration was determined using the BCA kit. (No. P0012S, Beyotime, China). After mixing with 4x loading buffer, the remaining protein was boiled for 10 minutes. Electrophoresed on 10%-12% polyacrylamide gels, then transferred the protein to a 0.45 um PVDF membrane. Using 5% skim milk powder (No. BS102-100g, Biosharp, China) to block the membrane for 1 h. Then the membrane was incubated with ID3 antibody (No. MG750811, 1:1000, mouse, Abmart), SLC22A4 antibody (No. TD9724, 1:1000, rabbit, Abmart) for overnight, using anti-GAPDH antibody (No. ab8245, 1:10000, mouse, Abcam) as an internal loading control. Recovered the primary antibody, wash with TBST for 15 minutes three times. Then incubated the secondary antibody (room temperature) for 1 h. Recovered the secondary antibody. Washed the membrane with TBST for 15 minutes three times. Finally, add ECL exposure solution (No. P0018S, Beyotime, China) for exposure. Using ImageJ software (v1.46r, National Institutes of Health, USA), we determined the relative levels of protein expression.

### Single-cell analyses

Single-cell sequencing data were obtained from GSE174574 on the GEO website [16]. 3 brain cortex samples of a control group and 3 samples under 24-hour MCAO condition were used to generate data. Seurat (4.3.0) and R (4.2.2) were used to import the original data. Standard quality control and normalization procedures were applied to all of these data. We removed low-quality cells (<200 genes/cell and >10% mitochondrial genes). The Harmony algorithm was used to integrate datasets from 6 samples in the entire database. To identify highly variable genes, we used the Seurat function “FindVariableFeatures”. To integrate the data, we chose the top 2000 genes exhibiting the greatest cell-to-cell variation. For data scaling and principal component analysis (PCA), the dataset underwent processing using the “ScaleData” and “RunPCA” functions, respectively. Using the “FindNeighbors” and “FindClusters” functions, subsequent auto-clustering analyses were performed. UMAP scatter plots were utilized to visualize the resulting clustering outcomes.

### Statistical analyses

We performed statistical analyses using R software and GraphPad Prism software, setting the conditions for differential expression analysis to p < 0.05, and |log_2_ fold change (FC)| > 0.585. We refer to expressions with log_2_FC greater than 0.585 as up-regulated expressions, and expressions with log_2_FC less than 0.585 as down-regulated expressions. We used t-test to analyze normally distributed variables and used the Mann-Whitney U-test to evaluate nonnormally distributed variables. We showed data as mean and standard error (SEM). When the p-value is less than 0.05, we consider this to be a sign of a significant difference.

### Data availability statement

The data that support the findings of this study are available in GEO database, reference number GSE58294, GSE22255, GSE16561, GSE37587, GSE110993 and GSE174574. The datasets during and/or analyzed during the current study are available from the corresponding author on reasonable request.

## RESULTS

### Data pre-processing

**Figure 1:**
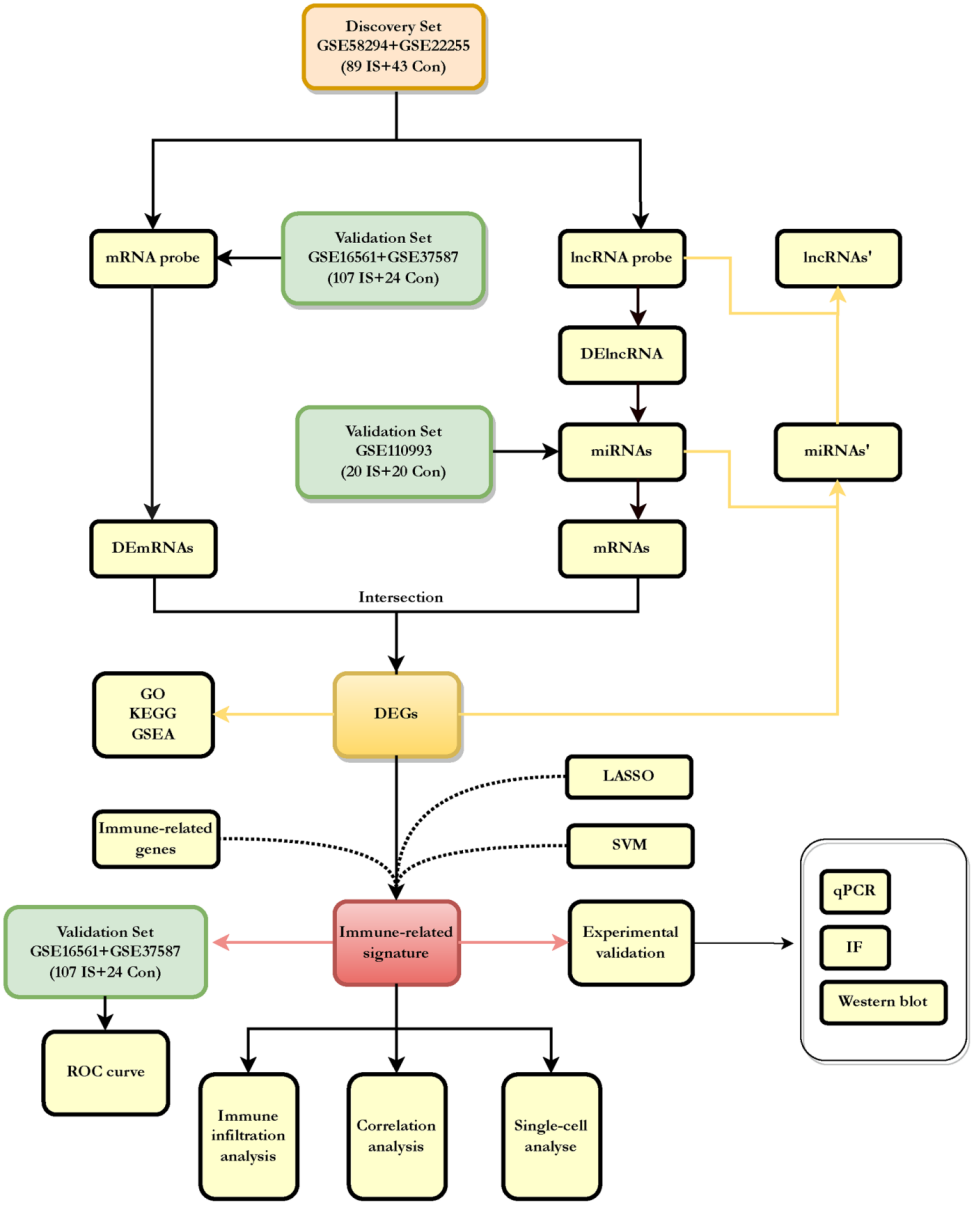
**Flowchart about the entire working processes of this study.** IS: ischemic stroke; Con: controls; DEGs: differently expressed genes.

PCA scatter plots show two distinct distribution patterns between ischemic stroke patients and healthy controls after batch correction with ComBat. As shown in the Supplementary Figure 1, samples from ischemic stroke patients were mostly distributed on the left-hand side of the plot, while samples from healthy controls were predominantly distributed to the right. The six downloaded GEO datasets are shown in Table 1, where GSE58294 and GSE22255 were combined as the discovery dataset comprising 89 IS patients and 43 healthy controls, and GSE16561 and GSE37587 were combined as the validation dataset comprising 107 IS patients and 24 healthy controls. GSE110993 was the same validation dataset comprising 20 IS patients and 20 matched healthy control subjects. GSE174574 for single cell level analysis, including 3 MCAO samples and 3 sham control samples. After data merging and eliminating differences between batches, we obtained expression matrices for 132 samples in the discovery dataset (GSE58294, GSE22255), 131 samples in the validation dataset (GSE16561, GSE37587) and 40 samples in the validation dataset (GSE110993). The workflow diagram is shown in Figure 1.

### Identification of DEmRNAs

**Figure 2:**
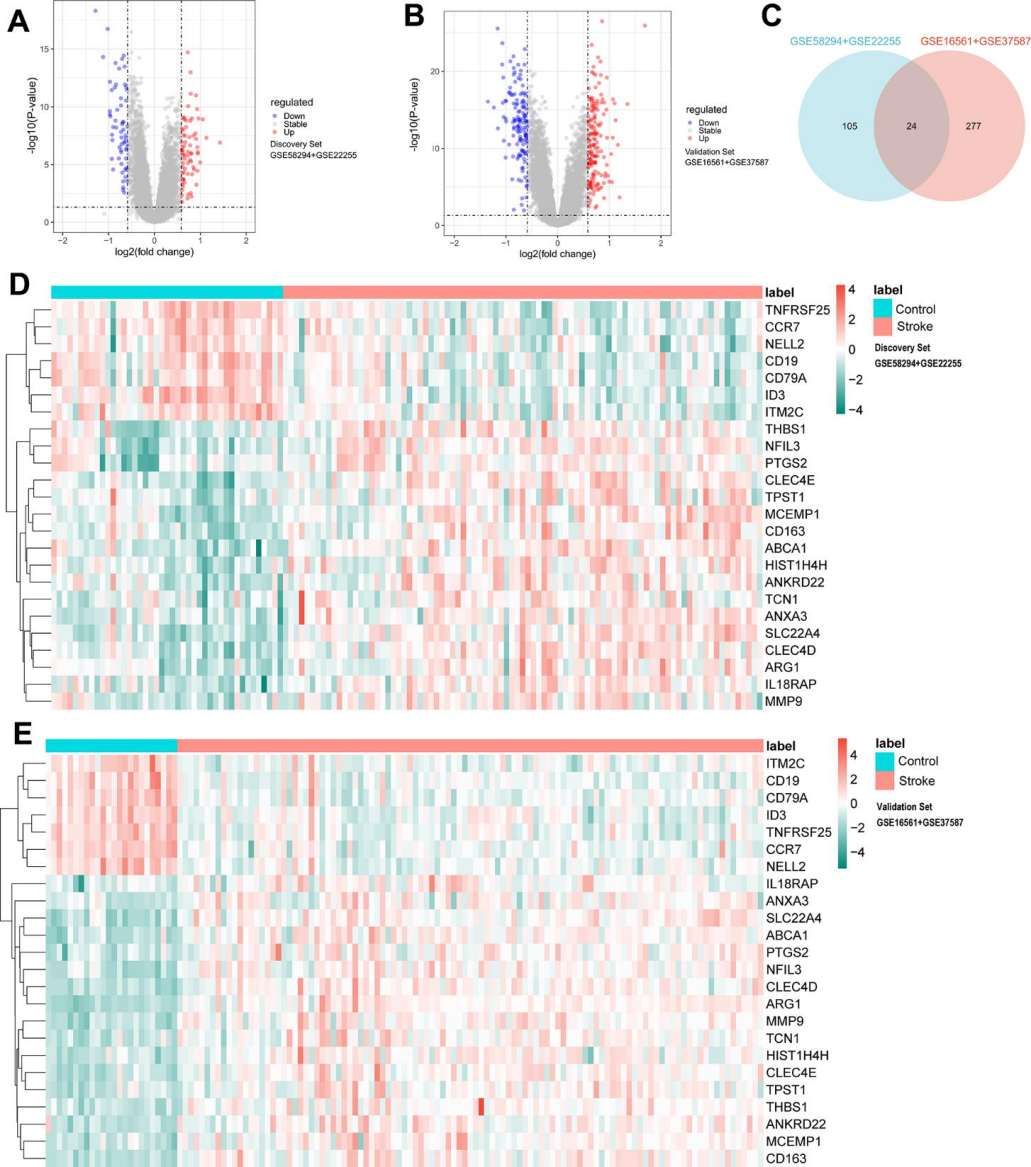
**Difference analysis of identification criteria: P < 0.05 and |log_2_FC| >0.585.** (**A**) Volcano plots for DEmRNAs in discovery set (71 upregulated and 60 downregulated). (**B**) Volcano plots for DEmRNAs in validation set (166 upregulated and 135 downregulated). (**C**) Venn diagram showing the common 24 DEmRNAs between discovery set and validation set. (**D**) Heatmap plot showing the common 24 DEmRNAs in discovery set. (**E**) Heatmap plot showing the common 24 DEmRNAs in validation set.

The limiting thresholds were p < 0.05 and |log_2_FC| >0.585, and 131 DEmRNAs were selected for further analysis (71 up-regulated, 60 down-regulated) in the discovery datasets; the volcano plot is shown in Figure 2A. Under the same threshold conditions, a total of 301 DEmRNAs (166 up-regulated genes, 135 down-regulated genes) were found in the validation datasets; the volcano plot is shown in Figure 2B, and the intersection of the two was taken to obtain 24 DEmRNAs, as shown in the Venn diagram (Figure 2C), including (ID3, SLC22A4, CLEC4E, CLEC4D, ABCA1, MCEMP1, TNFRSF25, ITM2C, CD19, NFIL3, IL18RAP, ANXA3, CD163, CCR7, HIST1H4H, ANKRD22, THBS1, ARG1, MMP9, CD79A, PTGS2, TCN1, NELL2, TPST1). The heat map is shown in the Figure 2D, 2E.

### Identification of DEmiRNAs, and DElncRNAs

**Figure 3:**
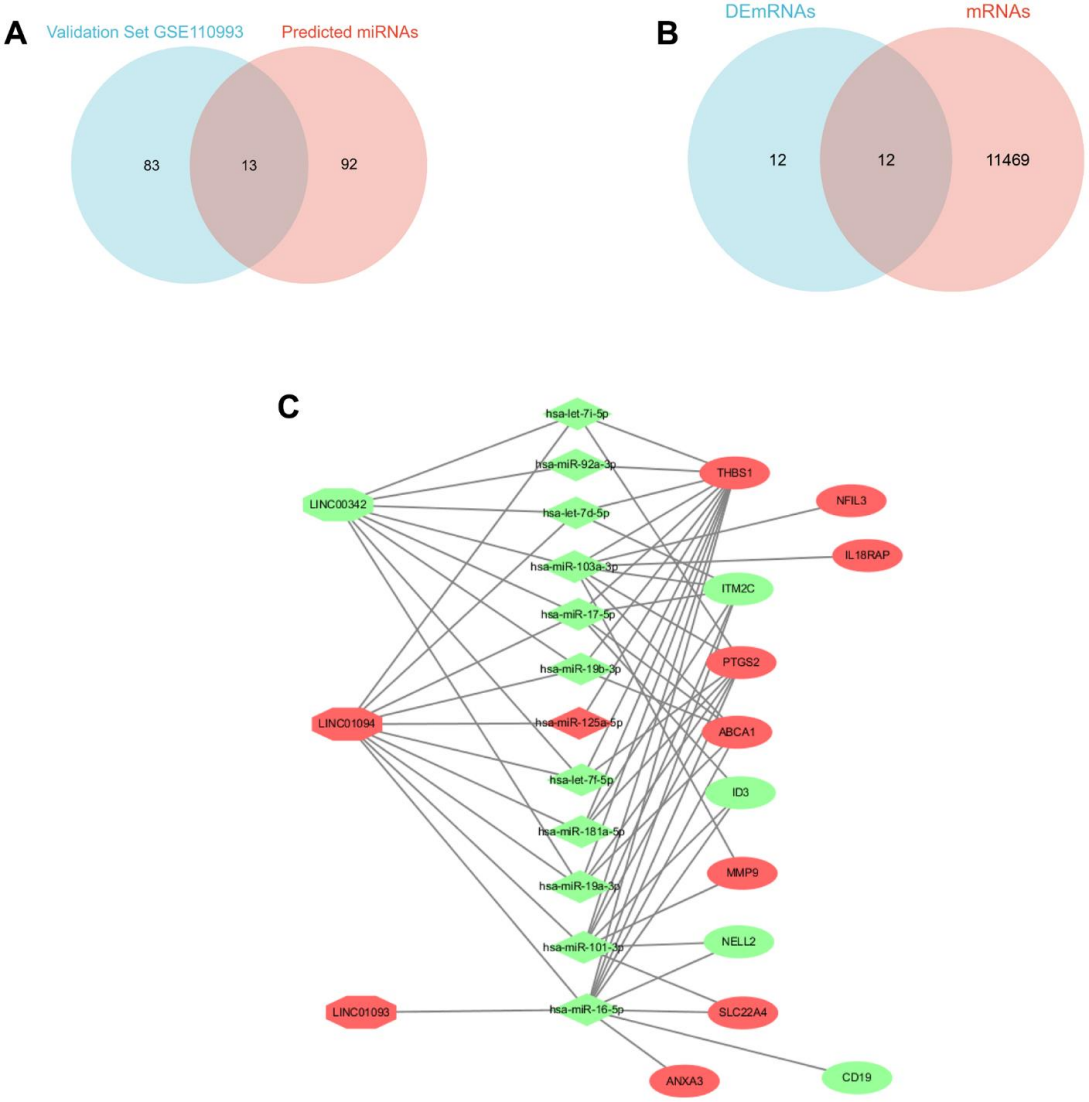
**Triple ceRNA network construction.** (**A**) Venn diagram showing the common miRNAs between validation set GSE110993 and predicted miRNAs. (**B**) Venn diagram showing the DEGs between DEmRNAs and mRNAs. (**C**) CeRNA network in IS, the octagon represents lncRNA, the diamond represents miRNAs, and the ellipse represents mRNAs. (red represents upregulated, and green represents downregulated).

In discovery sets, a total of six DElncRNAs were directly identified, as detailed in the Supplementary Table 2, including two down-regulated lncRNAs and four up-regulated lncRNAs, and 105 potential miRNAs, which can be checked in the Supplementary Table 3, detailed in the Diana database were found to bind to these six lncRNAs. In the validation set GSE110993, 16 up-regulated (e.g. miR-125a-5p) and 80 down-regulated DEmiRNAs (e.g. miR-101-3p) were identified. The intersection of the two was taken to yield 13 potential candidate miRNAs, (Figure 3A) miRNAs can be detailed in the Supplementary Table 4. Based on the multiMiR R package, these 13 potential candidate miRNAs were predicted to bind to 11,481 downstream mRNAs, and after intersection with DEmRNAs (Figure 3B), the focus was on the remaining 12 DEGs, including (PTGS2, ID3, ITM2C, MMP9, NELL2, SLC22A4 THBS1, ABCA1, ANXA3, CD19, IL18RAP, NFIL3), which shared genes between 11481 target mRNAs and 24 DEmRNAs. However, not all of the 13 potential candidate miRNAs can match each other with the 12 screened DEGs, and in order to focus more on the interactions with them. The 12 DEGs were reverse predicted by the multiMiR R package and intersected with the 13 potential candidate miRNAs mentioned above to obtain 12 miRNAs’ that were strongly associated with DEGs. Similarly, by Diana database and taking intersection with the above 6 DElncRNAs, 3 lncRNAs’ strongly associated with DEGs were obtained. lncRNAs’, miRNAs’, and the relationship of DEGs are shown in the Figure 3C.

### GSEA, KEGG and GO enrichment analysis

**Figure 4:**
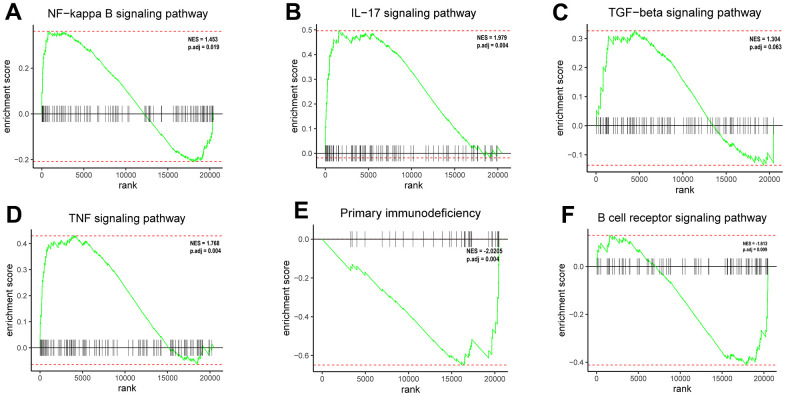
**Enrichment plots from GSEA.** (**A**) The DEGs positively correlated with the NF-κB signaling pathway. (**B**) The DEGs positively correlated with the IL-17 signaling pathway. (**C**) The DEGs positively correlated with the TGF-β signaling pathway. (**D**) The DEGs positively correlated with the TNF signaling pathway. (**E**) The DEGs negatively correlated with the primary immunodeficiency signaling pathway. (**F**) The DEGs negatively correlated with the B cell receptor signaling pathway.

Based on GSEA enrichment results, the 12 DEGs involved in the ceRNA network were mostly concentrated in: NF-κB signaling pathway, IL -17signaling pathway, TGF-β signaling pathway, TNF signaling pathway, Primary immunodeficiency, B cell receptor signaling pathway (Figure 4A–4F). The KEGG enrichment results are mainly focused on: IL-17 signaling pathway, TNF signaling pathway and so on (See Supplementary Table 5 for details). The results of the GO enrichment analysis were mainly: regulation of neuroinflammatory response, carboxylic acid transmembrane transport etc. (See Supplementary Table 6 for details). Notably, the results GSEA clearly suggested immune-related pathways, which pointed the way to the next analysis.

### Selection of the better DEIRS in IS

**Figure 5:**
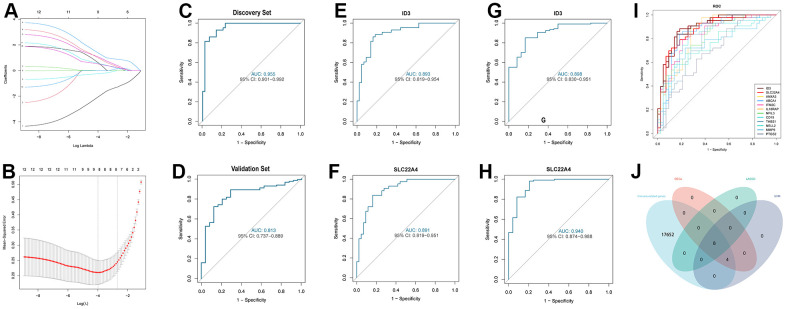
**Comprehensive strategy to select the better DEIRG in IS.** (**A**) 12 differentially expressed genes are represented by LASSO coefficient profiles. (**B**) Twelve differentially expressed genes were examined for binomial deviance profiles. (**C**) ROC curve for analysing LASSO regression model accuracy in the discovery set. (**D**) ROC curve for analysing LASSO regression model accuracy in the validation set. (**E**) ROC curve for ID3 in the discovery set. (**F**) ROC curve for SLC22A4 in the discovery set. (**G**) ROC curve for ID3 in the validation set. (**H**) ROC curve for SLC22A4 in the validation set. (**I**) ROC curve for SVM model accuracy in the discovery set. (**J**) Venn diagram for showing a comprehensive strategy among DEGs (pink circle), LASSO regression (light green circle), SVM models (purple circle), immune-related genes (light blue circle). DEIRS: differentially expressed immune-related signatures.

We identified 12 DEGs in the discovery set. To select the better DEIRS in IS, we used a comprehensive strategy among DEGs, LASSO, SVM and immune-related genes. 12 differential genes were incorporated in the LASSO regression model, and 8 candidate genes were obtained after dimensionality reduction (Figure 5A, 5B). Subsequently, we evaluated the accuracy of the LASSO regression model in the discovery and validation sets by ROC curves (Figure 5C, 5D). The SVM incorporated 12 differential genes and evaluated the model accuracy by ROC curves (Figure 5H), AUC values, 95% CIs are shown in the Supplementary Table 7. The intersection between LASSO regression analysis, SVM, DEGs, and immune-related genes resulted in 8 immune-related signatures (Figure 5I). Notably, among these eight immune-related signatures, ID3 was the smallest among the negative values in the LASSO regression score and SLC22A4 was the largest among the positive values in the LASSO regression score. Also, ID3 and SLC22A4 had the top two AUC values in the SVM. Therefore, ID3 and SLC22A4 were selected as the better DEIRS for further analysis. We then assessed ID3 and SLC22A4’s discriminatory ability for stroke patients and healthy controls, respectively, and a good discriminatory ability was found for both the discovery and validation sets (discovery set: ID3 AUC=0.893, 95%CI=0.819-0.954; SLC22A4 AUC=0.891, 95%CI=0.819-0.951; validation set ID3 AUC=0.898, 95%CI=0.830-0.951; SLC22A4 AUC=0.940, 95%CI=0.874-0.988) (Figure 5E–5H).

### Expression validation of ID3 and SLC22A4

**Figure 6:**
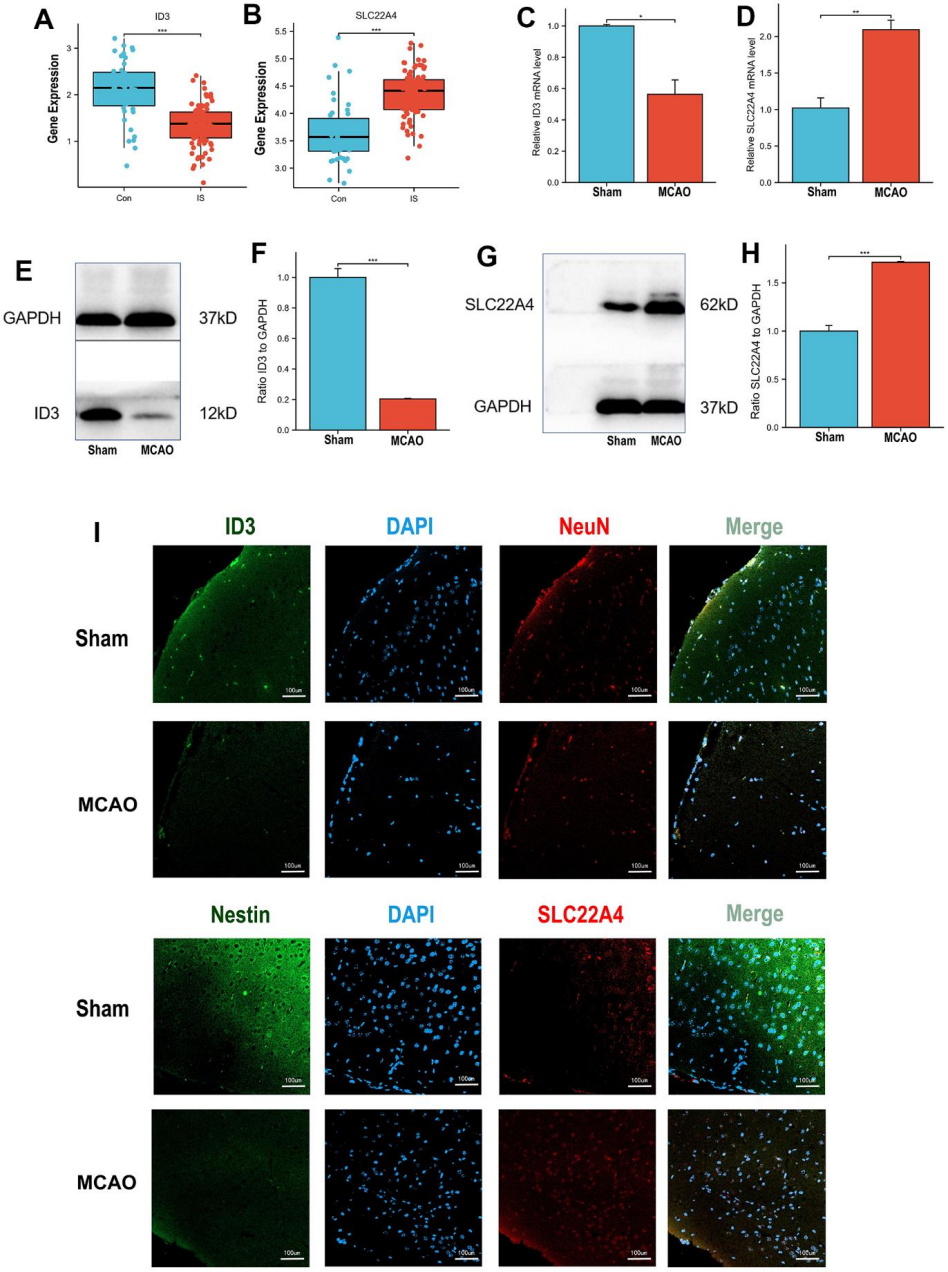
**Expression validation *in vivo* models.** (**A**) ID3 expression patterns in IS patients and controls in discovery set. (**B**) SLC22A4 expression patterns in IS patients and controls in discovery set. (**C**) The mRNA levels of ID3 in mouse brain tissues. (**D**) The mRNA levels of SLC22A4 in mouse brain tissues. (**E**, **F**) The protein levels of ID3 in mouse brain tissues. (**G**, **H**) The protein levels of SLC22A4 in mouse brain tissues. (**I**) The immunofluorescence levels of ID3 and SLC22A4 in mouse brain tissues. MCAO group and sham group, number of mice per group n=6.

Analysis of the discovery dataset (GSE58294, GSE22255) showed a significant decrease in ID3 expression and a significant upregulation of SLC22A4 expression in stroke patient samples compared with controls (Figure 6A, 6B). We then used the mouse MCAO model and examined the mRNA and protein expression levels of ID3 and SLC22A4 and found that ID3 was significantly downregulated and SLC22A4 was significantly upregulated in the MCAO group (Figure 6C–6H). Immunofluorescence detection of ID3 and SLC22A4 expression revealed that the fluorescence intensity of ID3 was weakened and that of SLC22A4 was enhanced in the MCAO group (Figure 6I).

### Immune infiltration analysis

**Figure 7:**
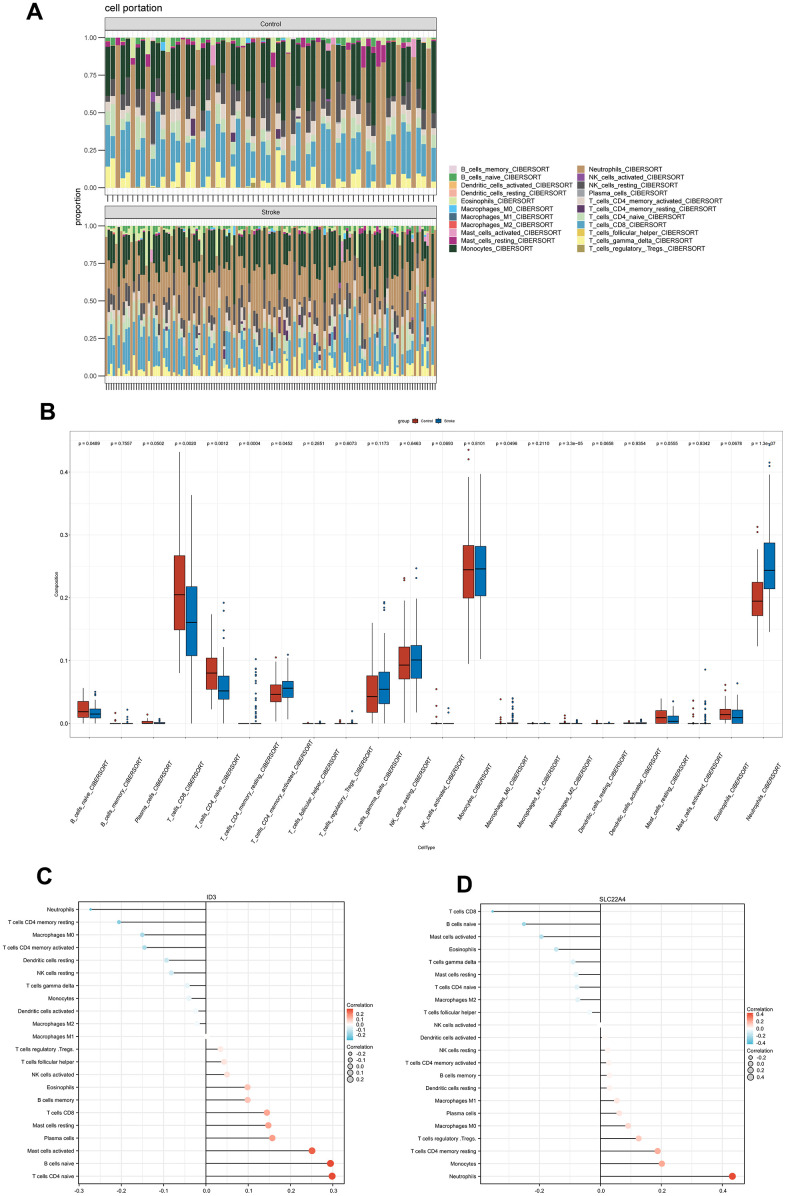
**Immune infiltration characteristics.** (**A**) A bar plot shows the relative percentage of 22 immune cell subsets. (**B**) Comparison of immune cells infiltrating IS patients and controls. (**C**, **D**) A Spearman correlation of immune cell subsets and ID3; SLC22A4. The color and size of the dots indicate the strength of the correlation.

IS is followed by a series of immune events, including peripheral immune cell invasion, pro-inflammatory factors secretion and so on [31]. Thus, we compared immune infiltration characteristics between IS groups and normal groups by using CIBERSORT. Bar charts depicted relative percentages of different immune cell subpopulations in each sample. (Figure 7A). CD8 T cells, naive B cells, and naive CD4 T cells show lower levels of immune infiltration in IS patients than in controls, while activated memory CD4 T cells and neutrophils show higher levels of immune infiltration (Figure 7B). An analysis of Spearman correlation was performed to demonstrate the relationship between ID3, SLC22A4 and different immune cell subpopulations. According to these results, ID3 correlated positively with CD8 T cells, naive B cells, and naive CD4 T cells; however, it correlated negatively with activated memory CD4 and neutrophils. CD8 T cells, naive B cells, and naive CD4 T cells were negatively correlated with SLC22A4, while activated memory CD4 cells, neutrophils were positively correlated (Figure 7C, 7D). See Supplementary Tables 8, 9 for more details.

### Single cell genome sequencing level analysis

**Figure 8:**
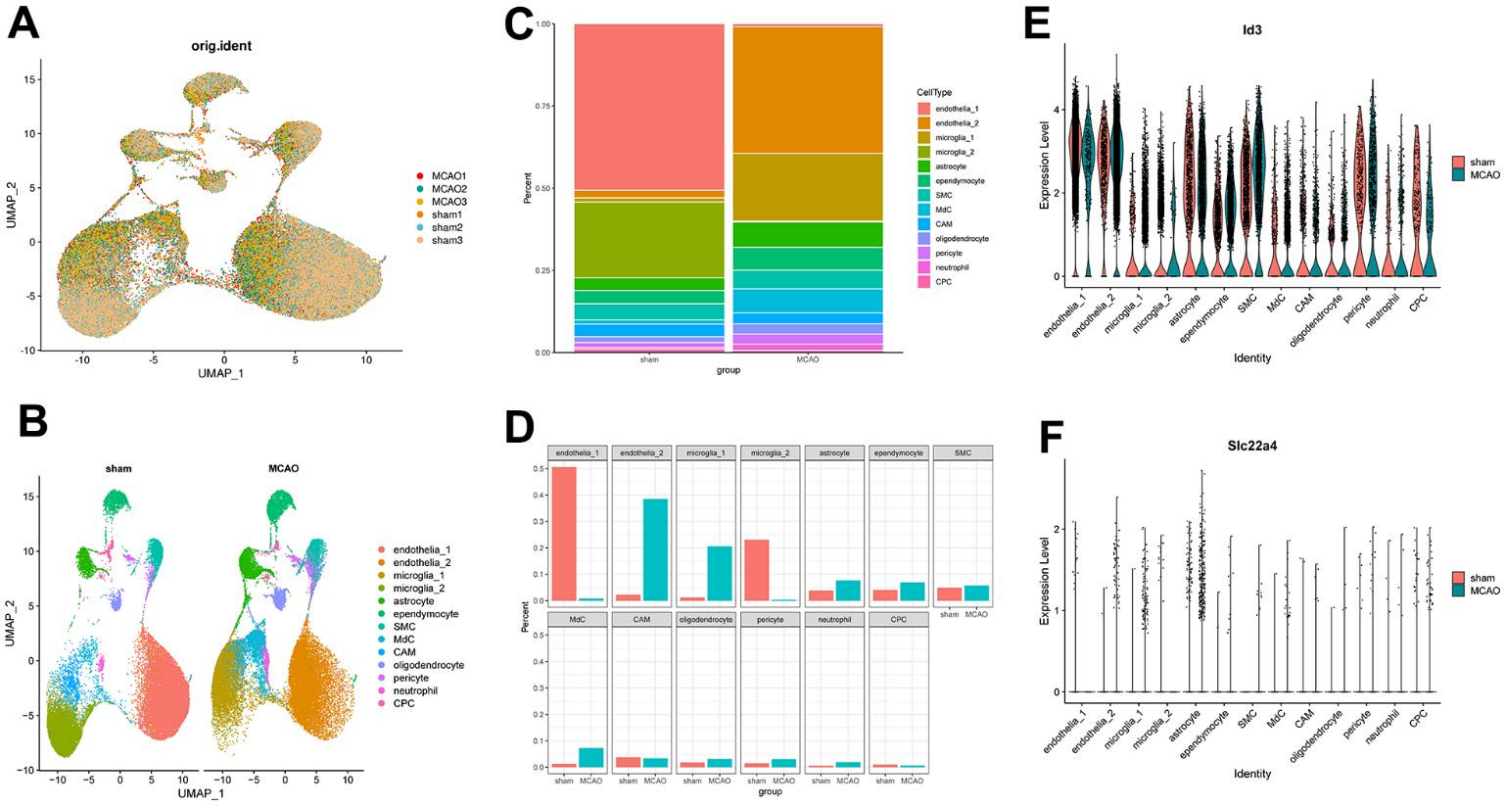
**Mouse brains’ scRNA-seq demonstrates transcriptome atlas.** (**A**) UMAP plot for visualizing clustering profiles between MCAO and sham groups. (**B**) The UMAP plots display clustering of single cells by types. (**C**) The proportion of cells in each sample for each cluster is shown in a bar plot. (**D**) Visualizing the number of cells in each sample for each cluster. (**E**, **F**) Violin plots showing ID3 and SLC22A4 expression levels in the above 13 clusters.

In order to examine MCAO-induced changes in gene expression profiles and cell heterogeneity, we downloaded GSE174574 from the GEO database for further analysis. GSE174574 includes three mouse middle cerebral artery occlusion model samples and three control samples, as detailed in Table 1. Significant heterogeneity was seen in the MCAO group relative to the sham group in UMAP (Figure 8A). Detecting known cell type markers, we identified 13 transcriptionally distinct clusters including endothelia_1; endothelia_2; microglia_1; microglia_2; astrocyte; ependymocyte; vascular smooth muscle cells (SMC); monocyte-derived cells (MdC); central nervous system (CNS)-associated macrophages (CAM); oligodendrocyte; pericyte; neutrophil; choroid plexus capillary endothelial cells (CPC) (Figure 8B). We compared and visualized the composition ratios of each of the cell types identified in sham and MCAO groups to identify brain cell types susceptible to ischemic injury. We found a significant decrease in endothelial_1 cells and a significant increase in endothelial_2 cells in MCAO group; interestingly, there was a significant increase in microglia_1 cells and a significant decrease in microglia_2 cells (Figure 8C, 8D). We examined the expression of ID3 and SLC22A4 in 13 identified clusters and found that ID3 expression was reduced in endothelial cell subtype I and increased in endothelial cell subtype II in MCAO group, while ID3 was increased in microglia subtype I and decreased in microglia subtype II; SLC22A4 was mainly concentrated in astrocytes (Figure 8E, 8F).

### Correlation analysis based on the single cell level

**Figure 9:**
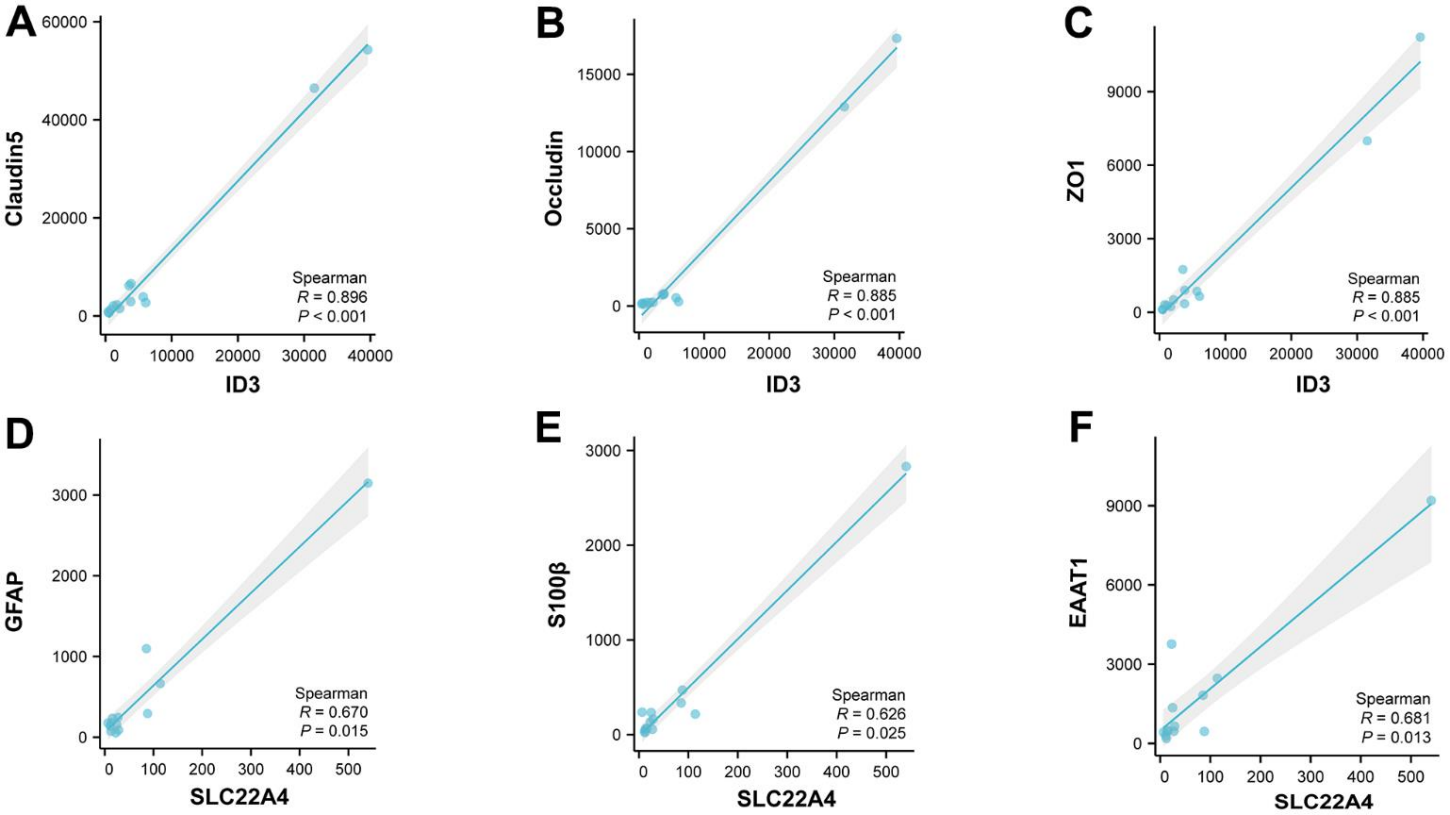
**Correlation analysis based on the single cell level.** (**A**–**C**) Correlation analysis of ID3 with Claudin5, Occludin and ZO1 in vascular endothelial cells. (**D**–**F**) Correlation analysis of SLC22A4 with GFAP, S100β and EAAT1 in astrocytes.

Based on single cell level data, we performed correlation analysis between ID3 and vascular endothelial cell marker [32]; SLC22A4 and astrocyte marker [33]. We found that in endothelial cells, ID3 was significantly and positively correlated with Claudin5, Occludin, and ZO1 (Figure 9A–9C); in astrocytes, SLC22A4 was significantly and positively correlated with GFAP, S100β, and EAAT1 (Figure 9D–9F).

## DISCUSSION

In the United States, stroke is the second leading cause of death and disability, affecting one in four people during their lifetime [34]. Emerging molecular and diagnostic technologies are being developed rapidly to better treat IS patients, while early diagnosis, treatment, and prognosis of IS still have limitations and require further research. Immune infiltration is one of the key factors in the development of IS, but few studies have explored immune infiltration in depth as a signature of IS progression [5]. Furthermore, the mechanism of immune-related signatures as a ceRNA may provide a new direction of thinking for early diagnosis and precise treatment of IS, which needs further in-depth study.

We successfully constructed a network containing triple ceRNAs and identified 12 differentially expressed genes (DEGs). Then we comprehensively evaluated the Venn results between DEGs, LASSO regression, SVM, and immune-related genes and selected two better signatures -- ID3 and SLC22A4. The CIBERSORT algorithm showed that the characteristics of immune infiltration in IS. We also explored the involvement of ID3 and SLC22A4 in immune infiltration, and their expression in a mouse model of middle cerebral artery occlusion and at the single cell level, respectively.

Inhibitor of DNA binding 3(ID3, also known as HEIR-1; bHLHb25, gene ID: 3399) is located on chromosome 1p36.12(exon count :3) [35]. As a member of the DNA binding inhibitor family, ID3 plays a crucial role in cell growth, self-renewal, senescence, angiogenesis and neurogenesis, and plays an integral role in functions such as stress coping, neuroplasticity, and neural circuitry [36–38]. In neurological and behavioural studies, ID3 has demonstrated its biological importance.

ID3 also plays an important role in immune regulation [39, 40]. In the course of our research explorations, we identified the presence of ID3 from the 12 candidate genes identified. During our study explorations further into independent patient populations, we verified the significance of these 12 genes in terms of diagnostic reliability. Notably, ID3 was found to be time-stable in the early 24 hours of stroke in previous study, further highlighting the importance of this gene in the early diagnosis of the disease [38].

Solute carrier family 22 member 4 (SLC22A4, also known as OCTN1; DFNB60, gene ID:6583, HGNC:10,968, OMIM:604,190) is located on chromosome 5q31.1 (encoded by 11 exons). Transporting organic cations between the plasma membranes of epithelial cells [41, 42]. It is associated with genetic polymorphisms that cause inflammation and plays a huge role in the human innate immune response. SLC22A4 polymorphisms are associated with the incidence of inflammatory bowel disease (IBD), Crohn’s disease (CD) and ulcerative colitis (UC) [43–45]. There are two polymorphisms associated with rheumatoid arthritis (RA) in Japanese and Chinese populations, rs2073838 and rs3792876 [46, 47]. SLC22A4 was also found to be significantly overexpressed in RA tissues by experiments in a mouse model of collagen-induced arthritis. At the same time, the running transcription factor RUNX1 regulated the expression of SLC22A4, thus having a significant impact on the susceptibility to RA [48]. Finally, it was shown that by reducing the non-normal transporter function of the SLC22A4 503F variant, we could effectively reduce the over-triggering of the inflammatory response [49]. Furthermore, ischemic stroke in the Japanese population was also significantly associated with the SLC22A4 gene polymorphism rs273909 [50].

In this study, two important signatures closely related to immune infiltration in ischemic stroke were identified. In MCAO model mice, bioinformatics analysis and multidimensional validation by qRT-PCR, western blot and immunofluorescence revealed a decrease in ID3 gene expression and an increase in SLC22A4 gene expression. These findings suggest that ID3 and SLC22A4 have significant biological significance in ischemic stroke.

Additionally, the enrichment analysis demonstrated that DEGs are involved in signaling pathways involved in inflammation or immunity. Specifically, the NF-kB signalling pathway showed significant enrichment in GSEA enrichment analysis, implying that NF-kB plays an essential role in inflammatory and immune responses. In addition, the transcription factor NF-κB is itself a regulator of the inflammatory response [51]. As ischemic stroke (IS) is a disease that elicits a systemic immune response [52], NF-kB plays a huge role in the induced inflammation of this disease. Also, NF-kB overexpression is involved in the inflammatory response associated with rheumatoid arthritis through activation of the SLC22A4 promoter [53]. And ID3 also plays a regulatory role in the NF-kB signalling pathway, its knockdown may lead to dysfunction of this signalling pathway [54]. We find these findings to be consistent with our bioinformatics analysis, suggesting that ID3, SLC22A4 and the NF-kB signaling pathway are very important for mediating biological processes involved in ischemic stroke. These results point the way to our subsequent analysis. However, these signalling pathways and their specific mechanisms of action still require further experimental validation.

On the basis of cumulative evidence, IS triggers a systemic immune response that is not confined to the infarcted area alone. This immune response is a systemic immune inflammatory response triggered to some extent by oxidative stress and immune dysfunction triggered by the ischemic brain tissue [55]. The progressive increase in oxidative mediators leads to further infiltration of immune cells, including T cells, B cells, macrophages and dendritic cells in the ischemic brain areas, thereby exacerbating the neurotoxic and neuroinflammatory response [56]. In order to learn more about the type and proportion of immune cell infiltration in stroke patients with ischemic stroke, 22 immune cells were evaluated using CIBERSORT. According to our analysis, ischemic stroke patients had reduced infiltration of naive B cells, CD8 T cells and naive CD4 T cells, while there was a trend towards increased infiltration of activated CD4 memory T cells, M0 macrophages and neutrophils.

Most experimental stroke models regarded neutrophils as the first blood-derived immune cells [57]. It had been reported that on day one, neutrophils increased, peaked on day three, and then declined, at 7 and 15 days after cerebral ischemia, they were still present, and were positively correlated with infarct volume and functional impairment [58]. In the peripheral blood, Kaito et al. reported an increase in monocytes-macrophages after brain injury [59]. 3-7 days after the onset of ischemia, peripheral blood monocytes and macrophages infiltrate the site of injury and reach a peak [60]. Unlike neutrophils, monocytes and macrophages, the number of lymphocytes in the peripheral blood flow is reduced in patients with ischemic stroke, resulting in an increased neutrophil/lymphocyte ratio [57]. Conversely, some studies showed that IS severely impairs certain stages of B-cell development in the bone marrow and this impairment leads to a reduction in the number of peripheral lymphocytes [61]. When this happens, the CD4+ T cell response shifts from a T cell-mediated immune system to a Th2-mediated humoral immune system, protecting the brain from further damage [62]. However, due to suppression of the immune system, the number of T and B lymphocytes in peripheral blood eventually decreased [63].

But reports about the relationship between different types of immune cells and IS development are lacking, and more research is required to uncover details in the process. In addition, we found a correlation between ID3 and SLC22A4 and 22 different types of immune cells. Among naive B cells, CD8 T cells, and CD4 naive T cells, ID3 was significantly positively correlated and negatively correlated with CD4 activated memory T cells, M0 macrophages and neutrophils. SLC22A4, on the other hand, was negative correlation and positive correlation with M0 macrophages and neutrophils. The role of ID3 and SLC22A4 in IS pathogenesis will thus be an important field for future research.

In addition, to identify brain cell types prone to ischemic injury, we identified 13 transcriptionally distinct clusters at the single-cell level by examining known cellular markers [16], and we compared and visualized the different compositional proportions in each cell type identified in sham and MCAO groups in a comprehensive manner. We noted a significant decrease in the proportion of ID3 in endothelial_1 in the MCAO group. Previous studies have reported that ID3 overexpression contributed to the increased vascular neogenesis involved in blood vessels in human brain microvascular endothelial cells [64]. ID3 deficiency leads to diminished reperfusion recovery [65]. Therefore, focusing on ID3 expression in endothelial_1 may provide new ideas for the treatment of stroke. There are many types of glial cells in the central nervous system (CNS), and astrocytes play a vital role in neurodevelopment and endostasis. It can regulate neural activity, produce synaptogenic factors, control of glial cell boundary membranes and blood-brain barrier [66, 67]. These neuroendostatic mechanisms are important for the maintenance of normal CNS physiology, and abnormalities in endostasis can lead to the development of neurological disorders and, in disease states, astrocytes have functions that promote and amplify CNS pathology, including inflammation [68, 69]. The results of single cell analysis showed that SLC22A4 was mainly concentrated in astrocyte in MCAO group, suggesting that SLC22A4 may be involved in the development of neuroinflammation by affecting the endostasis of astrocyte. Therefore, the regulation of SLC22A4 expression in the astrocyte may contribute to the treatment of ischemic stroke.

Finally, we found a significant positive correlation between ID3 and Claudin5, Occludin, ZO1 in vascular endothelial cells by correlation analysis. It is known that there is a significant decrease in ID3 after stroke, and from the correlation analysis it can be deduced that there is also a significant decrease in Claudin5, Occludin and ZO1, which is consistent with our knowledge that the massive damage to tight junction proteins after stroke leads to increased permeability of the vascular endothelium, which ultimately leads to massive infiltration of inflammatory factors [70, 71].

Similarly, in astrocytes, we found a significant positive correlation between SLC22A4 and GFAP, S100β, EAAT1. Astrocytes may show increased reactivity after stroke, which may lead to an abnormal opening of gap junctions located within them, ultimately leading to increased inflammation [72]. This is consistent with our findings.

We also have to admit that there are some shortcomings in the current study. Although our study verified the expression of ID3 and SLC22A4 in animal experiments and at the single cell level, the intrinsic mechanisms were not verified in sufficient depth. More experiments are needed for in-depth exploration in the future.

## Supplementary Material

Supplementary Figure 1

Supplementary Tables 1-9

Supplementary Table 10
