# Supplementary material for: Investigating the ID3/SLC22A4 as immune-related signatures in ischemic stroke
Source: Aging (Albany NY). 2023 Dec 14;15(24):14803–29. doi: 10.18632/aging.205308 (PMC10781493; doi:10.18632/aging.205308)
Supplement: Supplementary Figure 1 [file aging-15-205308-s001.pdf]

SUPPLEMENTARY FIGURE

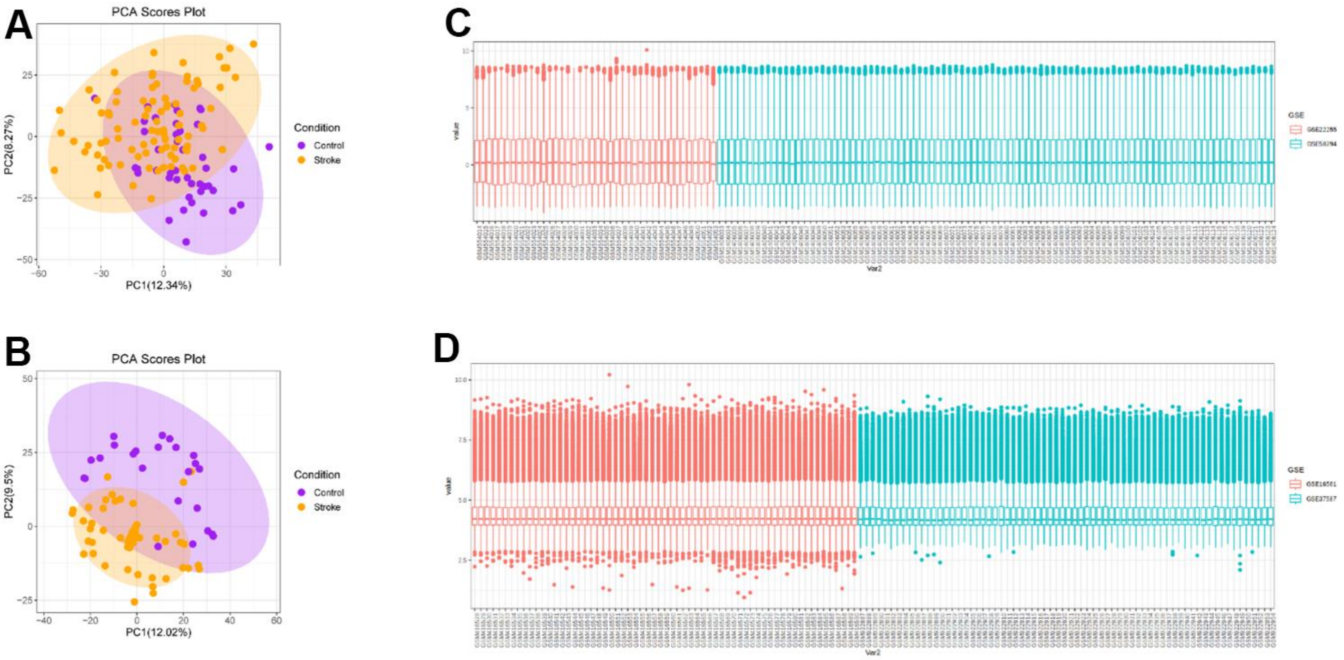

Supplementary Figure 1. Data preprocessing of DEGs. (**A**) PCA after batch correction with ComBat for GSE58294 and GSE22255. (**B**) PCA after batch correction with ComBat for GSE16561 and GSE37587. (**C**) Boxplot after batch correction with ComBat for GSE58294 and GSE22255. (**D**) Boxplot after batch correction with ComBat for GSE16561 and GSE37587.
