# Supplementary material for: Investigating the ID3/SLC22A4 as immune-related signatures in ischemic stroke
Source: Aging (Albany NY). 2023 Dec 14;15(24):14803–29. doi: 10.18632/aging.205308 (PMC10781493; doi:10.18632/aging.205308)
Supplement: Supplementary Tables 1-9 [file aging-15-205308-s002.pdf]

SUPPLEMENTARY TABLES

Supplementary Table 1. Sequences of primers.

Primer name	Sequence
ID3 FP	5’-CAGCGTGTCATAGACTACATCCT-3’
ID3 RP	5’-TCCTCTTGTCCTTGGAGATCAC-3’
SLC22A4 FP	5’-GTGTTCCCCTGTGGTGGTTT-3’
SLC22A4 RP	5’-TGCTGTTCATCTTTGCGGCT-3’
GAPDH FP	5’-GCAAGTTCAACGGCACAG-3’
GAPDH RP	5’-CGCCAGTAGACTCCACGAC-3’

Supplementary Table 2. DElncRNAs of GSE58294 and GSE22255.

ID	P-Value	FDR	log2FC	regulated
LINC00342	1.57E-11	2.22E-09	-0.695526303	Down
LINC00926	2.23E-08	8.54E-07	-0.720364083	Down
LINC00282	4.01E-08	1.42E-06	0.654800644	Up
LINC01094	1.52E-05	0.000206152	0.628948948	Up
LINC01270	8.44E-05	0.000846398	0.618386414	Up
LINC01093	0.000382075	0.002930149	0.791047092	Up

Supplementary Table 3. The Diana database found that a total of 105 potential miRNAs can bind to the above 6 lncRNAs.

Diana_res.Gene.Name	Diana_res.Mirna
LINC00342	hsa-let-7a-5p
LINC01094	hsa-let-7a-5p
LINC00342	hsa-let-7b-5p
LINC01094	hsa-let-7b-5p
LINC00342	hsa-let-7c-5p
LINC01094	hsa-let-7c-5p
LINC00342	hsa-let-7d-5p
LINC01094	hsa-let-7d-5p
LINC00342	hsa-let-7e-5p
LINC01094	hsa-let-7e-5p
LINC00342	hsa-let-7f-5p
LINC01094	hsa-let-7f-5p
LINC00342	hsa-let-7g-5p
LINC01094	hsa-let-7g-5p
LINC00342	hsa-let-7i-5p
LINC01094	hsa-let-7i-5p
LINC01094	hsa-miR-101-3p
LINC00342	hsa-miR-103a-3p
LINC00926	hsa-miR-106a-5p
LINC01094	hsa-miR-107
LINC00342	hsa-miR-10a-5p
LINC01094	hsa-miR-125a-5p
LINC00342	hsa-miR-1260b
LINC00926	hsa-miR-128-1-5p
LINC00342	hsa-miR-1285-3p
LINC00926	hsa-miR-1293
LINC01094	hsa-miR-132-3p
LINC01094	hsa-miR-135a-5p
LINC00342	hsa-miR-139-5p
LINC01094	hsa-miR-139-5p
LINC01270	hsa-miR-147b-3p
LINC01094	hsa-miR-148b-3p
LINC00342	hsa-miR-149-5p
LINC01094	hsa-miR-15b-5p
LINC01093	hsa-miR-16-5p
LINC01094	hsa-miR-16-5p
LINC00342	hsa-miR-17-5p
LINC01094	hsa-miR-17-5p
LINC01094	hsa-miR-181a-5p
LINC00342	hsa-miR-188-5p
LINC00342	hsa-miR-191-5p
LINC01093	hsa-miR-195-5p
LINC01094	hsa-miR-195-5p
LINC00342	hsa-miR-196a-5p
LINC00342	hsa-miR-197-5p
LINC00342	hsa-miR-19a-3p
LINC01094	hsa-miR-19a-3p
LINC00342	hsa-miR-19b-1-5p
LINC00342	hsa-miR-19b-3p
LINC01094	hsa-miR-19b-3p
LINC00342	hsa-miR-20b-5p
LINC01094	hsa-miR-20b-5p
LINC00342	hsa-miR-210-3p
LINC01094	hsa-miR-210-3p

LINC01270	hsa-miR-210-3p
LINC00342	hsa-miR-218-5p
LINC01094	hsa-miR-218-5p
LINC01094	hsa-miR-219a-2-3p
LINC00342	hsa-miR-221-3p
LINC00342	hsa-miR-23a-3p
LINC01094	hsa-miR-23a-3p
LINC01094	hsa-miR-23b-3p
LINC00342	hsa-miR-24-3p
LINC01094	hsa-miR-24-3p
LINC00342	hsa-miR-25-3p
LINC00342	hsa-miR-26a-5p
LINC01094	hsa-miR-26a-5p
LINC00342	hsa-miR-27a-3p
LINC00342	hsa-miR-27b-3p
LINC00342	hsa-miR-296-3p
LINC01094	hsa-miR-29a-3p
LINC01094	hsa-miR-29b-3p
LINC01094	hsa-miR-29c-3p
LINC00342	hsa-miR-30a-3p
LINC01270	hsa-miR-30a-5p
LINC00926	hsa-miR-30b-5p
LINC01094	hsa-miR-30b-5p
LINC01270	hsa-miR-30c-2-3p
LINC00926	hsa-miR-30c-5p
LINC01094	hsa-miR-30c-5p
LINC00342	hsa-miR-30e-3p
LINC01094	hsa-miR-320a-3p
LINC01094	hsa-miR-33a-5p
LINC01094	hsa-miR-33b-5p
LINC01094	hsa-miR-342-3p
LINC00342	hsa-miR-34a-5p
LINC01094	hsa-miR-34a-5p
LINC01270	hsa-miR-34a-5p
LINC00342	hsa-miR-365a-3p
LINC00342	hsa-miR-365b-3p
LINC01094	hsa-miR-423-3p
LINC01270	hsa-miR-423-5p
LINC01094	hsa-miR-432-5p
LINC01094	hsa-miR-433-3p
LINC00342	hsa-miR-449c-5p
LINC00342	hsa-miR-450b-5p
LINC01270	hsa-miR-486-3p
LINC00342	hsa-miR-589-5p
LINC01094	hsa-miR-7-5p
LINC01094	hsa-miR-874-5p
LINC00926	hsa-miR-876-3p
LINC00342	hsa-miR-92a-3p
LINC00342	hsa-miR-93-3p
LINC00342	hsa-miR-98-5p
LINC01094	hsa-miR-98-5p

Supplementary Table 4. 13 common miRNAs between validation set GSE110993 and predicted miRNAs.

ID	regulated
hsa-miR-101-3p	down
hsa-miR-17-5p	down
hsa-miR-125a-5p	up
hsa-let-7f-5p	down
hsa-miR-423-3p	down
hsa-miR-19b-3p	down
hsa-let-7i-5p	down
hsa-miR-92a-3p	down
hsa-miR-19a-3p	down
hsa-miR-16-5p	down
hsa-miR-103a-3p	down
hsa-let-7d-5p	down
hsa-miR-181a-5p	down

Supplementary Table 5. KEGG enrichment pathways of the 12 differentially expressed mRNAs.

Term	Description	Count	Q-value	Genes
Hsa04657	IL-17 signaling pathway	2	3.07×10^{-2}	PTGS2/MMP9
Hsa04668	TNF signaling pathway	2	3.25×10^{-2}	PTGS2/MMP9
Hsa05340	Primary immunodeficiency	1	6.34×10^{-3}	CD19
Hsa04662	B cell receptor signaling pathway	1	6.34×10^{-3}	CD19
Hsa04350	TGF-beta signaling pathway	1	6.34×10^{-3}	ID3

Supplementary Table 6. GO biological processes of the 12 differentially expressed mRNAs.

Term	Description	Count	Q-value	Genes
GO:0043154	Negative regulation of cysteine-type endopeptidase activity involved in apoptotic process	3	3.00×10^{-4}	THBS1/PTGS2/MMP9
GO:0043281	Regulation of cysteine-type endopeptidase activity involved in apoptotic process	3	1.26×10^{-3}	THBS1/PTGS2/MMP9
GO:2001234	Negative regulation of apoptotic signaling pathway	3	1.38×10^{-3}	THBS1/PTGS2/MMP9
GO:0150077	Regulation of neuroinflammatory response	2	1.52×10^{-3}	PTGS2/MMP9
GO:1905039	Carboxylic acid transmembrane transport	2	8.53×10^{-3}	THBS1/SLC22A4
GO:0098739	Import across plasma membrane	2	1.05×10^{-2}	THBS1/SLC22A4
GO:0002283	Neutrophil activation involved in immune response	1	1.50×10^{-2}	ANXA3
GO:0043312	Neutrophil degranulation	1	1.47×10^{-2}	ANXA3
GO:0006638	Neutral lipid metabolic process	1	3.19×10^{-2}	SLC22A4
GO:0002446	Neutrophil mediated immunity	1	1.73×10^{-2}	ANXA3
GO:0051092	Positive regulation of NF-kappaB transcription factor activity	1	3.41×10^{-2}	IL18RAP
GO:0033549	Regulation of DNA replication	1	3.35×10^{-2}	ID3
GO:0017017	B cell differentiation	1	3.38×10^{-2}	CD19

Supplementary Table 7. AUC and 95%CI for Support Vector Machines (SVM).

Gene	AUC	CI_95
PTGS2	0.686858	0.571-0.799
ID3	0.892915	0.818-0.954
ITM2C	0.837209	0.750-0.914
MMP9	0.736074	0.617-0.839
NELL2	0.742023	0.625-0.836
SLC22A4	0.891293	0.818-0.952
THBS1	0.772309	0.667-0.867
ABCA1	0.842618	0.749-0.925
ANXA3	0.871823	0.786-0.943
CD19	0.806382	0.706-0.895
IL18RAP	0.835587	0.746-0.915
NFIL3	0.813413	0.724-0.896

Supplementary Table 8. Spearman correlation between immune cell subsets and *ID3*.

Gene	Immune cells	Spearman coefficient	P-value	Type
ID3	T cells CD4 naïve	0.298475	0.000508	Positive
ID3	B cells naïve	0.294499	0.000608	Positive
ID3	Mast cells activated	0.250909	0.003709	Positive
ID3	Plasma cells	0.157024	0.072164	Positive
ID3	Mast cells resting	0.147602	0.091227	Positive
ID3	T cells CD8	0.144252	0.042889	Positive
ID3	B cells memory	0.098591	0.260719	Positive
ID3	Eosinophils	0.098383	0.261728	Positive
ID3	NK cells activated	0.049993	0.569167	Positive
ID3	T cells follicular helper	0.042843	0.325705	Positive
ID3	T cells regulatory (Tregs)	0.034368	0.695634	Positive
ID3	Macrophages M1	0.002334	0.978809	Positive
ID3	Macrophages M2	-0.024199	0.813052	Negative
ID3	Dendritic cells activated	-0.024199	0.782991	Negative
ID3	Monocytes	-0.040177	0.647386	Negative
ID3	T cells gamma delta	-0.044457	0.615742	Negative
ID3	NK cells resting	-0.081861	0.350748	Negative
ID3	Dendritic cells resting	-0.093088	0.288440	Negative
ID3	T cells CD4 memory activated	-0.145269	0.046517	Negative
ID3	Macrophages M0	-0.150459	0.044066	Negative
ID3	T cells CD4 memory resting	-0.205632	0.018010	Negative
ID3	Neutrophils	-0.272768	0.001554	Negative

Supplementary Table 9. Spearman correlation between immune cell subsets and *SLC22A4.*

Gene	Immune cells	Spearman coefficient	P-value	Type
SLC22A4	Neutrophils	0.433053	2.15E-07	Positive
SLC22A4	Monocytes	0.201701	0.647386	Positive
SLC22A4	T cells CD4 memory resting	0.187821	0.018010	Positive
SLC22A4	T cells regulatory (Tregs)	0.125462	0.151738	Positive
SLC22A4	Mast cells resting	0.147602	0.091227	Positive
SLC22A4	Macrophages M0	0.090918	0.042778	Positive
SLC22A4	Plasma cells	0.061858	0.481046	Positive
SLC22A4	Macrophages M1	0.054044	0.238245	Positive
SLC22A4	Dendritic cells resting	0.029643	0.735808	Positive
SLC22A4	B cells memory	0.029631	0.735908	Positive
SLC22A4	T cells CD4 memory activated	0.027724	0.042336	Positive
SLC22A4	NK cells resting	0.023939	0.785263	Positive
SLC22A4	Dendritic cells activated	0.013793	0.875265	Positive
SLC22A4	NK cells activated	-0.001887	0.982866	Negative
SLC22A4	T cells follicular helper	-0.035754	0.684003	Negative
SLC22A4	Macrophages M2	-0.074522	0.395746	Negative
SLC22A4	T cells CD4 naïve	-0.077318	0.022881	Negative
SLC22A4	Mast cells resting	-0.079647	0.363975	Negative
SLC22A4	T cells gamma delta	-0.089833	0.305662	Negative
SLC22A4	Eosinophils	-0.144841	0.097515	Negative
SLC22A4	Mast cells activated	-0.193911	0.025889	Negative
SLC22A4	B cells naïve	-0.250896	0.003711	Negative
SLC22A4	T cells CD8	-0.353914	3.14E-05	Negative
