# Supplementary material for: Investigating the ID3/SLC22A4 as immune-related signatures in ischemic stroke
Source: Aging (Albany NY). 2023 Dec 14;15(24):14803–29. doi: 10.18632/aging.205308 (PMC10781493; doi:10.18632/aging.205308)
Supplement: Supplementary Table 10 [file aging-15-205308-s003.docx]

Supplementary Table 10. Summary of immune-related genes extracted from the GeneCards (n=17664).

| **Gene Symbol** | | | | | | | | | | | | | | | | | | | |
| --- | --- | --- | --- | --- | --- | --- | --- | --- | --- | --- | --- | --- | --- | --- | --- | --- | --- | --- | --- |
| IKBKG | RAG2 | STAT5B | CTLA4 | RAG1 | FOXP3 | IL10 | PLCG2 | IFNG | ACP5 | RIPK1 | SYK | STAT1 | TNF | ATM | TLR2 | TNFRSF13B | CD27 | IL6 | MYD88 |
| JAK3 | JAK1 | IL2 | HLA-DRB1 | TLR4 | NFKB1 | TCIRG1 | CD4 | BCL10 | IL2RA | PDCD1 | ADA | PIK3CD | CD19 | IL4 | IL17A | TLR3 | NOD2 | CD40LG | HLA-DQB1 |
| CD8A | CD274 | MR1 | FAS | IL1B | FASLG | HLA-B | ZAP70 | TLR9 | CD40 | TLR7 | NFKBIA | IL13 | STAT3 | MBL2 | CD59 | CR2 | C3 | IL7R | IFNGR1 |
| IRF3 | CD81 | HLA-A | TGFB1 | IKBKB | CCL2 | IL2RG | NFKB2 | IL2RB | FCGR2A | PRF1 | TP53 | TYK2 | CD79A | PTPN11 | CCL5 | RIGI | HMGB1 | CIITA | TYROBP |
| HLA-G | IL7 | CD27-AS1 | CXCL8 | FCGR2B | IFIH1 | IFNA1 | TBK1 | RELA | CD86 | PTPN22 | TNFRSF13C | CD247 | TLR8 | TNFRSF1A | CCL3 | STING1 | HBA2 | HBA1 | RC3H1 |
| IRGM | WAS | CCR6 | CARD11 | HRAS | SH2D1A | NFAT5 | HLA-C | CCR5 | CD28 | CD209 | PTPRC | PRKCD | IL12RB1 | IRAK4 | C4A | STAT4 | FCGR3B | ICOS | PNP |
| IL4R | IL18 | IFNB1 | IL15 | MS4A1 | HLA-DQA1 | IL5 | CCL11 | IRF8 | CXCL10 | DOCK8 | IRF1 | ITGB2 | IL1A | FCGR3A | NEU1 | TLR1 | TNFRSF4 | HAVCR2 | HLA-DPB1 |
| ICOSLG | IL21 | BTK | CXCL12 | IL12B | TLR5 | IDO1 | ARPC1B | B2M | CLEC7A | LIG4 | KLRK1 | CD80 | CRP | PRTN3 | TNFSF13B | RYR1 | FCGR2C | CX3CR1 | TREX1 |
| IL17F | MIR155 | DCLRE1C | BRAF | MEFV | TFRC | XIAP | MALT1 | PGM3 | CFH | CSF2 | TNFAIP3 | REL | PIK3R1 | TCIM | IRF4 | AIRE | ITGAM | PIK3CG | IL12A |
| TTC7A | RFXANK | MIR146A | CD14 | ICAM1 | MAVS | MIF | CGAS | RAC2 | KRAS | UNC13D | IL17RA | CHUK | ARID1A | RFXAP | RFX5 | MPO | IRF5 | C4B | CD46 |
| IL1RN | CYBB | ACTA1 | STXBP2 | CXCR4 | C5 | FCGR1A | LRRC56 | CARD9 | CCND1 | CASP8 | TRAF3 | OTULIN | CFP | LRBA | ITGAL | MIR34A | ORAI1 | RAB27A | CD70 |
| IGLP1 | NLRP3 | EPHB4 | ALB | IL10RA | ITK | IGHE | RAPSN | IL6R | DNMT3B | WIPF1 | PMP22 | AK2 | AKT1 | TRAF6 | NSD1 | RIT1 | TBX1 | LCK | HLA-DPA1 |
| NLRP1 | NBN | TNFSF12 | STAT2 | IL1R1 | CCR1 | MIR21 | LTA | IRF7 | SOCS1 | ANGPT2 | LOC106804612 | BCL2 | SAMHD1 | MBP | IGHG1 | DHCR24 | LAG3 | TLR6 | CCL4 |
| PIK3CA | TNFSF11 | BTNL2 | PIEZO1 | FOXN1 | TBX21 | CR1 | IRAK1 | CD3D | DNAH9 | SFTPD | L1CAM | GATA3 | IGLP2 | STX11 | STIM1 | MAPK1 | ADAR | EIF2AK2 | VDR |
| CASP10 | LCIIAR | GCSIR | TICAM1 | LACC1 | IGAT | ISCW | CSF1 | SLC26A3 | NOS2 | CDC42 | IFNGR2 | RNASEL | C5AR1 | RYR3 | SLPI | TPP2 | JUN | CCR7 | LAT |
| SLAMF1 | MIR106B | SMARCAL1 | RMRP | MAGT1 | IL6ST | IL23R | CXCR3 | TNFRSF1B | IL18R1 | JAK2 | TRIM5 | STAT5A | IL9 | IL17RC | MAPK8 | MIR145 | HSPD1 | VCAM1 | THSD1 |
| NLRC4 | CLEC4D | CHD7 | KIF19 | MYC | PTEN | KIR3DL1 | MIR29A | CFI | TAP1 | NOD1 | CYBC1 | PGLYRP1 | GZMB | RIPK2 | IL22 | SELL | STAT6 | CSF3 | EGFR |
| TNFRSF18 | MIR15A | TAP2 | SPP1 | BCL6 | IL23A | HMOX1 | CTPS1 | ICAM2 | IGHM | C1QA | CXCL1 | CD69 | NCF2 | CYBA | ISG15 | CD3E | SLC11A1 | DDX41 | DNASE1 |
| TOM1 | LEP | PSMB8 | HLA-E | MSN | MYH7 | IRF2BP2 | RASGRP1 | DPP4 | BLM | MIR16-1 | CARMIL2 | BRCA1 | IL16 | P2RX7 | MIR17 | CASP1 | CD38 | ALOX5 | COPA |
| MYO5A | TRAF3IP2 | ACE2 | SERPINA3 | FOS | LY96 | CCR4 | ELANE | CAMP | PRKDC | MAPK14 | AICDA | TNFRSF14 | CD244 | LEPR | CLEC12A | CLEC4M | IFNAR2 | C1QC | CTSG |
| TSLP | LILRB1 | MIR223 | MIR150 | CCR3 | C11orf65 | TNFSF4 | LTF | ACKR2 | ADA2 | H2AC18 | KLRD1 | LBR | BST2 | CEBPB | IFNA2 | TERT | BCL11B | ITGB3 | NRAS |
| HP | TSHR | RELB | MIR140 | PSMB9 | INS | TEC | BRCA2 | NPM1 | APOBEC3G | IRGC | DOP1A | UNC93B1 | MIR126 | NCF4 | TET2 | NHEJ1 | GIMAP6 | MIR132 | SIGLEC5 |
| TRIM25 | SPINK5 | C1QB | TAPBP | NCF1 | INPP5D | DEFB4A | CD55 | CXCR1 | GIMAP5 | HCK | CD79B | MIR221 | C2 | IL37 | CD3G | TRAC | APOE | TNFRSF8 | IL33 |
| C1S | TLR10 | IL3 | SARM1 | VEGFA | ELF4 | CXCR5 | FN1 | CREBBP | FADD | CCL7 | MIR143 | KLRC1 | FYN | TRA | LYN | MIR20A | PRKCQ | S100A9 | HLA-DRB3 |
| IRF9 | IRGQ | CXCR2 | IFI16 | MIR142 | NCKAP1L | RNASEH2B | SRC | ITCH | CXCL9 | INAVA | TIRAP | CD5 | FLT4 | ACOD1 | MIR125A | ETS1 | HLA-DRA | BLNK | FCGRT |
| LBP | CD34 | MX1 | TREM1 | APC | TRIM21 | CD276 | MIR19A | IL36RN | MIR424 | CCR2 | ICAM3 | KARS1 | SKIC2 | PTPN2 | CDH1 | MAP2K1 | CEACAM3 | CD96 | GBA1 |
| CASP3 | PSMB4 | IFNAR1 | FOXC2 | IPO8 | CORO1A | MIR122 | CTNNB1 | LILRB2 | ADAM17 | BCR | CCL21 | MIR146B | PSTPIP1 | XRCC5 | ERBB2 | EPG5 | ARG1 | SOCS3 | FCER2 |
| HELLS | LCN2 | LOC107303343 | IGKC | FGFR3 | TREM2 | FCN2 | MTOR | S100A8 | CFB | CCL8 | INO80 | CALR | MASP2 | VTCN1 | PAK2 | POMC | SP1 | CFHR2 | PPIA |
| MAP3K14 | AHR | CD1A | MIR210 | CD2 | RAC1 | MTHFD1 | CCL3L1 | SASH3 | IL21R | IL27 | MIR200B | NR3C1 | BIRC3 | ITGA4 | IRAK3 | DHX58 | LYST | IGF1 | ITIH4 |
| IGH | ERVW-1 | IFNL3 | IKZF3 | C7 | SIGLEC10 | MECP2 | FCN3 | GUSB | SPN | TNFRSF17 | PRL | PTX3 | ERAP1 | MIR27A | S100B | ERCC2 | UNG | ABL1 | RNASEH2A |
| CLEC4E | RBPJ | IKZF1 | CD1D | CEACAM1 | MIR9-1 | NLRP12 | CD9 | GAPDH | THPO | DEF6 | TNFRSF9 | RHD | IKBKE | FLT3 | ERCC6 | MIR93 | MMP2 | PML | CACNA1C |
| CCR8 | CRKL | HBB | IL32 | EPRS1 | PPARG | MASP1 | OAS1 | TNFSF13 | U2AF1 | MAP3K7 | ANXA1 | MDM2 | IL4I1 | ADAMTS13 | DEFB1 | CCL18 | PTGS2 | CFTR | APOBEC3F |
| BCL2L1 | FHL1 | CD22 | EP300 | CSF3R | IL19 | SELP | DKC1 | LCP2 | NOTCH1 | LILRB4 | XRCC6 | BANF1 | TPI1 | LTBR | PLCG1 | SLAMF6 | BACH2 | CD44 | ACP1 |
| EPX | ITGB1 | ADRB2 | MIR181A1 | C8A | IGHV3-21 | MIR24-1 | HSP90AA1 | GATA2 | RNASEH2C | CD300LF | LOC100289580 | TNFSF10 | USP18 | FLG | ITGA2B | CD36 | NTRK1 | KMT2D | PARP1 |
| WDR1 | FLT3LG | TNIP1 | SLAMF7 | BTLA | BLK | TNFSF15 | MIR199A1 | NLRC5 | FCRL3 | SELE | VWF | MIR144 | CLEC6A | LGALS3 | MPZ | PYCARD | CD160 | KAT5 | MIR181A2 |
| PTPN6 | TRIM22 | CYLD | HSPG2 | NR1H4 | UNC119 | RNASE3 | NFATC2 | CCL20 | IL27RA | RORC | AIM2 | CXCL2 | C8B | SMARCA4 | SLC7A7 | F3 | BIRC2 | F2RL1 | IL12RB2 |
| RBCK1 | TGFB2 | HLA-DMA | MIR23B | HIRA | HLA-F | ENG | MIR222 | NFATC1 | BMP6 | EBI3 | LIF | BAX | LMX1B | TCF3 | SFTPA1 | SERPINA1 | LIFR | HFE | APCS |
| MMP9 | GZMM | CCL19 | MIR23A | MMP8 | TASL | COMT | MIR30E | MOG | SEMA4D | MICB | PURA | MIR92A1 | MARCHF8 | TGFBR1 | NFE2L2 | MIR29B1 | CD84 | GIMAP4 | PF4 |
| CXCL13 | DHFR | TMPO | PGLYRP3 | CXCL11 | MYOC | GPT | SRSF2 | MAPK3 | HEXIM1 | NT5E | CNR2 | VAV1 | CYCS | TNFRSF11A | CDKN1A | HLA-DRB5 | MPL | ALOX15 | C9 |
| RECQL4 | CFHR1 | CD33 | CCRL2 | POT1 | MIR124-1 | RNF31 | MIR22 | PTK2B | CCL22 | PRKCZ | MIR125B1 | NSD2 | ITGAX | FCRL4 | SH2B3 | CHEK2 | TJP1 | TIFA | CLU |
| TNFSF9 | MS4A2 | DOCK2 | CD163 | MMP1 | TAT | CD47 | AQP4 | MIR148A | ERCC1 | AR | FERMT3 | MIR31 | NEAT1 | CD83 | MUC1 | ACE | UBE2L3 | GP1BB | FCAMR |
| F8 | MIR192 | MIR30A | SKIC3 | MIRLET7D | USB1 | SBDS | CDK4 | MRE11 | PGLYRP4 | SQSTM1 | GZMA | LGALS9 | SPI1 | GP1BA | IL25 | SLC37A4 | IL9R | KIT | DEFB103B |
| CD58 | F2 | CLEC4A | S100A12 | ATG5 | SLC46A1 | PIGA | FCN1 | MIR127 | KLRC2 | IGHG2 | FGFR1 | TPO | PADI4 | TYR | ALAD | MIR205 | NOS3 | PRKCE | PLG |
| CP | IL18BP | CXCR6 | CDK9 | RANBP2 | SOD1 | CFD | CBL | UBA1 | FURIN | EDAR | ACKR4 | CEP128 | HLA-DQB2 | PRPS1 | GNLY | NKX2-5 | SETD2 | CCL17 | CD300LB |
| RNF135 | OCA2 | MAN2B1 | SLC5A6 | TMC6 | IGLL1 | CD226 | PRKG1 | IL15RA | PSMC3 | SMAD3 | ITPR3 | C1R | PLA2G7 | MIR196A1 | POU2AF1 | NLRC3 | TGFBR2 | GJC2 | ACTA2 |
| HLA-DQA2 | CEBPE | DNASE1L3 | GPI | PRDM1 | LAIR1 | CD74 | CCR9 | CAT | MIR423 | TARBP2 | HLA-DRB4 | PECAM1 | TRAF2 | VIP | CASR | HIF1A | POLE | TRAF1 | GATA1 |
| FCER1A | GPNMB | MICA | PSIP1 | KDM6A | IFI35 | HCST | MIR342 | CSK | LRRC8A | LILRB3 | G6PD | COPB1 | ZBP1 | IL11 | SOD2 | KDM4C | IL36G | CSF1R | DNMT1 |
| CIB1 | XPO1 | TOLLIP | ITPR1 | SERPINE1 | NOS1 | CCDC107 | ALPK1 | CBLB | NFKBIL1 | CTSA | POLG | SLC30A2 | IL18RAP | GRB2 | MALAT1 | SEMA3E | XRCC4 | DDX3X | FTH1 |
| DEAF1 | TRIM8 | NR1H3 | APOH | NLRP10 | MME | ALG12 | PVR | TIGIT | AKT2 | DOCK8-AS1 | CHIT1 | PRODH | SEPTIN9 | IFI27 | PIK3CB | XDH | CX3CL1 | CASP4 | CALCRL |
| TANK | NGF | UFD1 | NUP133 | LGALS1 | CDCA7 | PDCD1LG2 | IFIT1 | TBP | GBE1 | CTSC | HLA-DOA | CD24 | ABCB1 | COLQ | COLEC11 | ATRIP | AGER | CXCL5 | LY9 |
| ESR1 | SLC22A4 | SERPING1 | CRH | ZBTB24 | STUB1 | AGRN | IFNA6 | IFNA8 | IFNA13 | ALG1 | ULBP1 | CYP27B1 | UMPS | MAPK10 | APP | KLRC4 | HNRNPA2B1 | LIG1 | NSMCE3 |
| IFNA14 | PSEN1 | CD180 | EBAG9 | MLPH | BPIFB1 | GNAS | CCBE1 | PSMA7 | MIR29C | LILRA2 | GPR15 | CD48 | TSG101 | RAB7A | PRDX1 | CDKN2A | AP1S3 | MADCAM1 | IL17RE |
| PRKAR1A | NCAM1 | IFNL1 | TTN | APOB | BPIFA1 | TRNT1 | FCAR | SLC19A1 | NUP107 | MIRLET7A1 | TRIM62 | CCL26 | MUSK | RAD51 | CHI3L1 | TNFAIP8L2 | POLR3F | PEPD | FFAR2 |
| ENTPD1 | HSPA4 | KLRG1 | COL2A1 | FGA | ADORA2A | CD300LD | MIR149 | IDH1 | IFNA5 | NLRX1 | KIR2DL3 | CCL13 | TPMT | ERCC5 | RPSA | CCL24 | FPR1 | IL20 | MYLK |
| THBD | KITLG | MIRLET7I | MIRLET7B | HLA-DMB | RTEL1 | GBP5 | MSH2 | BPI | RPA1 | DMBT1 | LAMB2 | BIRC5 | HNMT | IL1RL1 | FMR1 | ERVK-6 | DEFA6 | MIR200C | TRIM14 |
| MIR338 | TRIM56 | SS3 | CD200 | SELPLG | GSTP1 | COL17A1 | TF | ALK | FPR2 | PLA2G2A | PRG2 | FCER1G | BUB1B | IL36A | TIMP1 | CREB1 | HLA-DOB | IFNL2 | DEFA5 |
| ANKRD17 | PMM2 | IFITM3 | DHX9 | CFHR3 | C3AR1 | HNRNPA1 | OTUD5 | WT1 | CLEC1A | KIR2DL1 | CISH | GZMH | RPL5 | DNASE2 | PIK3R2 | MIR224 | TNFRSF10A | CLEC10A | CD1C |
| RIPK3 | IL1RAPL2 | CYP3A4 | ITGA2 | CD8B | GPR35 | CTSB | ARID1B | GP6 | CASP9 | KRT14 | KIR2DS5 | AP3B1 | POLB | MYSM1 | HRG | TNPO3 | MUC5B | MMEL1 | SEMA4A |
| PI3 | PGLYRP2 | KIR2DL4 | IL24 | SST | RB1 | EXTL3 | SLC35C1 | TBX2 | GP2 | HSP90B1 | CLCN7 | PLA2G10 | MPV17 | WDR19 | MIR532 | AGK | TFE3 | ZNFX1 | TSC2 |
| WG | IGF1R | SFTPA2 | IFNLR1 | MIR130A | UBE2N | EPO | SDHB | SCGB1A1 | TNFRSF11B | APOA1 | EPHB2 | CHD1 | MYO9B | HAVCR1 | CD1B | CD300E | MAPK11 | SLC29A3 | FLNA |
| FAT4 | AKT3 | PMS2 | ZCCHC3 | IL31 | TTC4 | EZH2 | F9 | GSK3B | GIMAP8 | EOMES | EGF | BANK1 | FBN1 | TSC1 | HAMP | BCL3 | CAV1 | LYAR | TXK |
| PIGR | RUNX1 | HDAC1 | OPTN | CANX | UBC | MLH1 | TGFB3 | MET | CTSK | IL13RA2 | MEN1 | DSG1 | CD109 | CSF2RB | MIR30B | C1QBP | CELIAC13 | CA2 | BUB1 |
| LYZ | MIR141 | KIR2DL2 | KLRB1 | MIR486-1 | HES7 | IL31RA | DGKE | TMED1 | MIR26B | KIR2DS2 | ALG13 | MIR15B | ADIPOQ | IGAN1 | C4B_2 | CD6 | RN7SK | TRIM38 | PSMG2 |
| PCCA | VSIR | IFIT3 | FGB | SP110 | IL26 | SFTPC | IL1RAP | MCL1 | APOBEC1 | ITGAV | CTCF | PLA2G2D | ATP6V0A2 | LAGE3 | TRBV11-1 | TRBV11-3 | DHX15 | IGHV4-38-2 | CEACAM6 |
| MATR3 | BCL2L11 | FGG | SPIB | TERC | SIRT1 | MVK | TGM2 | BSG | ANXA5 | ERAP2 | LAMP2 | ASAH1 | TTR | MC2R | FGF2 | EDA | SLC15A4 | BTN3A2 | GAD2 |
| PDE4D | ADNP | TMCO1 | RPS15 | XBP1 | IL1R2 | NLRP6 | MX2 | MIR139 | PPARA | MIR185 | KRT1 | PCNA | STK4 | CLEC4G | CTAG1B | RNASET2 | MYH9 | HPS6 | BDNF |
| IQGAP1 | SERPINB3 | LZTR1 | CEBPD | FBXW7 | SEC24C | FANCA | MIR18A | SOS1 | MAP3K5 | GH1 | MSH3 | APOL1 | IGHG4 | GNRH1 | TBXAS1 | PGM1 | CBLIF | LAMP1 | SPPL2A |
| ANAPC1 | IRF2 | CD68 | DEFA1 | LY86 | IGLV5-52 | ZEB1 | CTBP1 | JAGN1 | GIMAP7 | IFNA21 | IFNA4 | IFNA7 | APRT | MIR19B1 | LTB | NFKBIB | TICAM2 | UBB | HK1 |
| NEDD4L | KIR2DS1 | SHARPIN | GHRH | CELIAC10 | CELIAC11 | CELIAC12 | CELIAC5 | CELIAC6 | CELIAC7 | CELIAC8 | CELIAC9 | TRBV11-2 | ARHGEF2 | MIR193A | MIR455 | ELN | FANCD2 | GNE | TNFSF18 |
| COL3A1 | MAL | GIMAP1 | CELIAC2 | GHR | RETN | SOCS2 | CFLAR | MRC1 | FBXL4 | CEACAM5 | CLEC5A | MIR483 | F5 | PIK3R4 | SIRPA | MST1 | SHC1 | CLEC4C | LETM1 |
| GAA | PCCB | OPRM1 | PTGDR2 | PDGFRA | TAC1 | MIR151A | MIR204 | COL1A1 | PRKCB | GTF2I | XPA | PRDM16 | IGHV3-23 | CD300A | KIR3DS1 | NLRP9 | HGF | MMACHC | COL4A5 |
| PPP3CA | RARA | CAVIN1 | IFNA16 | MIR574 | CDH23 | PTGER4 | N4BP1 | SOX2 | SELENOK | PLA2G1B | CXCL14 | MERTK | IL34 | LPL | TCF4 | MSMB | BATF | S1PR1 | IGHA1 |
| S100A7 | MRTFA | TINF2 | ABCD1 | MARCO | MIR28 | HSPA5 | PTAFR | LGALS3BP | CASZ1 | GJA1 | CAMK4 | ISG20 | MMP3 | MUC5AC | RORA | FHL2 | VIM | TRAFD1 | SERPINB1 |
| PLA2G6 | ADAM10 | NOTCH2 | SLC25A1 | ULBP3 | FLII | MIR200A | TRIM29 | FOXO3 | ENO1 | HIVEP1 | BCL11A | FANCC | HERC5 | C5AR2 | NPHS1 | MIR203A | RHOA | ALOX12 | USP7 |
| EPCAM | STAC3 | CHIA | CYP21A2 | CNTN2 | PSMB11 | MIRLET7G | PKLR | VEGFC | CCL27 | DSP | MED12 | RAI1 | BTD | TRAF5 | INDX | NUP85 | MIR20B | NFKBIE | POLR3C |
| ARVCF | IGF2R | CMKLR2 | KRT18 | CCL25 | PIBF1 | TYRO3 | CRTAM | TRAV24 | HPSE | RBP3 | IGLC3 | RAD54L | RPS19 | BECN1 | IRAK2 | SGSH | PAK1 | EXO1 | ADAMTS3 |
| NF1 | IGKV2D-29 | MAP3K8 | PIM1 | IL22RA1 | EDARADD | LILRB5 | EIF5A | CTSD | TBCE | RO60 | ATRX | SH2D1B | ZBTB16 | SMC1A | IL1RL2 | PTK2 | CHRNA1 | MPEG1 | TUBA1A |
| IGLC2 | MIR34C | NAIP | MAPK13 | CDK2 | ANPEP | UMOD | INPPL1 | CARD11-AS1 | SDC1 | MIR335 | IGHV1-46 | KDR | MIR25 | SOX9 | LY75 | HSPA8 | CMTM6 | ALDH2 | NAMPT |
| CXCL6 | IGHV3-30 | PDGFRB | LMNA | MST1R | MIR590 | PPP6C | OASL | FBXO9 | SPHK1 | TRBC2 | DNTT | ATF2 | PLA2G4A | OTC | DEFB103A | APLNR | MTHFR | PHB1 | AKIRIN2 |
| EZR | TRBV17 | FLT1 | CARD10 | RAET1E | MIR10A | SMARCA2 | ARL11 | TNFRSF25 | TRIP13 | NUP205 | C4BPA | HPS1 | RNASE7 | TYMS | SLC39A4 | SERPINC1 | CSF2RA | GSN | PVRIG |
| TACR3 | DGCR2 | POLR3E | VSTM1 | VTN | IGKV2-29 | DEFB118 | IGHV3-49 | TNFSF8 | IGHJ1 | ULBP2 | TRAT1 | EDNRB | CCL28 | MIR326 | HHLA2 | USP14 | TP63 | MIR130B | CD93 |
| IFNK | HYOU1 | EGR1 | MYH6 | SMAD4 | IL3RA | ROCK2 | CDR1 | FOXO1 | IGHV3-11 | NLRP2 | IGHA2 | G3BP1 | MANBA | TCN2 | SMPD1 | CNR1 | NMI | NFATC3 | RPS27A |
| AZU1 | USP8 | CD7 | KCNAB2 | HAX1 | CCR10 | MSMO1 | MIR330 | APOBEC3A | IGLC6 | HSPA1A | GSDMD | MIR181C | TBKBP1 | NR1H2 | INSR | IGKV2D-40 | CTSE | VSIG4 | EDNRA |
| PRSS2 | KRT16 | PTPRN | IGHV4-34 | SGPL1 | IDUA | LCP1 | CRAT | EDN1 | RET | SATB1 | MOGS | SEC61A1 | KRT10 | KRT17 | SETX | IVNS1ABP | GAD1 | CMKLR1 | ZNF341 |
| SFTPB | IGKV1-33 | IFN1@ | RNU4ATAC | MIR518A1 | NELFA | ATR | GPR183 | ARHGEF1 | IGHG3 | ATRIP-TREX1 | DLL1 | PIAS1 | IFIT5 | RNF182 | SNRPN | ANXA2 | IL11RA | RBM14 | PRIM1 |
| POLR3G | INHA | STK11 | AP2B1 | TARBP1 | DDA1 | IGHV3-15 | PARN | MAP3K1 | CLPB | CNPY3 | ALOX15B | HTN3 | KIR2DS4 | ACD | SIGLEC1 | LMO2 | PTGER2 | EIF2AK4 | EIF2AK3 |
| TMEM43 | MUC4 | WASF1 | NONO | ELK1 | NFATC4 | NBEAL2 | CXCL16 | RXRA | MGMT | SLC2A1 | SP100 | ZDHHC11 | HSPA1B | HSPA9 | TNFRSF10B | PSPC1 | WDR4 | MIR107 | HDAC2 |
| PQBP1 | PRKCA | ABCA1 | TNFRSF21 | LOC106694316 | OAS2 | EEF1A1 | HVCN1 | CD207 | FSTL1 | NPC2 | SLC39A8 | BUB3 | IRS1 | IRF6 | PRKACA | RERE | AGTR1 | TXNRD2 | GFI1 |
| RNF125 | DLK1 | PRKD1 | ATG16L1 | DEFA3 | SKP2 | LAIR2 | TRADD | DCT | HDAC9 | METTL3 | IGHV3-33 | SFPQ | CDK1 | JAZF1 | ZC3HAV1 | CTSL | SF3B1 | THBS1 | C8G |
| DCTN4 | FCHO1 | ACTN4 | SLC17A5 | PIGG | HPS5 | RASA1 | CCND3 | HDAC4 | IGHV1-69 | IGHV1-2 | IGHV3-13 | IGHV4-59 | IGHV2-70 | PTH | CD177 | C4BPB | F11R | GBP1 | ITGAE |
| TIMD4 | IL17D | OTUB1 | JMJD1C | IGHV3-53 | IGHV2-5 | IL13RA1 | KLK4 | STAR | TRGV3 | RFX1 | KCNN4 | ENTREP2 | LOC106694315 | LPRS | ATPLS | CLLS1 | CLLS2 | IFI30 | RAP1A |
| CLLS5 | IGHV3-48 | IGHV3-7 | IGHV4-39 | TGIF1 | CYP7B1 | SECTM1 | PGF | HMGA2 | PKM | SNAP25 | CD200R1 | IFI44 | KIR2DL5A | TAB3 | UBE2V1 | IL12A-AS1 | PLEC | CLLS3 | CLLS4 |
| ATN1 | LPO | SIGLEC7 | RARRES2 | GP9 | CSN1S1 | POLR3B | PPP3CB | XRCC1 | MAF | UBP1 | PILRA | GDNF | DGCR8 | GGH | MIR32 | BTN3A1 | TMEM165 | NCK1 | DDB1 |
| IGLC1 | ORM1 | FOXP2 | CDSN | WFS1 | DOK7 | IGHV1-24 | KPNA2 | DHX36 | TECPR2 | AGPAT2 | WRN | DGUOK | PSME3 | PRKN | TRBC1 | SP140 | APAF1 | MARCHF2 | TRGV9 |
| IGHV1-69D | ALDOA | OCLN | CHRNG | MAP2K2 | RIPPLY2 | RPS6KB1 | GNB1 | HNF1A | FSCN1 | SSB | ENDOD1 | TRGV5 | IGHV4-31 | ALG6 | G6PC3 | SOS2 | NRP1 | DIAPH1 | IGHV2-70D |
| E2F1 | IGHV1-69-2 | IGHV3-64D | YWHAZ | LILRA5 | SH2D2A | TRGC2 | PAX1 | FGL1 | IGHV7-81 | IGHV4-28 | IGHV1-18 | IGHV1-3 | IGHV3-20 | IGHV3-35 | IGHV3-38 | IGHV3-43 | IGHV3-66 | SDHD | SNAP29 |
| IGHV3-74 | KIR3DL2 | SEPSECS | GPR108 | IGHV3-16 | IGHV3-64 | IGHV1-58 | IGHV2-26 | IGHV6-1 | IGHV1-45 | IGKV2D-24 | IGHV4-30-2 | IGHV7-4-1 | IGHV5-10-1 | LTA4H | KNSTRN | TRGV4 | TRGV2 | IGHV3-72 | IGHV3-73 |
| ATP1A2 | IGHV4-4 | IGHV4-61 | IGHV5-51 | IDH2 | IGKV2D-28 | FCGR1BP | IFNE | LRRC32 | SMARCB1 | LUZP1 | PAX5 | ELP1 | ATRN | BAK1 | IGKV2-40 | HPRT1 | MGAT2 | TRGV8 | KRT5 |
| DGCR6 | PRKD2 | APOBEC3B | COL5A1 | SLC35A2 | CEP57 | NEDD4 | JUNB | KMT2E | IGKV1-5 | TIA1 | HNF1B | PSMC4 | CALCA | ADGRE3 | CBS | GATA6 | POMP | PDPK1 | CANDF1 |
| ABCB4 | NCR2 | MS3 | DEFA4 | IGLV6-57 | IGKV4-1 | IGLV1-51 | IGKV1D-16 | IGKV3-11 | IGLV1-40 | IGLV2-14 | IGLV3-19 | IGLV3-25 | IGLV7-43 | IGLV1-44 | IGLV2-8 | IGLV4-3 | IGKV1-17 | YY1 | RNF168 |
| IGLV8-61 | TRPC6 | PELI3 | IGLV3-21 | IGLV2-11 | IGLV3-10 | IGLV3-27 | IGLV7-46 | IGKV1-12 | IGKV1-16 | IGKV1-27 | IGKV1-39 | IGKV1D-13 | IGKV1D-17 | IGKV1D-8 | IGKV2-30 | IGKV3-15 | IGKV3D-15 | IGKV5-2 | IGLV1-47 |
| IGLV3-1 | IGKV1D-33 | IGKV2-24 | IGLV1-36 | IGKV1-9 | IGKV1D-12 | IGKV1D-43 | IGKV2-28 | IGKV2D-30 | IGKV3-7 | IGKV3D-11 | IGKV3D-20 | IGLV10-54 | IGLV2-23 | IGLV3-12 | IGLV3-22 | IGLV4-60 | IGLV5-37 | IGKV3D-7 | IGLV2-18 |
| IGKV6D-21 | IGLV3-16 | IGLV3-9 | IGKV1-6 | IGKV2D-26 | IGKV6D-41 | IGLV1-50 | IGLV9-49 | IGKV1-37 | IGKV1D-42 | IGLV11-55 | IGLV5-48 | IGLV2-33 | IGLV3-32 | SAG | NCSTN | LILRA3 | SLA2 | IGKV1-8 | IGKV6-21 |
| PTGS1 | IGLV4-69 | IGLV5-45 | IGKV1D-39 | IRS2 | MAP2K4 | TRBJ1-1 | CD52 | LTB4R | PGR | CEP126 | TAB1 | UBASH3A | MYCN | RBP4 | STX4 | TNIP2 | MB | FBXW11 | GLB1 |
| DICER1 | SCARB2 | LDHA | CNTF | RNASE2 | ORAI2 | FEN1 | VCP | MCM4 | TFAP2A | IFNA10 | SERPINB2 | PILRB | LILRA1 | GYPC | TET1 | LIG3 | GPR65 | PIK3R3 | IFITM2 |
| NPY | CRLF2 | PXK | CCN2 | SMAD7 | ELMO1 | F13A1 | IGHD1-1 | LMNB1 | CHAT | TIMP2 | TRBD1 | TRBJ1-2 | DUOX2 | PIK3R5 | NUP93 | BTRC | TBC1D23 | ETV6 | MYLIP |
| CDC73 | SRF | NECTIN1 | NFAM1 | TRBJ2-3 | LGR4 | BTN3A3 | MPIG6B | CTTN | MAPK12 | NCR3 | PARP9 | GGT1 | AARS1 | APOBEC3C | MED20 | TRBJ1-4 | TRBJ2-1 | BAD | YES1 |
| TRBJ1-5 | GSS | ERBB3 | PTMA | TNFRSF12A | OLR1 | ADGRE5 | MEX3C | SCARB1 | DCD | TRGC1 | IGLC7 | RTN4 | TLN1 | SLAMF9 | ACHE | CDH2 | ATXN2 | TRBJ2-7 | TRBJ1-3 |
| BRIP1 | TRBJ1-6 | IGKJ1 | PLA2R1 | BMP2 | NECTIN2 | CCNA2 | MT-ND1 | MYO1E | DSC1 | SLC3A2 | IGLJ1 | IGKV1-13 | IGKV1D-37 | SPEN | TXN | AFP | SPATA5 | PLCE1 | LAMP3 |
| SHH | SPTAN1 | MSH6 | OSCAR | UBAC2 | RUNX2 | TRIM26 | ARHGAP45 | ROCK1 | RUNX3 | CLECL1P | CALCOCO2 | TNFRSF6B | ADORA2B | GAB2 | HSPB1 | PSAP | SLC25A13 | PIANP | RRM2B |
| CDKN3 | RAD50 | OAS3 | SH3KBP1 | VPS45 | CCND2 | MMP14 | MORC3 | CBFB | TP53BP1 | ZFHX3 | ITGA3 | TRIM13 | KLHDC8B | NGFR | IGHD | ARSB | CLEC1B | HACE1 | PIK3C2A |
| MBD4 | TRIM31 | GRN | RNF8 | DUSP1 | CLCA1 | LARP1 | FGFR2 | RELN | ODC1 | PLXNC1 | SIGLEC15 | RGS1 | JUP | NPC1 | MUC16 | PRSS57 | PLAU | MMP12 | RAET1G |
| NUP210 | TDO2 | GNAI2 | RHCE | TMPRSS2 | PHB2 | PLA2G5 | AP4E1 | MIR99A | RARB | TGM1 | PLK1 | RALA | GALE | MDC1 | XCL1 | LGI3 | CDKN2B | FZD4 | WNT2B |
| SERPINB9 | SAA1 | MOV10 | HPS3 | POU2F2 | CPLX1 | USP15 | PDIA3 | GNPTAB | SEC23B | CHRNE | CRHR1 | NPHS2 | TP73 | PSMD12 | REG3G | ALOX12B | COL1A2 | TREML1 | TRIM37 |
| WDR73 | IGSF6 | RIOK3 | BCS1L | SLX4 | ZMYND11 | ITPR2 | OTUD7B | ATG7 | TRAF3IP3 | HGSNAT | CMA1 | MYO5B | CTSS | BMAL1 | TRIM24 | RPS14 | ACKR1 | LFNG | DLL3 |
| ALPL | MESP2 | IGHV8-51-1 | MAG | NAGLU | AOC1 | UBE4B | TRBJ2-2 | TRBJ2-4 | TRBJ2-6 | TRBJ2-5 | CHGA | PRSS3 | SLA | CADM1 | LAMC2 | NUP160 | TEK | DAAM2 | YAP1 |
| KCNJ11 | YTHDF2 | LEF1 | AIP | GNS | LRRK2 | ATP6V1B2 | ANKRD26 | CD1E | REV3L | KNG1 | TAGAP | CHRNA7 | SIGLEC9 | MYO1G | JUND | GNB2 | DLAT | GBP2 | MSH5 |
| SIGIRR | FANCL | TRIM6 | SLC9A3 | ZFAT | SLC39A14 | ERCC4 | MYO18A | FYB1 | TRAIP | VAMP1 | DBR1 | TNFAIP6 | ZFP36 | ALOXE3 | TSPO | AZI2 | FOXC2-AS1 | EPPIN | LAMTOR2 |
| DEFB119 | SLC15A3 | FERMT1 | ACTR3 | GIMAP3P | PSMA3 | LST1 | POLR2A | GFAP | SYT2 | PDE4A | KLK6 | ACVRL1 | ECSIT | ERCC3 | HARS1 | CST7 | ESS2 | MS2 | MS4 |
| IFNG-AS1 | HADHA | RAD52 | LRP1 | PTPN3 | TALDO1 | UBE2D1 | SOX11 | ACTR2 | SDHA | COL4A3 | RAET1L | MRPL28 | FGL2 | CD2AP | ATP8B1 | KLRC4-KLRK1 | CD63 | TRPM2 | CLDN1 |
| BAG6 | DSG3 | CPT2 | DEFB4B | ITGA5 | TERF1 | MCOLN2 | NDP | OSGEP | KIF11 | NSUN2 | IFI6 | PDGFB | TRBV12-3 | TJP2 | GDF15 | GFPT1 | MCOLN1 | HSD11B1 | MT-CO1 |
| UBE2D3 | NUP214 | CPLANE1 | MIR152 | MYO9A | SPPL2B | PIKFYVE | FTSJ3 | GALC | DEFB124 | ATP6AP2 | PHF11 | GPR84 | WRAP53 | HSP90AB1 | MYB | TP53RK | H2AX | BCL2A1 | SERINC5 |
| AHCY | HPR | DCSTAMP | SLC5A7 | RPL11 | TKT | VCL | KCNMA1 | OSMR | TRAV27 | CD300C | MEF2C | MITF | LIPA | NOX4 | CARD14 | TRIM41 | VHL | VAMP7 | WNT5A |
| LDLR | SEMA3A | POU2F1 | BTNL8 | ADCY7 | TRIM35 | ATF3 | CDC25C | CHRM3 | SLC4A1 | ABHD16A | RPS26 | BST1 | SAMD9L | ENPP1 | FCRL2 | TOMM70 | LAMA5 | HMGB3 | FOXP1 |
| FLNB | MIR197 | WRNIP1 | C17orf99 | HUWE1 | ORM2 | PDHA1 | CDCP1 | CDKN1C | CRYAB | FABP5 | TRGV1 | TRGV10 | TRGV11 | TRGJ1 | RNPC3 | PKP1 | ILF2 | XCL2 | GPX1 |
| TCOF1 | KMT2C | DEFB127 | PRLR | VIPR1 | IQSEC2 | TPRKB | SMAD2 | SCGB3A2 | MLANA | BGLAP | MAP2K3 | CFHR4 | MUC2 | GBP7 | FGF8 | PIP | TAFAZZIN | IL36B | GLA |
| IL17B | FANCM | ATG12 | PSME2 | DEFB126 | IL22RA2 | KRT8 | PLAT | CASP6 | FUCA1 | CDC20 | DDX6 | TWIST1 | FGF7 | GARS1 | MAP2K6 | RASGRP2 | KIR2DS3 | JCHAIN | DST |
| TRBV12-4 | PSMD5 | TRIP11 | NOP10 | MAOA | ITGA6 | ACR | TRBV10-1 | TRBV10-2 | TRBV10-3 | GNAQ | PSMD4 | CRLF1 | DYRK1A | ASXL1 | LAMA3 | CALM1 | INF2 | IGAN2 | OPRD1 |
| ANKRD55 | TRIM4 | ITLN1 | ABCC4 | IDO2 | PREP | TGFA | APOBEC3H | PCBP2 | PROS1 | GRHL3 | BMX | COLEC12 | ECM1 | SPIRE1 | FCRL5 | CCK | SKI | IGES | DHCR7 |
| FZD6 | EHMT1 | DYNC1H1 | ITGB7 | PRKCG | ITGB4 | GPER1 | PI4KA | PAG1 | RPS24 | MID1 | ARPC2 | HOXB13 | HM13 | MAPK7 | NPSR1 | TMEM33 | CXCL3 | PACS1 | CD151 |
| SIGLEC12 | SLC7A5 | NHP2 | SCNN1A | GPR182 | ATP1B1 | GAST | TRIM40 | TACR1 | PLD2 | PPP3R1 | CGA | MXI1 | MUL1 | FTL | COG6 | CCL23 | HUNK | NUP98 | BMP4 |
| FCMR | NR4A1 | HBG2 | UBE3A | CABIN1 | OPRL1 | GHRL | GLS | PPP1CA | GAL | SOAT1 | ADAM33 | DMD | ATP7A | POLR3D | NANOG | PROCR | IGBP1 | PRDX5 | TRBV19 |
| FGF23 | SLAMF8 | KLK3 | SUOX | DES | TFR2 | GALNS | VPREB1 | BLOC1S5 | ABCB11 | JAM2 | UBE4A | RBBP8 | TRIM28 | ARNT | ADAM8 | DIP2C | LALBA | SIAE | AP1B1 |
| SMARCA1 | GSR | BGN | SOD3 | COTL1 | SMC3 | CYP19A1 | MIRLET7C | BMI1 | ATP11A | NNT | ESR2 | MDK | HRH4 | JAM3 | PELI1 | MAPKAPK2 | HSPA6 | GSTM1 | SRD5A2 |
| ADH5 | CSNK2A1 | CYSLTR1 | SALL4 | COL7A1 | UBD | ARRB1 | PSMB5 | DNM2 | CLDN11 | PHEX | ALG9 | SPTA1 | PCGF2 | FUT2 | GABRD | MMP23B | MAPT | CENPB | CUL5 |
| HMGB2 | TGM3 | SWAP70 | ACADVL | H2BC21 | HTN1 | TRIM32 | VAV3 | ABCE1 | CYP2E1 | PTPRO | DCAF8 | PTPN1 | TXNIP | CTNNA1 | PLPP6 | CANDN1 | ATOD3 | PRKAA1 | GARIN5A |
| FECH | EFTUD2 | PICALM | ABCG2 | CYP2D6 | BCKDHA | LPP | CHEK1 | AZGP1 | GSTM3 | TRIM27 | HDC | ELOC | MECOM | AMACR | SIGLEC11 | LAMA4 | POLK | RPS6KA3 | DCC |
| IGFBP2 | PSMF1 | RPS17 | CSTA | TRDMT1 | CEBPA | HBZ | CUL1 | DRD2 | SLC15A2 | APBB1IP | DTNBP1 | KIF5B | LMNB2 | SCN5A | APOBEC3D | PLA2G2F | XPC | PSMB2 | NQO1 |
| GALNT14 | PTHLH | UHRF1 | IL20RA | UBA7 | IL17RB | FBXO38 | B4GALT1 | TKFC | CLDN2 | MAGEA3 | DUOX1 | LIMK1 | PSMA5 | FYCO1 | TRAF4 | CASP7 | KPNA3 | MNDA | CTNND1 |
| KDM5C | EXOSC9 | HMOX2 | CYP1A1 | WNT10A | BLOC1S6 | SLEB3 | ATOD1 | ATOD5 | ATOD6 | ATOD7 | ATOD8 | ATOD9 | SLEB12 | SLEB13 | SLEB14 | SLEB15 | SLEB4 | ATP6AP1 | ASPH |
| SLEB8 | FUT4 | EIF4G1 | CYP11A1 | ATP7B | UQCRFS1 | DYNLL1 | GATA4 | PGRMC1 | CCL16 | CLN3 | TCL1A | NCR3LG1 | SPA17 | CDH5 | TBXT | ATAD3A | MRAP | SLEB5 | SLEB7 |
| P4HA2 | UBE2W | ZFPM1 | TAL1 | CLEC2D | LAT2 | CST3 | ENPP2 | PSMC1 | ARF1 | TIMP3 | MFGE8 | RAD51D | CD8B2 | DLL4 | FGFR4 | DCAF1 | MAN2A1 | MMUT | NUMA1 |
| P4HB | CALM3 | PTPRJ | LMOD1 | CD72 | VEGFD | RAD54B | ST14 | SSR4 | POLR1C | GJB6 | BAZ1B | PIGT | ACAT1 | MTAP | SERPINB4 | NUDCD1 | FFAR3 | IGL | H19 |
| KPNA4 | CCNB1 | APPL1 | CMTM4 | RAD51C | COL6A3 | BRD4 | EPB42 | ACKR3 | MAPKBP1 | PSMA1 | CLTCL1 | CORT | LHCGR | TRIB3 | PON1 | TNFSF12-TNFSF13 | ZFP36L1 | ALG3 | DBNL |
| H2AC20 | ARPC4 | SOX18 | RNF19B | THRB | BCL6B | FUT8 | CLEC2B | P2RY14 | TUSC3 | TRDC | VPS4A | CDC25A | TAX1BP1 | TRAM1 | RXRB | VNN1 | IBD5 | TCP1 | SPRED2 |
| TFF3 | TERF2 | IGLL5 | ERN1 | GJB2 | RNF34 | RNF114 | ALOX5AP | DTX4 | RPLP0 | KLF6 | HMGCR | LTBP1 | MIR331 | CCDC39 | PMP2 | BATF2 | ANO5 | ESCO2 | CALM2 |
| P2RX4 | UBE2D2 | FRAXA | ZBTB12 | SNRNP70 | TRAV18 | TRAV19 | TRAV38-2DV8 | TRAV8-4 | TRAV9-2 | TRAV14DV4 | TRAV2 | TRAV38-1 | TRAV40 | TRAV9-1 | TAPBPL | PLD1 | C1orf105 | GSDME | DPEP1 |
| FIP1L1 | MCM3AP | PSMC6 | CEP290 | GCK | TRIM63 | SKAP2 | ADAMDEC1 | MEG3 | NOX1 | NIPAL4 | TUBB | ACP3 | SLC1A5 | POLR3K | POLR3H | LRP5 | NRTN | MAP3K3 | CYP11B1 |
| BSCL2 | WASL | RAPGEF3 | MYL9 | PAH | FLVCR1 | REG3A | PTPN13 | IGKV3-20 | IMPDH2 | SPAG1 | CD37 | MC1R | PTPN4 | PLD3 | BCHE | TYRP1 | PROC | NTRK2 | RAE1 |
| IGK | CTSH | SOX4 | SH2D3C | STX1A | NINJ1 | PSMA6 | REN | HEXB | NMB | TRAV12-2 | CENPF | SBNO2 | SPTLC2 | SEC22B | APOA2 | PSME1 | ALMS1 | RHBDF2 | RHOH |
| PSMB7 | P2RX5 | POLH | POLD1 | TRBV7-9 | CDK6 | TUBA8 | SPNS2 | MSX1 | FER | KDM1A | MFHAS1 | AOAH | PROP1 | TRPC1 | NF2 | PSG1 | ARIH1 | CCL15 | MKI67 |
| TRAV22 | HCRT | IL20RB | MPDU1 | OCRL | EPHA2 | RPA2 | DDX17 | ATF1 | MIR155HG | SUV39H1 | TRAV21 | ASS1 | MAP2K7 | LIN28B | MIR134 | EIF2S1 | DAB2IP | TXLNA | VRK3 |
| CEACAM8 | TFG | ABCC1 | TRAV29DV5 | PSMD1 | NARS1 | LZTFL1 | ADM | PSMD2 | SPON2 | SRGAP1 | MUS81 | COL5A2 | DCK | DDX60L | VAMP8 | LPIN2 | EYA4 | ZIC3 | PPP2R3C |
| PTGDR | STEAP3 | COL4A4 | VAV2 | TRAJ3 | AXIN1 | KEAP1 | EPHA3 | ZDHHC5 | PANX1 | SLC35A1 | ARSA | SKP1 | CHD4 | CAST | GC | CAND1 | TRBV6-5 | SLC26A4 | SIGLEC8 |
| LOC107133510 | KLK7 | ZNF683 | TRAV8-6 | ARHGDIA | ITPKB | MIR375 | GRP | RBX1 | CAMK2B | PSMD3 | FARSA | TRIL | CAMK2G | PSMB3 | TRAV17 | TOP3B | PSMC5 | APEX1 | LOC106099062 |
| DIABLO | MCM10 | PTS | ORMDL3 | PKD1 | TRBV16 | WASF2 | CFL1 | FANCI | PCSK9 | TRAV12-3 | NPEPPS | TRIM68 | EOGT | CCL14 | PPP1R12A | CBX5 | IGFBP1 | FGF10 | RNF115 |
| MYO1C | FES | DEFB104A | PDCD4 | GAS6 | SMARCA5 | RPE65 | CDKN2C | DPM2 | PIK3C3 | YWHAB | TRAV13-1 | TRAV13-2 | TRAV20 | TRAV23DV6 | TRAV25 | TRAV39 | TRAV41 | RICTOR | UBE2I |
| TRAV16 | CNN2 | CLC | PLEK | TRAV10 | TRAV12-1 | TRAV26-1 | TRAV26-2 | TRAV3 | TRAV36DV7 | TRAV4 | TRAV8-2 | TRAV34 | TRAV5 | TRAV7 | TRAV8-1 | TRAJ31 | TRAJ42 | TRAV6 | TRAV8-3 |
| IGHV3-9 | TRAV30 | TRAV1-1 | TRAV1-2 | DUSP4 | TRBV3-1 | DYSF | NKX2-1 | SIN3A | GP5 | MYO1B | UBE3B | CYSLTR2 | GALK1 | GRAP2 | PGBD3 | B3GAT1 | PRKRA | PPL | GNA11 |
| PTCH1 | MTRR | FOXG1 | GPC3 | LACTB | F10 | UFL1 | CTSV | KPNB1 | PRDX6 | CSNK2B | DISC1 | FCRL1 | KLLN | SNRNP200 | IMMT | ASGR2 | PRG4 | PRKAA2 | TRPS1 |
| ALCAM | DNASE1L1 | RDX | SERAC1 | KLK2 | DLG1 | CD53 | COMMD3 | PSME4 | TRIM10 | GPR31 | DEFB131A | CASP5 | MYH10 | NLRP4 | RFT1 | IFNL4 | ANGPT1 | FCRL6 | TRPC3 |
| MEST | TRBV18 | CNP | TREML4 | PLCB1 | MARCHF1 | CXADR | SLC7A9 | LMAN1 | IFI44L | FOLH1 | CCDC86 | UBR4 | INSL3 | IARS1 | GGCT | TRBV4-1 | TRBV4-2 | AKAP12 | BAG3 |
| BRI3 | TRBV20-1 | PSMB1 | PLCD1 | AASS | COPS5 | CLCNKB | CYFIP1 | KDM6B | HR | ODAD3 | PSMA2 | CCDC47 | HTT | SNRPD1 | PTPN12 | STIM2 | TRBV28 | TRBV6-3 | RBM8A |
| TRBV2 | APPL2 | YWHAH | ENPP3 | TRBV5-6 | TRBV6-1 | TRBV5-1 | TRBV7-3 | TRBV9 | TRBV23-1 | TRBV6-6 | TRBV24-1 | TRBV5-3 | TRBV5-5 | TRBV6-8 | TRBV7-1 | TRBV7-6 | TRBV13 | TRBV30 | TRBV5-4 |
| TRBV6-7 | TRBV27 | TRBV25-1 | TRBV29-1 | TRBV12-5 | TRBV14 | LMBRD1 | HYDIN | AGL | TPSAB1 | ARID2 | NBR1 | FBXO32 | BMP7 | ATP2A2 | TBX20 | LYVE1 | CISD2 | TRBV5-7 | TRBV6-4 |
| NUP37 | TRBV7-2 | TRBV7-4 | TRBV7-7 | SLC39A7 | HLA-DPB2 | TNFRSF10D | VAMP3 | ICAM5 | PFN1 | TRPV4 | PLCB2 | ID1 | MUC6 | ABCG8 | PSMD14 | TNXB | BUD23 | ATP6V0A1 | NCL |
| FANCF | H3C1 | PLA2G4C | DSG2 | PSMB6 | CELA2A | GZMK | FADS1 | SCIMP | THY1 | CUBN | PKHD1 | PLVAP | COL10A1 | CLDN12 | RAB7B | NEFL | TIMMDC1 | GAS5 | FANCG |
| ITGAD | FLG2 | COG7 | WFDC2 | IGLV5-39 | CRCP | DCST1 | CARM1 | ICAM4 | KMT5B | DIS3 | DHPS | AP3D1 | CD5L | RAB10 | CAPN1 | MIR184 | RAN | PDAP1 | AP2S1 |
| SLC7A11 | PSMD13 | ABCC8 | ILF3 | F2R | FAP | PRNP | DNAAF2 | CUL4B | PRKD3 | PYGL | DAXX | BCOR | PSMD7 | FOXJ1 | TRDV1 | TRDV3 | PRPF19 | TRDV2 | PSMC2 |
| IKBIP | HLA-H | AAAS | SLC3A1 | CD300H | APTX | MLXIPL | GOT2 | ABCA3 | TRIM48 | ABCG1 | SLC11A2 | XK | DNAL1 | CTNNBL1 | MCM2 | COG4 | PXN | FBXO10 | ANG |
| LAMA2 | BATF3 | NUP155 | ELAVL1 | PSMA4 | KIF15 | SIK2 | BAZ1A | PAX6 | TCF7 | KRT19 | PSMD6 | PSMD11 | HSD17B4 | COQ8B | HLA-DQB3 | SAMD9 | CTC1 | TMEM50B | ABCC2 |
| GPX3 | IVL | TTI2 | NR4A2 | SLURP1 | PRRC2A | PLCB3 | SDCBP | HYAL1 | MUC3A | XRCC3 | PPP2CA | BAP1 | GAPT | PAF1 | SCNN1B | AGBL5 | SMPDL3B | SUCLG1 | MCCC2 |
| RHOG | TRIM7 | LEXM | CLDN3 | CNBP | MIR148B | RPS10 | RB1CC1 | RPS29 | SATB2 | POSTN | PSCA | NTF3 | MMAB | SLC2A5 | ADGRE1 | SH3GL1 | LTN1 | JAG1 | MAP3K4 |
| CYP2C19 | UBE2J1 | KRT9 | SLFN11 | EPB41 | CACTIN | RPL35A | ELF1 | MAD1L1 | DEL22Q11.2 | TRAV35 | PCSK1 | ST3GAL1 | PLOD1 | ACAA1 | HLCS | DEFB131B | AREG | SLC4A2 | EIF4E |
| FER1L5 | MACROH2A1 | AIF1 | NFKBIZ | GJA5 | DDX60 | HLTF | ACTC1 | MYDGF | COL11A2 | IPO7 | TCF12 | GADD45A | ANTXR2 | CRY2 | SPHK2 | PRSS1 | SCN9A | SERPINH1 | FLI1 |
| AGTR2 | PDE7A | SUZ12 | NMBR | YWHAQ | CAMK2D | CYB5R3 | ATP6V0D1 | HECTD3 | LCAT | PLEKHM1 | CYP24A1 | MARVELD2 | YWHAE | HRH1 | TARS1 | CLDN19 | NDUFS4 | TPM2 | PEX19 |
| UNC45A | PPP2R1B | AKR1A1 | SELENON | SIRT6 | PMVK | ALDH18A1 | SLC2A3 | NUS1 | ACTG1 | ROR2 | TREML2 | ELOB | THEMIS | RUBCN | TRBV4-3 | ORAI3 | OXA1L | SLC6A20 | ST6GAL1 |
| ZBTB20 | ECE1 | RPS27 | NDFIP1 | PTH1R | LRP4 | IGHV3-43D | IGHV3-38-3 | OMP | TRPM4 | NR2C2 | CYP1A2 | PON2 | PRDX3 | PRKCH | DDIT3 | NCOR2 | TCF7L2 | NGLY1 | VAMP2 |
| PPP3CC | SUN2 | GPBAR1 | DEFB130B | BPGM | ADORA3 | AGA | H3-7 | PRDX4 | SUGT1 | CCT8 | TRIP4 | CCNO | TRBV6-9 | TRBV7-8 | TRBV5-8 | MPI | CNTNAP1 | AIMP1 | SEC14L1 |
| DIPK1A | SERPINA6 | NDUFC2 | DBN1 | PCAP | PSORS5 | LOC109504725 | PSORS4 | PSORS6 | HPC3 | LOC110806306 | PSORS10 | PSORS3 | PSORS7 | PSORS8 | PSORS9 | AA1 | HPC10 | GLUL | ELAC2 |
| HPC4 | MIR449A | MIR521-1 | ZFHX3-AS1 | PSORS11 | TUBB2A | SRP54 | IGHV4-30-4 | IGHV1-38-4 | PPP1CB | FTCD | PRMT5 | UROS | CLOCK | DUT | SELENOP | F11 | FAH | HPC14 | HPC15 |
| PADI2 | HPC5 | HPC6 | HPC7 | BRWD1 | ZEB2 | GBP3 | CCL3L3 | NUP88 | MCPH1 | SDCCAG8 | ULK1 | TLL1 | LGMN | CLTA | DHX8 | PVT1 | SURF4 | RACGAP1 | ART1 |
| MT2A | APOA4 | ARHGAP31 | RFC2 | DDR1 | IGHV3-30-3 | IGHV3-30-5 | STAG2 | RECQL | SLC2A4 | CKB | PSENEN | RCAN1 | DDX1 | NR2F2 | AURKA | RTN4R | HAND2 | YWHAG | TUBB4B |
| SLC25A15 | CACNA1H | SNAP23 | SLC40A1 | JPH4 | OLFM4 | OSTM1 | DGKA | ANKFY1 | TUBA1B | DDC | UMODL1 | NUP62 | KLKB1 | IGKV@ | UBA52 | PRKAB1 | PTGER3 | CAPZA2 | CDR2 |
| MAGEA1 | RECK | FAM111B | PTPRZ1 | TUBB3 | SIGLEC14 | IGHV1-8 | SAFB2 | YDJC | GOLM1 | MMP13 | DYNC1LI1 | SYNPO | RASGRP3 | KLRC3 | GRIN2A | TK2 | CASP2 | ATP6V1H | TGFBR3 |
| SCN4A | NUP153 | LSP1 | RNF5 | STMN1 | COMMD9 | NMU | GCG | ADSL | PXDN | TUBGCP2 | SM1 | COMP | NCOR1 | RPS20 | DKK1 | LOX | CBX3 | H1-0 | ATP6V1A |
| VPS33A | ADAP1 | DNAJC3 | KCTD1 | PLA2G3 | SLC19A2 | PAWR | AMFR | PSMD9 | SHMT2 | NDN | PKD2 | PDSS2 | CLEC9A | ARHGAP26 | GABARAPL2 | TIMM8A | HJV | SOX10 | ARRB2 |
| GALT | TAC4 | HLA-S | UBE2B | SPEF2 | WWOX | SEMA6D | RELT | ALDH3A2 | KLK1 | WASF5P | DCN | HCP5 | DNM3 | MRC2 | DRD1 | MUC13 | PACRG | PANK2 | ERBB4 |
| CLTC | ARMC5 | MAP1LC3B | PLXND1 | FKBP5 | PLA2G2E | BIN1 | DNAI1 | COLEC10 | SOCS5 | DEFA1B | ARL8B | NDUFS2 | STK10 | SON | CAPZA1 | LOC100420831 | ITGA1 | RNASE6 | FASN |
| FBXL19 | CCT2 | SLC6A14 | CORO1C | HRH2 | PNKP | GCNT1 | LGALS8 | AFDN | LRP2 | TRAP1 | LPIN1 | CYP17A1 | DBT | SLC6A3 | HPGDS | PPP2R5C | EEF2 | FNIP1 | TONSL |
| RACK1 | ZMPSTE24 | PTPRN2 | JAML | PIGN | PFKM | AVP | TSC22D3 | RNF6 | AMN | MTR | CETP | AQP3 | PPP1CC | TUFM | FOSL2 | NOP53 | TK1 | UBE2K | SLC6A4 |
| CLDN4 | UBE3C | RLIM | C1RL | MICU1 | DNAAF1 | PRKCI | PDLIM2 | TSPAN12 | PRSS23 | GON7 | BUB1B-PAK6 | NDP-AS1 | DCR | LOC106804613 | LOC110006319 | NKS1 | DUP22Q11.2 | TOP3A | TACR2 |
| S100A4 | ADGRG5 | CPB2 | CSN2 | GLI2 | NUP35 | DTX3L | CD101 | TRDJ1 | TRDD1 | CRACR2A | DARS2 | EIF3H | CPA1 | TRPV1 | USP29 | PHOX2B | FBXO7 | LPRS6 | RWS |
| DUSP6 | POGZ | TARM1 | SRP72 | HTR3A | CLCF1 | PEBP1 | CACNA2D1 | CFAP410 | CEACAM7 | PCNT | CUL3 | PSMD8 | CRBN | PHGDH | RAP1GAP | TNC | UGGT1 | SIGLEC6 | STX16 |
| BLMH | ZIC2 | RPL26 | PRMT7 | TYMP | SNX10 | GFI1B | PDXK | PPP2CB | IKZF2 | TBL1XR1 | NABP1 | RABL3 | NBAS | EEA1 | NAT2 | PFKFB2 | ABL2 | TRAPPC10 | COLCA1 |
| RNF2 | ANO6 | ADCY10 | RSPH9 | ABI1 | BTN2A1 | DOK3 | SKAP1 | PROM1 | ARPC3 | MAD2L2 | PDE12 | PYDC2 | ACLY | RPS6KA5 | RAB5A | TBC1D10C | NEURL3 | PPP2R1A | MVP |
| PRSS16 | PA2G4 | OGG1 | TANGO2 | H2BC15 | SLC46A2 | SFRP1 | HERC2 | POLI | NTS | LTBP3 | TPT1 | CNOT7 | HPGD | RARG | CXCL17 | TRIM34 | ILDR2 | EXOC2 | FARSB |
| RHOD | DUSP3 | CKLF | SEMA3C | ANK1 | PLCB4 | PSMD10 | UBE2T | FANCE | WNT10B | AP2M1 | NRG1 | LDB3 | CHST4 | GNB3 | COCH | TRAPPC11 | CYP7A1 | TNFRSF10C | MAPKAPK3 |
| GRIN2B | IGFBP3 | SPARC | TRAPPC1 | HMGCL | GNAO1 | POLA1 | ARPC5 | CYP1B1 | TUBB4A | HGD | IKZF4 | CRISP3 | DGCR5 | PTPRD | LY6E | SHOC2 | MCM5 | DPM1 | HDAC11 |
| PLD4 | STX7 | HDAC6 | MYOM2 | BRD2 | CAV3 | PDE3B | KLHL22 | KIR3DL3 | FAF2 | CDH3 | SH2B1 | PDCD10 | USP27X | PRPF3 | SLC26A9 | TPM3 | FBL | FOXI3 | MUC19 |
| COX5A | DDX21 | UBE2A | COL18A1 | DSG4 | CALCR | MPP1 | TFPI | IGSF3 | CTNNA3 | GSK3A | NFIL3 | CFC1 | LSM2 | SLC27A2 | HPX | ROR1 | SH3BP2 | NUP43 | AK7 |
| F12 | LCE3B | SUPV3L1 | CCNE1 | HCFC2 | CCT5 | EXOSC2 | NTRK3 | GANAB | FOXC1 | CAPN3 | FUS | DPP8 | TOR1A | VPS13B | MMAA | GLIPR1 | METRNL | OPRK1 | FEZF2 |
| EVC2 | POFUT1 | NAB2 | KPNA1 | UBE2E1 | CCDC22 | TBL2 | RYR2 | WNT3 | DAB2 | SSRP1 | H1-4 | SNRPA1 | CD99 | GPR68 | FLOT1 | SPTB | DNAJB11 | UBXN1 | IGF2BP3 |
| SRP68 | H3C14 | ARAF | ILK | TSPAN32 | COG5 | MYH11 | EIF2A | CNIH1 | UBE2M | XPO5 | TLK2 | SLC27A4 | GAPVD1 | AFG3L2 | RHOC | ABCA7 | PAX2 | DAPK1 | SRD5A3 |
| SCD | QDPR | REV1 | FUBP1 | VAPA | EIF4A1 | RAD21 | PSG4 | FAS-AS1 | ATL3 | SIK3 | CSN3 | WNT4 | SUMO1 | DNAH11 | VPS33B | DNAH5 | CCDC40 | RANBP1 | KDSR |
| CDK12 | PINK1 | TFF2 | TUBA1C | OTUD4 | IP6K2 | DEFB114 | GFRA1 | BID | COL11A1 | PDIA6 | HBS1L | EPHX1 | MTREX | LONP1 | SENP7 | CTRC | NSD3 | DDR2 | SYVN1 |
| FAM111A | NCOA3 | PSG2 | BARD1 | UBN1 | HDAC3 | BIRC7 | HNRNPD | IFT20 | G3BP2 | CERS3 | FAT1 | NIPBL | ANKRD11 | MC4R | HNRNPH1 | EHBP1L1 | DNMT3L | NUP188 | EWSR1 |
| LORICRIN | SEPTIN5 | RAB6A | ACVR2B | TRIAP1 | ST3GAL5-AS1 | SLC30A5 | ACADM | PYHIN1 | PCM1 | PAEP | NEK9 | BHLHE40 | TRAF3IP2-AS1 | BTN1A1 | DMP1 | RNF26 | KLK15 | NR1I2 | TDGF1 |
| NDC1 | SLC5A2 | PHF19 | SLC18A3 | MLKL | SLC26A2 | ATMIN | NPAT | NDRG1 | AHSG | VIPR2 | MID2 | S100A1 | HAPLN1 | RAB32 | XCR1 | LOC117307477 | STX17 | AGT | L2HGDH |
| USP17L2 | FOXA1 | LIME1 | LMO7 | LRP6 | RNF216 | AK5 | IREB2 | COG1 | KCNJ6 | EIF4G2 | DDB2 | ADCYAP1 | LRRFIP1 | PNOC | IRAG2 | GLI3 | RPS7 | CGB5 | TRAJ18 |
| UXS1 | FAF1 | ADAMTS2 | RBBP4 | RHOF | KLF4 | NDUFA9 | AHNAK | TH | MORF4L1 | MIR33A | POU2AF3 | IDS | HMMR | DEFB106A | GPR89A | ADARB2 | LCE3C | TGM5 | DUSP10 |
| NRON | SIPA1 | TRIM39 | PLSCR1 | DLG5 | RNF128 | SIX1 | HK3 | AQP5 | NACA | PDCD6IP | STAU2 | MSLN | H3-3B | GMNN | KYNU | ENO2 | SPTBN1 | FSHR | CA9 |
| CLEC2A | ATAD1 | TRIM65 | GLIS3 | MMP19 | CRHR2 | IAPP | MAP4K1 | MT-TF | MT-TH | ADAMTS14 | CD82 | STIP1 | GNAI1 | AP2A2 | SIAH1 | ANKRD1 | DUSP2 | UTP4 | COL6A1 |
| UBE2V2 | FMO3 | DEFB136 | PJA2 | HSPE1 | CASP12 | MLEC | SIGMAR1 | DRD3 | ABCC9 | SEC61G | LRRC19 | B4GALNT1 | SPTLC1 | POLR2B | ABI2 | TBCK | GART | RBL1 | ZMIZ1 |
| RAP1GAP2 | RPL15 | EXOSC10 | KLF1 | P2RY13 | GNB4 | ERLEC1 | UACA | TUBB2B | DEFB106B | MCEMP1 | RPGRIP1L | SLC30A10 | TP53I3 | TRIM3 | TRIM59 | DNAAF5 | PIGM | SLC25A37 | MKRN3 |
| ERO1A | KIF2C | TPM1 | CCDC124 | CALML5 | PDE5A | SCO2 | MIAT | MARCHF9 | FKBP1A | BAG5 | CDK5 | CYP3A5 | CSH1 | ID2 | CTSZ | CDC34 | ATP6V0E1 | HTRA2 | STT3B |
| SEC13 | XRCC2 | SNRPE | TNNI3 | YPEL5 | PEX5 | PRCP | KAT7 | PYY | SMARCE1 | EIF4E2 | FGF14 | SETD1A | TASP1 | TNMD | IGF2BP1 | SLC7A2 | HABP2 | ANXA6 | EME1 |
| PC | BBC3 | TSR2 | MAGI2 | RING1 | GBP6 | PPARD | FAN1 | KAT6A | MTCP1 | TRIM49D2 | CYP51A1 | PGAM5 | WASF4P | PIWIL4 | EXOSC3 | PELI2 | NUP54 | PALB2 | PTCD3 |
| FIG4 | POGLUT1 | CASP14 | CRISPLD2 | PVALB | DPP10 | GADD45G | KIF2A | THEM4 | SHBG | MACROH2A2 | RAB23 | GLIPR1L2 | SRCAP | ADIPOR1 | MYBL2 | HOTAIR | CHAF1B | SMURF1 | UBASH3B |
| UCP2 | SNORA31 | MBL3P | NR4A3 | ERCC6L2 | CUL4A | GCLC | LPCAT1 | STAU1 | GMPS | SUFU | CTR9 | DCUN1D5 | COL6A2 | CRB2 | MIR195 | POLE2 | IBTK | TNNT2 | MBD2 |
| COX4I2 | DENND1B | ABHD5 | MNX1 | PRKACB | PRDX2 | LEMD2 | RAD23B | CAPZB | MARCHF5 | TTLL12 | KRT71 | YTHDF1 | SPRED1 | CENPE | SMARCC2 | RNF185 | FXR1 | LNPEP | LAMTOR1 |
| TOMM20 | ATG9A | MYF5 | AK1 | NUDT6 | ACSL3 | ROM1 | LAX1 | H2AZ1 | HMGA1 | PPARGC1A | PCK2 | BCKDHB | GLRB | COL13A1 | TWF1 | SNX9 | DEFB108A | FKTN | MAFB |
| DEFB107A | FGF19 | BPIFB6 | MARCHF7 | LIMK2 | SPTBN2 | EMP2 | NAGS | GCDH | MMP7 | MCAM | FBXO11 | TACC3 | ACTR1A | WDR26 | PMCH | NCOA2 | GNG12 | NTAN1 | HPSE2 |
| TUG1 | IARS2 | TMEM70 | MDM4 | ANKRD36B | C9orf72 | TRBV6-2 | CHRNA3 | XYLT1 | EXOSC6 | PTGDS | UBE2G2 | UBR2 | SORT1 | MBTPS2 | DEFB107B | SKIC8 | DOK1 | MYL2 | MYH3 |
| SIRPB1 | ESRRB | CCL4L1 | SYNE1 | SLC9A3R1 | LTB4R2 | SOCS6 | CSNK2A2 | SMIM3 | FLVCR2 | ISL1 | THOC6 | MED13L | GOT1 | GLMN | DNAAF11 | TUBG1 | POU5F1 | LEPQTL1 | RARS1 |
| CUX1 | FOCAD | RALB | PDE4C | S1PR5 | ADARB1 | PBRM1 | AREL1 | BBS1 | AGO2 | BMS1P20 | SSTR2 | GGT5 | ZFP36L2 | RC3H2 | DNAJC13 | BAG1 | DNAJC21 | PRKAR1B | ARL3 |
| CRK | ITGB5 | PPT1 | KAT2B | H1-3 | SPAG11A | TRIM49B | TRIM77 | TRIM49D1 | PTRH2 | PNMA2 | ZCCHC8 | PLAG1 | FOLR1 | KIF20A | POLR3GL | ATG14 | KL | PTN | PTMS |
| BLTP1 | WSB1 | SEC63 | SSC4D | RRAS2 | MMP25 | GRIA3 | CEBPG | SOX6 | TRIM58 | MLLT10 | STK11IP | FGFBP2 | NPPA | FANCB | MGAM | AP1M1 | LUC7L2 | TCN1 | OTUD1 |
| N4BP3 | EXOSC7 | C1D | VIPAS39 | MAP1LC3A | MAEA | TSPAN6 | CUL2 | TUBB6 | NSA2 | RAB5C | ANP32B | NAA10 | ERCC8 | ZMYND10 | TMPRSS6 | MAN2B2 | CACNA1A | AHI1 | FNDC4 |
| EYA1 | SCART1 | NOX3 | RPL7 | SNX27 | YBX1 | PLCL2 | PIM2 | JDP2 | LOC108167311 | LOC108167312 | HSD3B2 | SPOP | NUP58 | SARS2 | ANAPC11 | SNRPD3 | EVL | LINC00328 | EPHB6 |
| HSPB8 | H2BC12L | H3C15 | PCLAF | LRSAM1 | EFNB2 | HHEX | HNRNPK | BOLA2B | PLAAT4 | SETDB1 | ITPKC | UBTF | MTA2 | RASGRP4 | GLRA1 | SLC2A10 | BPTF | LUC7L | KANSL1 |
| MYBPC3 | GCA | HMHB1 | FAAH | IGKV1OR2-108 | JMJD6 | MYPN | STXBP1 | SRSF1 | HTR2A | CGB3 | ZNF638 | H3C4 | H3C6 | H3C13 | ACTR1B | CHST14 | ARF6 | EN1 | RTTN |
| ADSS2 | CERK | PSMA8 | FERMT2 | TDP1 | PTTG1 | STEAP4 | OR10J3 | MSTN | MCM7 | MSRB1 | CCNH | COPS6 | MTHFD1L | MT1A | BCAP31 | CACYBP | REST | ZP1 | MYH2 |
| PMAIP1 | NOX5 | AMPD3 | MFAP4 | CLPTM1L | TM9SF2 | OGDH | TRPA1 | GOLPH3 | TEP1 | MT-ATP6 | RAB11A | IGLV@ | ENAH | BAIAP2 | WASF3 | MAPKAP1 | FBLN5 | RPS28 | CLEC14A |
| CERT1 | NFIX | ACTG2 | ARHGDIB | B3GALT6 | NEXN | PPP6R3 | SERINC3 | PGAM1 | BTN2A2 | KRT7 | ADK | RPS6KA1 | C1GALT1C1 | PRPF8 | FOSL1 | PRKCSH | SRSF5 | MGP | ADAMTSL1 |
| RAVER1 | HECTD1 | ASB1 | SHB | ZDHHC6 | CTNNA2 | ADH1C | ABCD3 | ACAN | GLI1 | SENP3 | TRMT2A | AMPH | ALPP | ANAPC2 | NOC2L | TOP1 | VARS1 | TOP2B | VDAC1 |
| HNRNPL | HP1BP3 | HCLS1 | ERG | CKAP4 | DCTN1 | LRRC14 | AQP1 | NCK2 | SLC36A1 | DEK | PLEKHM2 | NOTCH3 | ANLN | AHSP | BMP1 | ITGB8 | ETS2 | TRMU | PIAS3 |
| SQLE | RPS16 | NME1 | MYL12B | KIR2DL5B | KIR3DP1 | SNX20 | STYK1 | LMO4 | RAB14 | HERC1 | ACAD11 | PEG10 | CHRNB4 | CDC5L | FBXO6 | SDC4 | DNAJC2 | TMEM59L | GH2 |
| UCHL1 | VPS35 | TARDBP | DHX33 | LRIG2 | GJB4 | DNAAF4 | MBD5 | ODAD2 | DRC1 | APOBEC3A_B | IVD | DEFB110 | HOXA13 | EIF4B | HACD3 | DROSHA | SHPK | IDE | IMPDH1 |
| POP1 | PPIC | PCYT1A | IFT140 | QPCT | TRAF7 | SND1 | ARFGAP1 | CPOX | SCN2A | C17orf107 | BLOC1S5-TXNDC5 | EEF1E1-BLOC1S5 | LOC112533672 | LOC121587541 | GRD2 | HNRNPDL | RNU7-1 | SOCS4 | DEFB105A |
| RPTOR | PIAS4 | SNRPB | GNB1L | PTGES2 | PAFAH1B1 | NPAS2 | DEFB133 | RPL27 | DBH | SEPTIN2 | APOBEC4 | CFL2 | GPC4 | ACAD9 | MYLK3 | DDX39B | ZRSR2 | KRT74 | NOC3L |
| RNF113A | UTRN | ASCC2 | KIF23 | TRIM64B | TRIM64 | TRIM51G | IGF2-AS | WWTR1 | DEFB116 | DEFB113 | LILRA6 | TUBA4A | TFAM | PSEN2 | TDP2 | SMN1 | STK38 | DYNC1I1 | GTF2H5 |
| PPM1D | DYNC2I2 | TRIM51 | TRIM49C | MGST2 | PI16 | MAGED1 | GSTZ1 | DNM1L | DNAH8 | AKAP13 | CCN1 | UBE2C | FGF4 | FADS2 | UPF1 | MAML1 | NAPEPLD | GPX2 | SLC35D1 |
| UBE2F | RPS6 | ADAD1 | DSC2 | SC5D | PDE4B | SMCR8 | CEACAM20 | LRRC8D | STRA6 | TRIM69 | ACTN2 | FRAS1 | VASP | POLR2E | ZBTB1 | LAYN | BMP8A | ATF7 | NME2 |
| FTO | ASB2 | IGFBP5 | ARL14EP | ENOSF1 | IBD2 | IBD3 | IBD6 | HTRA1 | SLC25A12 | SPDEF | EAF2 | SRA1 | RBM38 | VCAN | MIR183 | NCKIPSD | RAD17 | SLC30A1 | SUSD4 |
| DCTN2 | TOPBP1 | TAF1A | RFX6 | RPL18 | RPL31 | APC2 | ADPRM | CACNA1B | DEFB105B | ATP4A | PWP1 | TRIM50 | HK2 | SLC39A10 | CDAN1 | BLZF1 | HIVEP3 | NEDD9 | SCARF1 |
| TWF2 | KIF3A | PJA1 | RNFT2 | COBL | GTF2IRD1 | ALDH1A2 | TPD52 | SEC61B | AUTS2 | FBLIM1 | LECT2 | XIST | EFHD2 | USF3 | DEFB128 | MPZL3 | UBE2E2 | GPATCH3 | PTPN14 |
| AGR2 | PIP5K1C | COG8 | RAPGEF1 | BMPR1A | NRP2 | AXIN2 | FGFRL1 | PPIL1 | CLDN10 | MAP2 | TRG | ARC | NODAL | STT3A | INHBA | TGFBI | ATIC | CPNE3 | MIR186 |
| LRRC8E | EDA2R | HBG1 | DEFB135 | MIR491 | SEPTIN7 | S1PR4 | ERLIN1 | CH25H | CCAR2 | HSH2D | FGF1 | SETBP1 | IL1RAPL1 | RPS15A | UBA2 | GHSR | KRT2 | NEK10 | DPF2 |
| STOM | LRRC8C | LEMD3 | UBOX5 | CC2D1A | MT-ND5 | BLOC1S1 | PTPN7 | DEFB104B | CELSR1 | MPV17L2 | FAM53A | MED15 | GNPTG | NRROS | CDC45 | UGT1A1 | MTDH | AGBL3 | SAA4 |
| UBE2E3 | MDH2 | BCL2L12 | SLC48A1 | DYM | SLC41A1 | DHX29 | RPS3 | NPHP3 | LSS | SUMO4 | HMBOX1 | PABPC1 | FGF5 | CACNA1S | FOSB | DUSP22 | CCL4L2 | RPS27L | PRRC2B |
| AAMP | OPLAH | PPP2R5D | KLF2 | MMP24 | GPR18 | TPSB2 | IGKJ | IGLJ | FOXM1 | CTSW | IPO5 | STK19 | AKAP1 | CAP2 | PRPF31 | FOXH1 | RIC1 | DAPP1 | ZP3 |
| FOLR3 | BLOC1S3 | EEF1A2 | CDKAL1 | YARS1 | BAHD1 | SLC22A6 | SCT | BNC2 | MNS1 | MYO1H | AMPD1 | WAC | SORBS1 | EPSTI1 | HSF1 | CAPN5 | EPM2A | GNAI3 | DDRGK1 |
| NLRP2B | ATOH7 | ADIPOR2 | PTPN5 | PYDC1 | EDC4 | MKS1 | TMEM237 | PMS1 | RBBP5 | HNRNPU | ZDHHC18 | PEDS1 | USP22 | ATP1A1 | CEP152 | IER3 | GALNT3 | CCR5AS | CCDC92 |
| LY6G6D | EPOR | LSM8 | RAMP2 | DGUOK-AS1 | SNHG22 | CALCRL-AS1 | IDDM8 | IDDM3 | IDDM4 | GCCD3 | HT | IBD4 | IBD7 | IBD8 | IBD9 | IDDM11 | IDDM13 | WWP2 | MRGPRX2 |
| IDDM6 | MTTP | LMO1 | NAPB | AA2 | ASRT3 | ASRT4 | ASRT6 | ASRT8 | EA3 | EA7 | EA8 | EOE1 | EOE2 | GRD1 | IBD11 | IBD12 | IBD15 | IDDM15 | IDDM17 |
| IBD20 | IDDM7 | LOC107032825 | NIDDM2 | IBD24 | IBD26 | IBD27 | IDDM18 | IDDM23 | IDDM24 | LRSL | MBNP | MTBS1 | NBLST4 | NBLST5 | NBLST6 | NBLST7 | NIDDM4 | IBD16 | IBD18 |
| PBC4 | IBD21 | IBD22 | IBD23 | TST1 | TST2 | HNF4A | LAMB3 | APOC2 | TRH | CLDN7 | GNGT2 | RAB1A | MRPS22 | POLQ | ZNF365 | CLCA2 | OGN | PBC2 | PBC3 |
| TRIM36 | PBC5 | PSC | RC14S | SEH1L | ASB7 | PLPP5 | ADAT3 | CFAP300 | CEBPZ | KLHL2 | DSN1 | FLNC | H6PD | MPLKIP | MARCHF3 | SERPINI1 | SMURF2 | RNF41 | RAB18 |
| ZBTB7A | SERPINB12 | ARMC8 | PBX1 | POLR1D | APEH | KCNA3 | HSD3B1 | PTPN9 | TRIM45 | PSG9 | CSPG4 | RPLP1 | ARID4A | HSD17B1 | TIMP4 | IQCB1 | TMPRSS15 | CITED2 | DIP2B |
| COMMD10 | FUT7 | SIM1 | MIR301A | PAK4 | SF3B2 | HNRNPC | G6PC1 | FOXF1 | SAA2 | PKP3 | PAK5 | SOX8 | IGF2BP2 | HOXA3 | ANGPTL4 | PITX2 | PLIN2 | PER3 | GAB3 |
| CLDN5 | GRPR | GABRP | TERF2IP | PHYH | PEX7 | NEK8 | B9D2 | FGD1 | CLDN6 | CALU | TIPARP | TGM6 | SFXN4 | SSX5 | FBXO17 | GSDMA | OGT | FOXJ2 | SDHC |
| AP1G1 | ACACA | JMY | HNRNPF | PSPH | SH3PXD2B | TBC1D8B | TRIP12 | CACNA1G | S100A7A | RRAS | MMRN1 | ING4 | CPQ | DYNC1I2 | EFEMP2 | RSPH1 | CNNM4 | MIR182 | ASXL3 |
| NUP50 | H2BC6 | PRPF6 | BCL7B | CEP76 | KIF5A | PUS10 | CRNN | MYF6 | GNA15 | TRIM46 | FMN1 | KLK11 | RNF7 | UBE2L6 | KAT6B | CHAMP1 | MCCC1 | SCNN1G | A2ML1 |
| MYBPH | EPHX2 | TRIM11 | RBM22 | RPLP2 | ATL1 | MTX2 | H1-2 | ATP5IF1 | JADE2 | KCNJ10 | NPPB | FOLR2 | MYL12A | EPHB1 | CDC37 | GAS8 | PHF20 | CAMKK2 | SRGN |
| ATAD3B | EIF3C | ASL | SFTA3 | PGD | NFASC | FFAR1 | SRPK1 | WDHD1 | CMAS | SAC3D1 | ZDHHC11B | CHIC2 | FDX2 | NCOA1 | SMARCAD1 | RALBP1 | PNPLA2 | MAP3K9 | DEFB134 |
| TRIM43 | MGA | SIRT3 | LIPT1 | TDRD3 | TNKS | AKR1B10 | CD300LG | SEMG1 | LEAP2 | CLEC12B | EIF2AK1 | EYA2 | LRP8 | ITGB6 | WASH6P | PDE8B | SCP2 | RFWD3 | DOLK |
| GRHPR | H2BC8 | HRNR | HTR1A | SMG9 | PLN | PLTP | FABP2 | GBX2 | CHST3 | THBS4 | TLX1 | BPIFA2 | PFKL | IQGAP2 | ADAM19 | KIFBP | TRIP10 | AEBP1 | USO1 |
| RAB8A | NXN | MORC2 | SHROOM4 | CLEC18A | ADGRB1 | GABARAP | TNK2 | PLXNA1 | ELAVL4 | LITAF | SSR2 | PARP12 | S100A14 | TFAP4 | EHMT2 | MIR17HG | FLCN | ACTN1 | PRSS56 |
| KHSRP | CBR1 | CYFIP2 | FIS1 | MIB2 | TUBA3C | VPS35L | BBS5 | CRY1 | CHST1 | ATP11C | DNASE1L2 | AKAP8 | FAU | EIF4A2 | POP4 | FZR1 | CPN1 | WDR45 | KRT86 |
| AP1S1 | NUDT10 | TAS2R38 | ARIH2 | COG2 | TSEN34 | BCLAF1 | SLC25A3 | PTGER1 | H2BC10 | TRIM75 | IGHV1OR21-1 | IGHV1OR15-1 | GSTT1 | ANGPTL3 | KAT14 | MIR16-2 | SREBF2 | SRSF7 | KIF3B |
| GORAB | RN7SL1 | PAX4 | CSPP1 | SCYL1 | B3GNT2 | NAP1L1 | HCFC1 | TOR1B | SLC30A8 | GCNT2 | CCDC28B | MUC20 | TUBA3E | TUBB8 | DYNLL2 | RAB4B | ZNRF2 | LRIF1 | RSPH4A |
| TPR | PNPLA1 | MT-ND4 | STOML2 | GDF1 | SPECC1L | BTBD1 | TRIM9 | RASA2 | CRYAA | ALYREF | LCE3A | SPAG9 | KISS1R | USP13 | GOLT1B | DLEU1 | UCHL3 | MT-ND2 | RPL35 |
| CST6 | ZNF76 | POU1F1 | COX10 | SLC2A11 | TMCO6 | RPS4XP13 | HSPA2 | RNF217 | TOMM40 | ERF | CAD | CDC6 | ICA1 | CRB1 | MATN3 | HAP1 | MT-CYB | MEOX2 | ZFYVE16 |
| DOCK7 | CARS1 | ATG9B | SCAF4 | CAP1 | ATOX1 | HECTD2 | MOSPD2 | C6orf120 | SERPINA7 | RAB2B | PPP4C | SPATA2 | CHD8 | TPBG | TRPM7 | LNX1 | HIVEP2 | GSTA2 | DGKZ |
| RIPK4 | ABHD13 | UBR1 | UBE2H | SLC29A2 | CDK5RAP2 | TWNK | PABPC4 | F7 | AKR1C3 | WDR77 | DDX50 | ETV7 | AMBP | ADGRE2 | P2RY6 | HDAC5 | JAG2 | NR3C2 | HIF1AN |
| ARPC1A | CCDC116 | NELFCD | EIF4EBP1 | LARP7 | NKRF | GDF2 | SSR1 | SCARA5 | MARS1 | ID3 | RTCB | KRT33B | PROK2 | MFN2 | GPHN | TBX6 | KCTD7 | ZBTB17 | CPS1 |
| PFKFB3 | MAN2C1 | COPB2 | TSPAN7 | BBS12 | SLC30A7 | CLDN9 | GTF2E2 | SRSF6 | SNX19 | CLSPN | RBM15 | SHANK3 | GLCE | DPP7 | UBE2Q2 | DZIP3 | KLHL13 | ATF6 | PNMA5 |
| NLK | DHODH | NEK2 | TMEM138 | ARRDC4 | MAFK | REG1A | UBE2J2 | PPP2R5A | PIK3AP1 | ADGRG3 | SERPINB6 | GLRA2 | FGF3 | PAPSS2 | STK36 | CDK10 | NSMCE2 | TMEM179B | MYL6B |
| SPTBN4 | BHMT | RGS5 | PCTP | MCIDAS | ESRRA | MSI2 | TMEM209 | RINT1 | NOG | TBC1D24 | PPM1B | ASGR1 | H1-5 | SPATS2L | UCN | P2RX1 | ADH4 | HEPHL1 | NME5 |
| DEFB130A | DOHH | KPTN | GAS2L2 | GLRX5 | PIP5K1B | FXR2 | PTPRF | HAS1 | RPP30 | KCNJ12 | OXT | MATK | PARP14 | CIB2 | CSRP2 | RAP1B | HES1 | OFD1 | PES1 |
| RBM7 | LPA | H3C3 | WNT1 | CCNDBP1 | BICC1 | NAB1 | RGS14 | CMAHP | SMPD3 | DOK2 | IST1 | LRG1 | NLRP8 | MT-CO3 | SGK1 | UBR5 | THOP1 | PLS1 | MEF2A |
| MGRN1 | TXNDC2 | SLC25A38 | PRPSAP1 | PLEKHO2 | ASB16 | ASB4 | NME1-NME2 | RAB44 | EPPIN-WFDC6 | GRIA1 | F13B | USP21 | PDCD7 | PPP1R14B | TRIM61 | TRIM64C | TRIM43B | QSOX1 | TMEM30A |
| IGHV3OR16-10 | SERPINB10 | FBXW2 | HERC4 | IGHV2OR16-5 | IGHV3OR16-12 | IGHV3OR16-13 | IGHV3OR16-8 | IGHV3OR15-7 | PDGFRL | GPIHBP1 | CLIC1 | PCBP1 | DEFB108B | RAB9B | NKAP | DDX46 | CORO1B | IGHV4OR15-8 | IGKV3OR2-268 |
| YTHDC2 | IGHV3OR16-17 | NFILZ | IGHV1OR15-9 | CAMK1 | EPN1 | ANXA4 | AKR1D1 | PDE11A | CLDN16 | RPGR | DNAI2 | ZNF750 | MT-ND6 | MT-TL1 | MT-TW | SDC2 | SLC39A6 | MAP3K2 | USP4 |
| GPANK1 | TBCD | NLRP7 | ZC3H12D | MDGA2 | HSPH1 | ROS1 | GHITM | NMT2 | MIR125B2 | CPSF6 | CYTIP | DEFB108C | SLC15A1 | RNASE8 | PDLIM1 | ASCC3 | GOLGA2 | ANXA3 | LAMC1 |
| SMAD9 | ENAM | USP10 | FOXD3 | SLC39A2 | PIK3IP1 | PNO1 | NCOA5 | RBM3 | TMUB1 | HELQ | ZBTB46 | TMEM150B | GOSR1 | IFNW1 | ZDHHC8 | ITFG1 | B9D1 | SEM1 | NLRP14 |
| PROX1 | CCT3 | ITPA | ZNF699 | LINC01187 | LINC02040 | LOC105377125 | SEZ6L2 | LIPH | POLR1B | TMEM129 | SCN10A | SCN11A | SGCG | USP19 | MGAT3 | TAC3 | XAGE1A | TMEM216 | ITGA9 |
| H2AJ | FKRP | C5orf58 | ADAMTS9-AS2 | RCAN2 | HMSD | DOCK1 | ATP6V1C1 | ZNRF1 | FBXO4 | FBXL5 | FBXW8 | FBXW10 | HERC6 | FBXW12 | FBXW9 | DEFB123 | DEFB125 | TFF1 | EIF3M |
| CHD6 | SH3GL2 | MMP11 | HMGN1 | GABARAPL1 | TRIB2 | COMMD8 | E2F2 | GPR55 | RTRAF | PPP2R2B | TRPV6 | XRN1 | ADAM15 | LTBP4 | ROGDI | ODAPH | RASSF5 | PENK | RIMBP3C |
| BMS1 | PDS5A | CKS1B | MT-ATP8 | DEFB121 | DOCK6 | SH2B2 | TNPO1 | RLBP1 | NES | A2M | BMPR2 | DPYD | MTRNR2L8 | NR1D1 | BNIP3L | GABPA | BMP5 | MIR30D | TRD |
| MIR217 | SYNJ1 | CYP11B2 | MYLK2 | GRK2 | HSD11B2 | ATG3 | RPS2 | KLRA1P | RASGRF1 | EBP | WDR35 | ALG11 | FAM83H | UROD | KMT2B | ALPI | S100P | CSGALNACT1 | MIR381 |
| LEO1 | DDX5 | CDON | DISP1 | TRIM17 | MCHR1 | SEC14L2 | AFAP1 | MYRF | TPPP | ST3GAL4 | SCG2 | PIWIL1 | MIR296 | ZRANB3 | RBM34 | DDX23 | EIF2S2 | MRAS | OVCH2 |
| CYRIB | CBFA2T2 | SETD7 | RPL30 | WARS1 | SIRT2 | PAK6 | ABRAXAS2 | RAB38 | TAGLN | RASSF1 | S1PR2 | CNTFR | ATP1A3 | IGFBP6 | CLCA4 | SIAH2 | FABP1 | FCRLA | YTHDF3 |
| S100A11 | ECRG4 | OLIG2 | GOSR2 | SAT1 | SLC23A2 | SLC23A1 | THRIL | RMI2 | NMNAT1 | PYCR2 | PGRMC2 | SWT1 | MIR26A1 | TXNDC15 | YBX3 | SLC25A4 | CCT4 | RBM4 | NFX1 |
| YIF1B | GNL3 | RPN2 | RBM28 | ERVFRD-1 | COL4A6 | PPIG | SYP | H4C9 | USP5 | VDAC2 | ALAS2 | AKAP9 | MTUS1 | MZB1 | SH3RF1 | PRMT1 | MED25 | TDG | MIR2909 |
| ARL6 | MUC12 | ATXN3 | PIGU | SSC5D | KLK5 | DYNLT1 | ACTL6A | SESN1 | MBOAT7 | NDRG2 | PLA2G15 | FST | CAMK1D | LTC4S | SLC19A3 | CNOT1 | S1PR3 | AOC3 | PER2 |
| RPL10 | CDIN1 | MOK | STS | LOC108004536 | GTPBP4 | NFE2L1 | CPVL | HOXA5 | TRAPPC9 | LRRC25 | GNRHR | GUCY2C | PTPRK | CSTB | ATP6V0D2 | PLAC8 | CYSTM1 | PGK1 | LGALS4 |
| IGSF8 | NHLRC2 | SEC23A | LOC105980078 | H3C11 | H3C12 | H3C10 | H3C8 | H3C7 | HBEGF | RAPGEF4 | DEFB132 | TSR1 | CSTF1 | LIPC | SENP1 | ING1 | SETDB2 | ASB5 | MDH1 |
| RPL39 | CIP2A | NCKAP1 | DRD4 | LCN1 | ADAMTS5 | CTF1 | CTRL | SLC10A1 | VPS13A | CLCN2 | STAM | WDR36 | LAS1L | SNTA1 | ADORA1 | MOCS3 | ERLIN2 | ATP5MG | MIR628 |
| TCHH | EIF2B5 | SMARCC1 | ZBTB7B | GNG10 | MEF2B | CCNB2 | EDIL3 | TAGLN2 | HOOK3 | MIR212 | TLR12P | GM2A | SMCHD1 | MAGEC1 | FABP12 | ARID5A | ANXA7 | MOBP | AK9 |
| ZNHIT6 | MCM3 | DSE | ABHD12 | CSNK1A1 | TAF1 | GCGR | KIRREL1 | KTN1 | IER3IP1 | SEMA4B | ELOVL4 | SCG5 | ASH2L | PPP1R13L | LOXL2 | SLURP2 | CD163L1 | LSM3 | PDZD8 |
| KDF1 | SRPK2 | ANXA11 | NCF1C | PTP4A3 | HCAR1 | ESYT2 | CCDC78 | HNRNPM | LRRFIP2 | ZNF804A | EIF3A | NOP2 | EIF4A3 | GAS1 | ABCB7 | PNKD | CYP20A1 | NDE1 | P4HTM |
| HOPX | BDKRB2 | AARS2 | SERPINE2 | WHAMM | FRK | PRG3 | SP3 | SLC9A4 | GMPPA | TNRC6B | MT-CO2 | ADRB1 | RALGDS | CSNK1E | RMI1 | GREM1 | CPA3 | KCNH1 | PPFIA1 |
| TNFRSF19 | CCDC88B | PF4V1 | CALHM6 | ACVR2A | SLC12A4 | GATAD2B | SLC24A4 | CDCA7L | TSPAN2 | PRPS2 | EIF3D | PLRG1 | PRMT6 | CC2D2A | LAPTM5 | TIE1 | MIR100 | ATP5PO | RHEB |
| EBF1 | PSAT1 | RSL1D1 | KHDRBS1 | PBXIP1 | MICAL3 | OTUD3 | TMEM109 | OAT | PLAA | MTHFD2 | SIT1 | XAF1 | SHFL | PLS3 | BPIFB4 | CCT6A | BMP10 | MAD2L1 | BCL2L14 |
| FUT3 | GLG1 | WLS | DSTN | MRTO4 | EGLN3 | KDM5A | SULF1 | CNOT3 | CNMD | GPX8 | PPT2 | GPR174 | MAP7 | CHTOP | H4C3 | VANGL1 | TAL2 | PEDS1-UBE2V1 | GNG4 |
| ATP2B4 | LMAN2 | NOP56 | AMBN | TNFAIP8L1 | RDH11 | PON3 | RNF20 | ARHGEF5 | SBSN | CTNND2 | DLX3 | AP2A1 | RHO | TCL1B | RAB3GAP2 | TM2D2 | PARK7 | MATN1 | PTGES |
| AKNA | DDX39A | NTF4 | DUSP14 | PACS2 | RECQL5 | H4C5 | VLDLR | FBLN1 | POLRMT | HIP1 | EEF1E1 | SMTN | ZC3H12C | BNIP1 | SMC6 | RWDD1 | P2RY11 | EPAS1 | SNX14 |
| PI4K2B | MIR135A1 | FIRRE | MYNN | NUTM1 | PRKAB2 | SFN | GHRHR | PRKACG | AHCTF1 | SLC12A3 | TOM1L2 | FBXL20 | RTL1 | PTK6 | FAM20C | EBNA1BP2 | SSX2 | GSDMB | KDM5D |
| ABO | KRT6A | NEK1 | DDX52 | NR2C2AP | TMEM185B | AP3S1 | AUP1 | TPCN2 | SNHG1 | FBXO31 | ATP6V0E2 | FBXL7 | PIGY | P2RY12 | EIF3K | EFNB1 | CLPP | SAGE1 | VAPB |
| BRF1 | COPS2 | RCAN3 | GSTO1 | ST6GALNAC2 | GTPBP2 | DERL2 | HOXB6 | NUDT21 | SMC5 | HOOK2 | WDR83 | ZNF207 | ISY1 | KRR1 | DDX27 | RRS1 | KCMF1 | EPS15 | PIM3 |
| EXOSC1 | MTF1 | GPR132 | SMG1 | MIRLET7E | MIR451A | GLYR1 | ZNF2 | EGLN2 | NUCB1 | PDS5B | SUMF1 | MEGF8 | AK3 | SNHG5 | CAMK2A | KIF21B | MEIS2 | ITGA7 | SLC52A3 |
| COQ8A | EXOSC4 | MYOF | EMD | ZNF7 | KIR3DX1 | MICAL2 | AHDC1 | EIF3B | RNH1 | TM9SF3 | LARP4B | GRIN1 | MIR29B2 | TRPM8 | STX5 | HAT1 | ZNF408 | DCLRE1B | MIR324 |
| MSX2 | STX8 | DHRS7B | SLC22A8 | HOXA7 | MATN2 | RGS3 | SEMA3G | SNRNP40 | TMEM9 | TRAPPC2L | CIAO2B | PGGT1B | SSBP2 | TIMM13 | FAM234B | USP38 | MS4A4A | ACER3 | COPG1 |
| BAZ2A | M6PR | AKTIP | BOK | PIP5K1A | UCP3 | PRKAR2B | EIF3L | TBCB | NISCH | CPE | SRR | GPX4 | XRN2 | OSBPL8 | SPSB1 | EDN3 | KIF1A | RIOX1 | GADD45B |
| CRIPT | PNPT1 | SUPT5H | AGFG1 | GBP4 | CHRDL1 | HNRNPUL1 | CDH11 | ZMYND8 | AK4 | NLRP5 | GNG2 | USF1 | CEP68 | CHD9 | FAM172A | DOP1B | TRPM1 | SLCO2A1 | SEMA3D |
| PHIP | RETREG1 | EREG | NKX2-3 | TRIM15 | LYNX1 | WDR41 | TPH1 | MLST8 | PPARGC1B | SLC6A6 | STK3 | KAT8 | HHIP | SKIL | KCNH4 | NPAS3 | AFAP1L2 | HGS | PHLPP2 |
| DCBLD2 | UPF2 | PPP1R14C | HMGN2 | KLHL14 | MFSD1 | AKIP1 | PHLDA1 | PRPF18 | DUSP11 | TMEM163 | C2orf88 | MARCHF4 | SLC25A6 | TMX1 | CLDN8 | POLR2L | NANS | B3GNT5 | GCNT3 |
| RP9 | METTL14 | MLLT3 | CRLF3 | BLVRA | CAPRIN1 | PELP1 | NOP16 | SCRIB | SERBP1 | SLAIN2 | COL4A1 | TNFAIP1 | DPP9 | H2AC25 | TRIM2 | H4C1 | ABCD4 | LOC119230225 | PRPF4 |
| RRM1 | COPS3 | DEFB115 | PRORP | CORIN | IFT172 | STOX1 | SERPINF1 | SNRPA | CNKSR1 | SLFN12L | KLC1 | SRP14 | SOX1 | DGAT1 | COPS8 | SEL1L | AHNAK2 | PIGL | NLRP11 |
| PEX2 | RCN1 | CYP2C9 | PDX1 | ACADSB | TMEM79 | DHX57 | CRTC1 | HRH3 | INPP5K | TFPI2 | UPP1 | SECISBP2 | ADAMTS6 | HOXB7 | KCNMB4 | AP4M1 | APBA3 | EVI5 | BCKDK |
| LRRTM2 | TMEM67 | RPGRIP1 | SLIT2 | DUOXA2 | FIGN | GAREM1 | GK5 | IPP | PIP4P1 | PRDM8 | SYTL1 | RGCC | MFSD2B | NMRK1 | PALS2 | TMEM100 | USP54 | MYO1D | RNF139 |
| NAA16 | POLE4 | ABTB1 | NKX3-2 | ZNF28 | PPM1N | NWD2 | SERTAD3 | TRIM73 | FAM72D | FGF9 | TNKS2 | DAB1 | CYTH2 | ANKHD1 | EXT1 | GJB3 | TCTN3 | CGRRF1 | GIN1 |
| CYP4F22 | SYCE2 | TMC7 | PRDM11 | PPFIA2 | ZNHIT1 | VCX | LPXN | IBSP | GNG5 | COMMD1 | HNRNPA3 | SYNCRIP | DNAJC10 | MYBPHL | HNRNPH2 | SESTD1 | MYH1 | IFT122 | WASHC5 |
| LINC00426 | RSPH3 | SRY | MAPK8IP3 | CLIP1 | CTHRC1 | GID4 | EHF | SLC25A22 | FKBP6 | KIF12 | CRIP1 | IRF2BPL | PTBP1 | DDX3Y | CCNG1 | GRB10 | PKN1 | MYH4 | PPP1R12B |
| CEP85L | CLEC11A | GDI2 | OSBPL1A | MTM1 | ORC6 | DNAJC30 | ARCN1 | MYL3 | SAR1B | PBX2 | EPHA5 | P2RY2 | PPM1G | EIF6 | DDX24 | UTP18 | SMARCD2 | G6PC2 | VMA21 |
| SCN1A | PRAM1 | TCF21 | RNF14 | STK24 | CHMP2B | UEVLD | RNF148 | TCAIM | DNAJB1 | CSAD | PRAME | SAE1 | TNFAIP8 | GTPBP1 | MAP4K2 | NRIP1 | GALNT2 | POLL | RAD1 |
| DHX30 | TLN2 | HPN | SERPINB5 | PAK3 | RPS6KA2 | NUP42 | ENTPD7 | SEPTIN10 | USP36 | IGLON5 | TBATA | PMEPA1 | MIR198 | DPYSL5 | TMEM107 | FKBP8 | EEF1D | RPS12 | BCL2L2 |
| PAX9 | TMEM259 | NEFM | TSN | SMAD1 | CAPN2 | DAAM1 | SAFB | AQP9 | C1GALT1 | RPN1 | YIPF5 | FAM3A | PRR7 | NOTCH4 | CDK13 | MIR99B | SAMSN1 | NPR1 | GDF5 |
| RANGAP1 | MAP2K5 | PPP1R14A | TMEM201 | ALDH3B1 | SSBP1 | SEPTIN1 | GDF9 | POM121 | NR0B2 | MAP1B | PDCD11 | NDUFB10 | PLXDC2 | ZNF622 | SUCLA2 | PSMG1 | CD2-LCR | CHPF2 | CALML6 |
| RPA3 | SGO1 | PPAN | MID1IP1 | CHD2 | APEX2 | ATCAY | FEM1A | TPSG1 | ZNF93 | WFDC3 | POR | BTF3 | DDX54 | GRWD1 | PHRF1 | GCSAM | TET3 | TP53COR1 | SEPTIN4 |
| SCAP | SCN4B | ZNF569 | CCDC137 | INHBE | WNT9B | DCAF7 | PLXNB1 | UBE2O | KLHL25 | PTPMT1 | MC3R | GRHL2 | LAP3 | SRSF3 | TBRG1 | PLA2G12A | ILRUN | SPPL3 | PDGFC |
| CLDN20 | COQ6 | VTI1B | SLC35A3 | FIBP | C6orf15 | H4C16 | MAT2A | PALLD | NAT10 | LRRC59 | AGO1 | RPP40 | ADAM12 | GPX7 | NTN4 | DTX1 | GPRC6A | CDK7 | SELENOF |
| RALGAPA1 | GNG7 | PACSIN2 | SYTL3 | WASHC1 | GEM | GALP | WFDC12 | IRAK1BP1 | MIR136 | CDKN2B-AS1 | MKRN1 | KBTBD13 | SELENBP1 | GPR15LG | UNC5B | POLR2H | BPIFB2 | HDAC8 | EIF4H |
| GATD3 | RNF13 | KIAA0586 | SULT1A3 | SLC22A5 | PHKG2 | PHKA2 | MLX | ABCA12 | ADAMTSL2 | NME8 | CLIP2 | HACD1 | HSD3B7 | FUZ | LIPN | LMF1 | DNAAF3 | SLCO1B1 | DNAJC18 |
| CCDC65 | MRPL15 | GCN1 | KRT13 | MT-TQ | MT-TS2 | KMO | ABCF1 | SUPT16H | DDX47 | GRAMD1A | CD164 | GCH1 | P2RY1 | HSD17B12 | EXOSC8 | TBXA2R | PDZK1 | TGDS | CCDC103 |
| SSX3 | MGME1 | ODAD1 | MT-TS1 | TRPC4 | SYPL2 | GNB5 | GNG3 | POLG2 | FHIT | RAPGEF2 | FPR3 | PNN | MIR181B1 | BMP15 | CMC4 | ADAMTS4 | KEL | MAMLD1 | CPAMD8 |
| MTPAP | DCANP1 | LOC109286563 | AK8 | CDX2 | CENPJ | FAM13A | ETFDH | CHMP4B | P4HA1 | B3GNT3 | EIF2S3 | BASP1 | WFDC11 | WFDC10B | WFDC13 | WFDC9 | WFDC10A | CAVIN4 | ETV4 |
| SLC44A2 | TSHB | TMEM127 | TECR | RERG | NT5C3A | TCF20 | SEMA6A | LGI1 | H1-1 | AGO4 | DFFA | BCAS3 | MEPE | LPAR2 | HOXA1 | CYTL1 | USP9X | PPP2R5E | MIR18B |
| AAVS1 | ASB8 | FBXO44 | FKBP4 | MMP10 | SSNA1 | MIB1 | TXNRD1 | CLPTM1 | RPL27A | AFF4 | MIA3 | FAM120A | OGFR | NOC4L | WDR18 | THEM6 | ILVBL | TRIM23 | CLDN23 |
| DIP2A | LY6G6F | SSX1 | CDC40 | ZNRD2 | FXN | FOXR1 | ALG14 | HBD | NEB | A4GALT | PARP4 | ZNF350 | BICRA | SMPD4 | SLC8B1 | DPY30 | GTF3A | GDAP1 | OPA1 |
| UPF3B | HAUS6 | FBXW4 | UBA3 | MS4A3 | KLRF1 | DCAF15 | FGF22 | LYG2 | TMIGD2 | MFN1 | EIF3F | PLOD3 | GLIPR2 | FASTKD5 | PLOD2 | GSPT1 | SURF6 | E2F4 | GLS2 |
| ANP32A | PCMT1 | ADGRL4 | MLLT6 | SHANK2 | CTBS | GFER | ATP12A | EXOC1 | ATP2A1 | CSMD2 | ADGRA1 | FGD2 | CASK | DYNC2LI1 | C2orf69 | KLF5 | NR5A2 | GMFG | DLD |
| CBFA2T3 | NCLN | SUCNR1 | PIK3R6 | MYCT1 | BAG2 | NAP1L4 | MYL11 | RGS6 | EIF3I | DERL1 | PANK4 | SIM2 | VPS26C | ATP6V1B1 | GOLGA7 | PIGO | MYAS1 | E2F3 | PAX3 |
| AGO3 | NMUR1 | ZNF148 | GFUS | PARP2 | ZNF335 | TMEM219 | ERP44 | RNF220 | RNF4 | ASB13 | FBXW5 | VAT1 | GLP1R | TCERG1 | PDGFD | PDF | PER1 | DUSP7 | SYTL2 |
| MPHOSPH9 | RPP14 | RPP21 | FAM221B | IRS4 | CSE1L | SCAMP3 | ACTBL2 | PDGFA | RPL13A | HSPA12A | UGT1A6 | AAK1 | RPL3 | EIF3E | ACOT8 | PROKR2 | ADD1 | H3C2 | PPIL2 |
| BRD9 | HSPA14 | EFNA1 | SMC4 | KNOP1 | SULT2A1 | RAB1B | FAAP24 | KCNQ1OT1 | RTN1 | CNTNAP2 | HSPA1L | ENDOU | FCRLB | LLPH | CTAG2 | SPINT1 | CCHCR1 | WFDC5 | KCNQ2 |
| IGFALS | SGSM1 | ADPRH | PCID2 | CGB7 | CFAP74 | EXOC4 | RCN2 | AHRR | TNIP3 | JARID2 | SSX4 | GATAD1 | CANT1 | VPS51 | SYCP3 | LOC111365141 | KPNA6 | TRIB1 | TIMM50 |
| WWP1 | TLK1 | UIMC1 | PLPPR3 | ATP5F1A | CYTH1 | MYL6 | DLX4 | MMP16 | BRCC3 | SOST | RRP12 | TMEM131 | POU2AF2 | GPR42 | MAX | PYCR1 | CGN | PTPRB | ATP8A1 |
| DNAJB6 | CPNE1 | TMEM63A | ASB6 | MRPS26 | HELZ2 | SEMA3F | KSR1 | SUN1 | FAM83D | IKZF5 | LINC00261 | MIR425 | CENPV | ANKRD13A | LCT | FNBP1 | TMEM37 | DNAAF6 | METTL7A |
| ADCK1 | NPLOC4 | QARS1 | DUX4 | LRRC75B | SCHLAP1 | HSPA4L | RRP1 | UGT1A8 | EGLN1 | RUVBL2 | RUVBL1 | LSM14A | CRELD1 | LBH | IFITM3P5 | IDH3B | BBS4 | CRLS1 | NRBP1 |
| PIGP | RAB35 | ACCS | CAMK2N1 | GSTA4 | ECD | ESYT1 | COPE | HOXC13 | NCOA4 | NOXA1 | FCGBP | RALY | MLLT1 | PKNOX1 | WDR62 | MIR10B | ZBTB38 | ARX | RPIA |
| MIA2 | C21orf91 | CA1 | EEF1B2 | LGI2 | ZNF74 | DGCR6L | PLA2G12B | TIAM1 | RCC1 | UCP1 | EMSY | GNL2 | TPPP3 | RPF2 | WDR3 | PUM3 | HSD17B8 | SLC12A1 | TUB |
| CDC27 | SMO | NDUFS3 | TBX5 | LONRF1 | DKK2 | LUM | DUSP16 | RGS13 | EPDR1 | FASTK | SVEP1 | NLE1 | SAA3P | PDLIM7 | EFS | COL14A1 | DOK4 | NDST2 | GGT3P |
| CLDN22 | LRR1 | ASB3 | FBXL12 | HNRNPA0 | RBM39 | LIMCH1 | CKM | AKR1C4 | ATXN2L | RPS6KA4 | CCKBR | TAFA2 | TRAF3IP1 | ZPR1 | EVC | RHAG | SIX3 | CLDN15 | CLDN17 |
| RCC2 | EIF2B1 | KRIT1 | OS9 | ERV3-1 | SIKE1 | DAP | HERPUD1 | DDX56 | EIF4G3 | PPP3R2 | TTC26 | GAP43 | PAPPA | SEC24B | SNAI2 | RARRES1 | URI1 | SEMA6B | MIR98 |
| GIPC1 | RRP1B | URB1 | GNA13 | TP73-AS1 | MIR346 | KDM4A | DNAJB2 | RANBP9 | DIMT1 | RNF170 | LTV1 | UQCRB | SLC52A2 | ULK2 | HBB-LCR | SLC25A32 | RAMP3 | MCTS1 | TAOK1 |
| WNT3A | ARHGAP24 | HOXC6 | NUDT15 | CUEDC2 | KNL1 | WDR81 | RAB15 | PAX8 | CTNS | INPP5E | CEP43 | AP3M1 | MIR373 | FAM20A | PPM1A | ARID5B | HSD17B3 | LGALS13 | MAP3K11 |
| HYCC1 | MSTO1 | PDPR | AMELX | SLC31A1 | DPP3 | RPRD1B | SMAP1 | GIMAP1-GIMAP5 | MYRIP | H2BC12 | KCNH2 | PNPLA3 | NOL6 | RRP8 | MIR320A | DNAJA3 | STX12 | KCNE1 | KCNE2 |
| COQ2 | PNPLA5 | ZNF629 | SLC4A4 | GNGT1 | GNG11 | GNG13 | GNG8 | FLOT2 | ERBIN | SSBP3 | LRCH3 | GVINP1 | ANAPC4 | ATP5F1B | SEPTIN11 | PREX1 | ACTR3B | BACE1-AS | OTX2 |
| RPP38 | NXF1 | PPP1R13B | CR1L | TAAR9 | DNM1 | CDA | KLC2 | UBE2G1 | RAB3D | BRK1 | ASB9 | HERC3 | LRRC41 | KIF26A | SPSB2 | MUCL1 | BTBD6 | NKIRAS2 | NKIRAS1 |
| COPG2 | MORC1 | POP5 | RPP25 | STIL | DCDC2 | MPHOSPH10 | EFNA5 | POLR2C | KRT20 | RHOU | KPNA5 | BOLA2 | CHD5 | DNAJA1 | P2RX3 | COIL | MIR663A | HPS4 | PTPRH |
| CHRM1 | KRI1 | ATP6V1G2 | CPSF3 | ZFAT-AS1 | MUTYH | NPR3 | SYNE2 | IFRD1 | TIMM44 | ZFR | COPS4 | FCF1 | MAP7D1 | NCBP3 | ST2 | DLST | SLC25A11 | RAB27B | NCF4-AS1 |
| GNA12 | USP25 | ADAM9 | PTK7 | RPL7L1 | SESN3 | USH1C | GCM2 | MIR3667HG | XAGE1B | HCN2 | GPR17 | MPZL2 | ST3GAL6 | PSD | CECR2 | SNX15 | TTC8 | EPHA1 | ABCC5 |
| TST | CCT7 | SRPRB | WDR6 | MYOG | SUMO2 | PRINS | MIR506 | NARS2 | NEFH | CYP26A1 | EIF2B3 | NOLC1 | SMPD2 | TOR1AIP1 | LCLAT1 | CCDC85B | MRPL40 | MLLT11 | POMT1 |
| FZD1 | HOXB9 | DCLRE1A | PAXX | DIPK2B | TCTN2 | TMEM231 | GIT2 | MAP1A | AHCYL1 | RBBP7 | NCAPG | CEP170 | SAMM50 | NCBP1 | SDAD1 | UBL4A | CREM | LONRF2 | RADX |
| MRPS25 | SOSTDC1 | NPRL3 | ESPN | SMOC1 | ZMYM2 | VKORC1 | ASCL1 | THSD7A | SIK1 | SLC25A24 | TRIO | SMARCD1 | NHLRC1 | MAB21L1 | ZNHIT3 | ZNF384 | GMDS | SLC7A6 | SSTR3 |
| CTH | MRPS2 | SDF2 | NPR2 | DNAJB12 | SRP9 | STAB1 | CCKAR | ALDH9A1 | MC5R | PKP4 | CDK11A | COG3 | STAC | TTN-AS1 | HBE1 | SLC37A1 | ZBTB21 | FUT1 | SCIN |
| PHETA1 | AIMP2 | CLGN | SDF2L1 | LINC00824 | LINC01882 | LINC02352 | CUTALP | DUTP6 | ENSG00000234630 | RNU6-1133P | RNU6-959P | RNA5SP185 | RNU6-1149P | RPL13AP23 | ENSG00000287218 | RNU6-376P | H3P5 | CCDC69 | C2CD2 |
| RF00017-1277 | PI4KAP2 | ADAM1A | LINC01122 | ENSG00000289970 | HSALNG0013961 | HSALNG0045887 | HSALNG0150714 | RF00017-5051 | RF00026-586 | RF00026-648 | lnc-BRAP-1 | lnc-PSMD5-5 | lnc-YDJC-1 | piR-43105-423 | lnc-CEP76-2 | piR-61240-151 | HSALNG0091647 | RN7SKP226 | PSMC1P4 |
| TOX | lnc-ANXA6-3 | lnc-CD247-1 | lnc-RCSD1-5 | PTPN18 | EIF4E3 | FUCA2 | HTR1B | MRPS27 | RAI14 | MRPS5 | C7orf50 | SIL1 | SNRPD2 | H3-3A | HAGH | LAD1 | TLE3 | RF00994-258 | piR-40398 |
| SLC9A1 | CCDC115 | LAMTOR5 | PITPNA | MAFG | RNASE10 | RNU6ATAC | CCNE2 | CHP1 | DAP3 | TRAPPC3 | GEMIN4 | SACM1L | TMX3 | APOBEC2 | NOB1 | TNKS1BP1 | PALD1 | RPL24 | RNF186 |
| PSG3 | AKAP8L | PRRC2C | BDKRB1 | PSG8 | PSG7 | SHLD1 | GGT2P | EXOSC5 | EYS | PGAP3 | NPL | SCYL2 | EPYC | HELB | NRG2 | ATP2C1 | MT-TK | TIAL1 | MARCHF6 |
| NDUFB4 | PSG6 | TTLL1 | THEMIS2 | URGCP | VPS8 | BHLHE23 | BACE1 | POMGNT2 | TINAGL1 | IGSF21 | TAFA1 | TAFA5 | TAFA4 | ZNF684 | TAFA3 | IGSF23 | SSX7 | LTBP2 | SLC22A12 |
| SSX6P | SERPINA12 | MORF4L2 | TMEM199 | LOC116158494 | LOC116158495 | RAB3GAP1 | SGCD | TNNC1 | CYP26C1 | TCAP | CSRP3 | PPCS | HSCB | RBM20 | UBE2S | MGST1 | SGCA | SSX4B | SSX2B |
| ARSL | SSX9P | IGHV3OR16-9 | TRGJ2 | NUBP1 | KRT23 | MIR199A2 | EGR2 | PTGIS | MAP3K10 | MYO6 | GIGYF2 | ZNF318 | CCDC102A | HCG25 | MIR4774 | MIR4804 | DGAT2 | ARID3B | CDC42BPB |
| ALDH8A1 | GPX5 | PRND | TMED10 | IRF2BP1 | LARP4 | SPCS2 | MAIP1 | ADAMTSL3 | GRM7 | ABCC6 | GALM | MAP3K20 | UGP2 | XPNPEP3 | CIC | NBEA | TSEN2 | SLC16A4 | VAMP4 |
| ARHGAP29 | ATF5 | EHD2 | SART3 | CFAP221 | VPS37D | PWAR1 | LOC110806262 | LINC00111 | IGHVII-65-1 | IGHVIII-67-4 | CNN2P11 | IGKV2-36 | ENSG00000233712 | ENSG00000275654 | FDFT1 | S100A6 | HNRNPR | COX8A | TBX4 |
| CDR2L | TTC12 | NEPRO | KATNIP | TMPRSS12 | OVCH1 | PTPA | CCN4 | IFT43 | CRHBP | CYB5A | PNPLA6 | TRHR | IGHMBP2 | ADPGK | COQ3 | MITD1 | TBC1D30 | MAFF | SLC22A2 |
| STC1 | THBS2 | MIR500A | RNF123 | MIR557 | AZIN1 | CD200R1L | GRK6 | ADCY9 | NQO2 | KIF13A | SACS | ALDH1B1 | HS2ST1 | EXOC5 | TRIOBP | ANKRD28 | BET1 | LINC00467 | PRRT2 |
| MRPS9 | MARCKS | GINS4 | SI | STRBP | TAS2R14 | SCFD2 | TMEM255A | TRMT61B | DMAC2 | IKBKB-DT | LOC102723407 | EEF1G | SF3B3 | UBAP2L | DHDH | TOPORS | PIAS2 | EHD4 | MRPS23 |
| CSMD1 | SRRM1 | BRIX1 | MLF2 | ZNF366 | RAD51B | LTK | RSF1 | TCL6 | SEC24D | CAB39 | KLHL41 | BIN2 | PDZD11 | FBP1 | ARL1 | BMAL2 | GRAMD4 | KCNRG | MIR154 |
| VENTX | CHRNA5 | CYP4F3 | CD302 | UBAC1 | FBXO22 | DEFB129 | KCNA2 | GYPA | LINC01194 | RPS18 | THRAP3 | DEFT1P | GPD2 | CDC25B | UQCRC1 | ETF1 | EFNB3 | ATF7-NPFF | CCPG1 |
| CHMP5 | CYP2R1 | SMAD6 | AP1S2 | MRPS17 | MRPS18B | DDX28 | MRPS34 | MAP1LC3C | UCN3 | AP4B1-AS1 | NPY1R | DAD1 | BAG4 | H2BC4 | H2BC7 | CAMLG | SET | PLCD3 | PLCD4 |
| KCNK3 | MRPS7 | PLA2G4B | LRCH4 | SLC30A6 | GLB1L | ARHGAP23 | MPP2 | RFC3 | EGFL7 | MAZ | HEATR1 | PRSS8 | COL9A3 | SGCB | DSPP | SLC30A4 | KLK8 | NKX2-6 | ZFYVE19 |
| LRRK1 | FLRT3 | INSIG2 | SLC9A3R2 | ST18 | ZC3HC1 | ARHGEF26 | TMEM204 | SLC22A25 | SLC51B | CYS1 | MSMP | CCDC154 | USP41 | CS | LPAR1 | CCAR1 | MIR199B | WDFY3 | DPH2 |
| UQCRC2 | SYT7 | ANXA9 | HSD17B11 | RPL6 | RPL17 | THOC7 | DYRK1B | RSPO1 | SMS | FMO5 | WNT6 | CLCNKA | PAPPA2 | RGS12 | USP16 | ASH1L | PPM1M | EGILA | CILP |
| RIPPLY3 | PEX14 | GNAZ | PFN2 | CDC23 | KIF4A | RCHY1 | ANAPC5 | RNF144B | HEBP2 | ACVR1 | PPIB | PNPLA8 | CAMTA1 | AMT | HSD17B2 | APLP2 | ASCC1 | SSPN | GPR89B |
| VPS4B | ZNF595 | CYP2B6 | DIS3L2 | MRPS35 | GDE1 | PURB | KCTD10 | MRPS31 | MRPS15 | LNCATV | SIRPAP1 | SIRPB3P | KLRF2 | SNU13 | SRSF11 | NOVA1 | RRP15 | UGT1A4 | CYP3A7 |
| CYP26B1 | MRPS28 | NUBP2 | RGS17 | HNRNPAB | SUMO3 | BTC | CSNK1G1 | BMP8B | MEF2C-AS2 | KIF1B | NEDD8 | EMC1 | RPL36 | ETV1 | TGFB1I1 | SNHG29 | PRKAG1 | C15orf48 | F2RL3 |
| LAMB1 | RAB2A | ASIP | ABCF2 | RFC5 | PELO | GPRIN1 | COMMD4 | INVS | DNAJC6 | PIH1D2 | RNF103 | ERVFC1 | ERVFC1-1 | ERVPABLB-1 | ERVS71-1 | NPTN | MYO5C | MARK2 | TSPYL1 |
| CAMKK1 | PDIA4 | AATF | DNAJC7 | MIR203B | TRPV5 | TBC1D4 | COL9A1 | ADAMTS9 | CD248 | PTCRA | AGBL2 | H2AZ2 | ANAPC7 | TXNDC5 | RBBP6 | A1BG | UBE3D | CLINT1 | DUSP19 |
| FZD7 | PTPN23 | KPNA7 | PTPN20 | DIO1 | HUS1 | MT3 | SCGB2A1 | ZNF879 | CBX1 | ARFRP1 | SLC7A8 | DNAJA2 | MYL1 | CACNB2 | RPL4 | RPL14 | HYAL2 | FABP4 | UCA1 |
| CMTR1 | ATP1B2 | CBR3 | MBD1 | TXNL4A | DCTD | WSCD1 | ABCA4 | MEF2D | ADCY1 | SSTR4 | GDF11 | ASTE1 | PAGR1 | NDUFS1 | SPRY1 | CSDE1 | DDX10 | RAP1GDS1 | RPL12 |
| LARS1 | TMEM41B | GKN2 | WNT2 | ORC2 | MIR4273 | PDYN | GBF1 | ROMO1 | PASD1 | CHRNB1 | CHRND | ATXN10 | SLC12A8 | CENPL | UTS2 | AIFM1 | PTPRS | POLDIP3 | SPAG5 |
| ALDOB | SNW1 | SF3A1 | ST13P4 | RBM25 | ZBTB14 | HTR1D | ABCB6 | ABCA5 | SOAT2 | ALDH1L1 | SCUBE2 | ABCA6 | SAT2 | PPP1R11 | RMDN2 | RPA4 | ZNF230 | LHX1 | PDZD7 |
| CHROMR | PRPH2 | MIR582 | USP6 | NID1 | HADHB | SRSF9 | MAP4 | CWC27 | ABCB9 | DEFB112 | PYDC5 | CTSF | CCNF | CUL7 | MYO10 | AP1M2 | NPDC1 | CCDC110 | SPINK4 |
| DCTN6 | CFAP58-DT | RCVRN | PSORS1C3 | GHDC | C1orf35 | FBXO30 | ASB17 | ATP1B3 | MINPP1 | AGXT | NFS1 | SLC1A4 | SLC6A5 | SULT2B1 | SLC6A19 | PHKB | CIDEC | RNF126 | RNF138 |
| TSEN15 | DCTN3 | KLHL11 | RILP | TBX19 | TSEN54 | UROC1 | SDR9C7 | CFAP298 | CLTRN | NADK2 | FBXO28 | MAGEL2 | PPP1R21 | PRDM12 | PYROXD1 | DNAH1 | DNAJB13 | REEP1 | RIN2 |
| CFAP45 | DDX59 | GPR101 | NAXD | METTL27 | PET117 | NPAP1 | TMEM270 | PWRN1 | IPW | NOTCH2NLC | SNORD115-1 | SNORD116-1 | MKRN3-AS1 | PPP1R9B | NOP58 | DBP | SMU1 | DZIP1L | SMG8 |
| LSM12 | CFAP52 | ODAD4 | GTF2IRD2 | NR5A1 | UNC13B | TJP3 | CCNT2 | ENPEP | RPL22 | SBNO1 | USP49 | KRT72 | ECSCR | MIDEAS | USP17L3 | USP17L1 | USP17L4 | USP39 | RPL36A |
| FKBP14 | TRMT1L | KIR2DP1 | RILPL2 | EIF2B2 | FAR1 | RPL23 | SCFD1 | GEMIN5 | GPS1 | CENPA | FOXA2 | SLC2A9 | MMP15 | PDK4 | CA6 | PAX7 | SCTR | USP17L7 | USP17L8 |
| CAPN10 | PPID | CACNA1F | RBL2 | PLSCR3 | DMXL2 | LRCH1 | EDDM3B | BCL9 | STEAP2 | EHBP1 | KHDRBS3 | SPAG8 | STMP1 | TMPRSS9 | ACSL4 | CEP120 | CEP19 | SLC9A2 | ANKH |
| SLC14A1 | NALCN | PARL | B4GALNT2 | RNF146 | SUGP1 | RORB | RFC4 | NCAPH | PLIN3 | CIAO1 | OLIG3 | BOP1 | TTC21A | ITPRID2 | UBN2 | MYH13 | DIS3L | COL4A2 | GPLD1 |
| KRTAP5-7 | FMNL1 | GSTM4 | MKRN2 | SEC24A | ACTR10 | SEC61A2 | LOC102725035 | LOC107987462 | LRRC2 | RNF44 | SYCP2L | LRCH2 | SLC25A5 | ESM1 | FUBP3 | CHGB | SLC34A1 | BOD1L1 | ESX1 |
| RFX4 | DLEU2 | GPC1 | ANK2 | RBM17 | SIPA1L1 | LGALS16 | SNRPB2 | MLN | PYGB | SEC31A | UBA5 | ARHGAP9 | CDC16 | POLR2F | RAB5B | CREG1 | KLHL9 | ZP4 | LRPAP1 |
| RNF130 | PCSK2 | DDX20 | MAGED2 | RNF25 | KIF18A | PPP2R5B | TMBIM1 | WIPF2 | BTNL9 | FBXO2 | KLC4 | MUC17 | POLR2K | FBXO21 | FBXL14 | UNKL | MUC21 | RBSN | RNF111 |
| FABP6 | UBA6 | KLHL20 | RAB37 | PTGFR | RABGEF1 | GPA33 | TRIP6 | NSMAF | ATP5MK | MBL1P | SLC25A21 | NID2 | USP28 | SLC28A3 | ALKBH1 | EBF2 | USP12 | GET3 | SEC16A |
| DHRS4L1 | ZNF740 | ZNF512 | ANTXR1 | C22orf39 | NR1I3 | KLK14 | OSGIN2 | GRB14 | RGL1 | HSD17B10 | DPYSL2 | ETFA | ATP2B1 | DECR1 | MDN1 | SNRPF | FOXK1 | ZFYVE1 | DHRS4L2 |
| PRNCR1 | GLRX | TENM1 | HOXD13 | MYBBP1A | PDCD6 | METTL9 | PTCH2 | PEX1 | COX6B1 | LGALS2 | ITSN1 | SLBP | HS6ST1 | NSMF | NIPA2 | SLC46A3 | HEBP1 | GAR1 | HOTTIP |
| TUT1 | ARMS2 | KDM5B | PTGES3 | PUS1 | DHX16 | TRAPPC4 | EIF3J | BEND3 | SYNC | RFLNB | MCM8 | RHBDL2 | CLUAP1 | BECN2 | RNA5SP141 | GSTT1-AS1 | BVES | MIR339 | CEACAM4 |
| MBTPS1 | RNU5A-1 | CES1 | SOX3 | BCO2 | ZNF281 | SELENOW | SIGLEC16 | PPP1R12C | LEFTY2 | SERPINF2 | ADH1B | MIR196A2 | ATP13A1 | EIF3G | RPL37A | EMC2 | NOP9 | PRKAG2 | ENTPD5 |
| CDK8 | GRAP | ESPL1 | ARID3A | HCAR3 | ACRV1 | DEFA1A3 | DAG1 | CYP27A1 | KIF22 | ATP6V0A4 | DNAJC5 | PPIE | ANAPC10 | ATP6V0C | NAPRT | TRIM71 | LAMTOR3 | SLC35G2 | STIMATE |
| RNF19A | TPM4 | TTF2 | SRRM2 | DYNC1LI2 | FBXL3 | ANAPC13 | KBTBD7 | KIF2B | KIF3C | TSPAN14 | ARL8A | ATP6V0B | ATP8B4 | CPPED1 | FBXO15 | FBXO40 | MUC15 | RAB31 | ATP6V1G1 |
| FBXL16 | UBE2Z | ABCA13 | DERA | FBXL22 | FBXL8 | CDC26 | FBXL15 | ASB12 | FBXO27 | FBXO41 | SPSB4 | TMED7-TICAM2 | CELA3B | CELA1 | WASHC3 | WASH3P | RNY1 | TUBAL3 | FBXL13 |
| SLC2A2 | KLHL42 | ENPP4 | STBD1 | STON1 | EFCAB11 | AKR1B1 | EED | SASH1 | PPP2R2A | ABLIM1 | DOCK10 | NELFE | GAB1 | MYH7B | MPHOSPH8 | ZC4H2 | MACO1 | LINC01193 | ADH7 |
| HS3ST4 | DDIT4 | NOL10 | UTP3 | PTDSS1 | STAMBP | TRIM33 | NMT1 | CDH17 | ADAM28 | CAPG | UVRAG | CBX4 | OXSR1 | SLC4A10 | TAF4 | BCAR3 | MRPL12 | H2AC6 | PCAT1 |
| SMG6 | PTGIR | CLCN3 | P2RX2 | IFI27L1 | POU3F4 | DGCR11 | TIMELESS | GIP | EPB41L1 | GOLGB1 | LPAR6 | ARL2BP | WEE1 | EIF5B | SFXN1 | TRPV3 | CRX | TRA2A | CNOT4 |
| LYL1 | GRSF1 | PTCHD1 | NAA35 | LIPE | SYNGAP1 | CPM | CLPX | EAPP | CIAO3 | ZNRF4 | IQANK1 | ENSG00000250264 | ENOPH1 | BABAM2 | CENPN | NGDN | PPAT | MLF1 | FASTKD2 |
| XPO7 | FAM167A | RYK | DKKL1 | HOXB2 | H2BC11 | KAT2A | ASNS | RFC1 | FDPS | NSF | CYGB | NEU3 | CNNM3 | REC8 | THOC1 | H2BC3 | EEF1A1P5 | SFRP2 | UGCG |
| ADAMTS1 | CRNDE | MIR96 | SLC28A1 | DUSP5 | ELP3 | MBD3 | ME1 | NEK7 | MFNG | MTMR4 | NCAM2 | NCEH1 | PRMT3 | RAB6B | ARHGAP10 | AGTRAP | CNTN5 | IGHV3-69-1 | LOC107980440 |
| RRAGA | DAGLA | ATP10A | CLASP1 | ERRFI1 | GABPB2 | GPRC5C | IFITM5 | PPP1R16B | WFDC1 | ADAM7 | ZBTB42 | EPGN | KLHDC2 | AIF1L | MMD | WBP1L | C1QL2 | DNAJC11 | REXO2 |
| TMSB4X | SCGN | SUN5 | CD2BP2 | SCN8A | AMHR2 | CACNB4 | KCNA1 | MFAP5 | CARTPT | SGCE | PRSS12 | RAMP1 | PTF1A | SLC39A5 | SPEG | KCNA10 | RABGGTA | MB21D2 | TMEM121B |
| TMEM126A | PKHD1L1 | NFIA | FOXE1 | APOO | CIZ1 | HOXC4 | MYCL | NHLH2 | ODF2 | SLC25A46 | TPSD1 | CLUH | OMA1 | AGBL1 | COL26A1 | UNC80 | FUNDC2 | DNMBP | NAV2 |
| AGBL4 | TNS1 | FGD3 | HS3ST3B1 | AP5M1 | NHLH1 | PMS2P2 | ACADS | BPIFA3 | RNF39 | CLCN5 | MYL4 | PDP1 | FRS2 | FRMD6 | SAMD15 | ARFGAP2 | MIR24-2 | SUCO | ACER1 |
| SIRT5 | FAM163A | LRRC17 | ADNP2 | XPOT | HNRNPH3 | ATP5PB | LUC7L3 | TMED2 | ANP32E | CNOT9 | FAM98B | SH3BGRL2 | SLIRP | YLPM1 | TTC27 | H2AC12 | SAAL1 | CDH15 | NT5C2 |
| MCC | VDAC3 | RPL34 | ATP5F1C | PODNL1 | KLF10 | GRK5 | STATH | POGLUT2 | PHF14 | PNMA3 | LUZP6 | SIRT7 | PLIN1 | MIR365A | CYP2S1 | AP3B2 | PDLIM5 | SH2D4B | PUF60 |
| PBK | MMS | HAAO | STX1B | CLP1 | INHBB | STAG1 | AJUBA | ESAM | KIF13B | HOOK1 | OTUD6B | ESRP2 | PACSIN1 | HAPLN4 | IMMP2L | MYO1F | NAV1 | PDZD2 | BCAT1 |
| SLFN14 | KCNJ8 | MAP4K5 | USP20 | ZFYVE21 | KLHDC10 | LONRF3 | MED19 | TTC28 | CHURC1 | PRMT9 | SMDT1 | KPRP | TEDC1 | YY2 | CYP21A1P | GULOP | ADCY6 | SETD3 | YPEL4 |
| MARK3 | ADM2 | GDPD3 | PTPRCAP | SLC30A9 | STK38L | ACIN1 | ARF4 | BZW2 | CLCC1 | PIGS | RRBP1 | BCAS2 | IPO4 | MMS19 | RAB11FIP1 | LGALS7 | LY6K | GLRX3 | GATB |
| PRR16 | ZYX | SERPINA5 | MCM6 | FAM135A | MS | MGAT5 | HDAC7 | NSFL1C | ORC3 | VPS28 | TCTN1 | ARFGAP3 | PDCL | MTUS2 | KIRREL3 | HOXA2 | DNA2 | MRPL18 | GRAMD1B |
| PDILT | EMC3 | LOC106627981 | EVPL | RNU6-1 | GK | EIF2B4 | FSTL3 | ST13 | ASCL2 | MAGED4B | USH2A | MAPRE2 | KCTD13 | FGF21 | RS1 | STAC2 | BABAM1 | ANOS1 | HBQ1 |
| ART3 | FAM149A | STH | WDR72 | PUM1 | CLTB | LRFN5 | NCAPD3 | REEP5 | VIL1 | BMP2K | CDK3 | PPIL3 | ZW10 | ALG5 | PSRC1 | RANBP10 | SDF4 | TOR4A | SLC8A3 |
| DCAKD | DAPK3 | DEPTOR | MRPL3 | VMP1 | DMAC2L | GID8 | HDHD5 | H1-10 | HEATR3 | GARRE1 | PALM2AKAP2 | DENR | BHLHE22 | RNU11 | SPAG11B | NR6A1 | MACC1 | DUS4L | MRPL4 |
| HESX1 | KCTD12 | RNF11 | SAMD4A | COMMD7 | CT83 | CACNA1D | FH | ELK3 | GYG1 | TUBB1 | ATP6V1E1 | PAFAH1B2 | UBE2R2 | ATP6V1F | DEGS1 | ELMO2 | HECW2 | ETV5 | LHX4 |
| ATP11B | LRPPRC | ECI2 | RNGTT | CPN2 | GAN | KLHL3 | PGM2 | ALDOC | KIFAP3 | KLC3 | PRR5 | SCAMP1 | CTSO | OSTF1 | ATP6V1E2 | DCTN5 | UBE2D4 | RAB3A | SYNGR1 |
| NIT2 | ATP6V1C2 | ATP6V1D | ATP6V1G3 | UBE2Q1 | ASB11 | KBTBD8 | PLPP4 | RAB24 | CAPZA3 | KLHL21 | ASB18 | DET1 | FRMPD3 | KCTD6 | KIF4B | SVIP | TUBA3D | ASB10 | KLHL5 |
| KBTBD6 | RAP2B | RAP2C | SLCO4C1 | CBLL2 | GPR75-ASB3 | MIR378A | UMLILO | AGPS | SLC2A6 | NOTUM | BOLL | SEPTIN3 | HCRTR2 | CALD1 | CTBP2 | EMX2 | LOXL4 | UBE2U | ASB15 |
| COLGALT1 | NHLRC3 | ASB14 | WIPF3 | NDUFB6 | SEC23IP | USF2 | MRPL11 | OLFML3 | MOB1B | MRPL1 | STRAP | ERH | MRPL22 | MRPL43 | MRPL49 | SLC38A9 | YTHDC1 | RCOR1 | CYB5B |
| MOB3B | RBM10 | ATP13A3 | MED17 | ENY2 | FAM8A1 | PCMTD2 | QTRT2 | LACRT | MRPL52 | NUDT22 | BTBD18 | SMN2 | SLC10A2 | SLC4A1AP | CERS2 | RABEP1 | MANEA | CILP2 | IWS1 |
| FNTB | PLEKHG3 | PTDSS2 | SNX6 | KCNT2 | MTSS1 | MYBL1 | NEIL2 | VGF | SGMS2 | STX2 | CUZD1 | B4GAT1 | CCDC25 | CIBAR1 | ELK4 | KRT34 | NFATC2IP | GNL3L | NPM3 |
| CHCHD7 | DHRS4 | PTPRE | CREB3L3 | FAM161B | NEURL4 | SNX31 | CARMIL3 | KRT27 | PPP4R3B | ZC2HC1C | FHIP2B | NTAQ1 | TTC9 | NUGGC | TMEM64 | CCDC166 | CHURC1-FNTB | PGAM4 | PHF20L1 |
| BPIFB3 | CMTM5 | CSRNP3 | EMX1 | NTSR1 | TRPV2 | CARD6 | ANK3 | DIO2 | GPD1 | KCNN3 | TULP3 | KATNB1 | USP53 | RSPRY1 | DLEC1 | ADGRL1 | INS-IGF2 | C8orf44-SGK3 | TRAJ33 |
| NUSAP1 | MMADHC | RFPL3S | MIR188 | CNGA3 | SOX17 | IMPG2 | PEX26 | WHRN | CLRN1 | MFRP | FAM161A | PCARE | PRCD | MLIP | COL12A1 | MYH14 | MIR138-1 | MIR519D | GLE1 |
| CPT1A | RTEL1-TNFRSF6B | ASF1A | SCO1 | PAICS | SMC2 | ZC3H14 | UXT | PPP6R1 | BRCA1P1 | ODF3B | ZNF767P | ENSG00000173366 | MRPS17P5 | CHRNA2 | GDI1 | ITM2B | FNTA | MIR138-2 | MIR187 |
| TIMM8B | FZD5 | PFKP | DGKB | ZNF250 | PRR4 | LRRIQ3 | WFDC8 | ZDHHC22 | FBXO48 | LOC107963955 | SCHIP1 | LINC03053 | PELO-AS1 | SLC5A11 | KRT4 | PDE4DIP | NRXN1 | TMX2 | COQ10B |
| NAGA | NT5DC2 | SCCPDH | TRIM72 | VRK1 | LDHB | UGDH | ELOVL5 | ZFYVE9 | EXOC7 | THOC2 | UBQLN1 | SART1 | EDF1 | IMP3 | STX6 | SF3A3 | SRPRA | LOC117038795 | CHID1 |
| COMMD2 | CENPC | SERPINB7 | POMT2 | CPA4 | SPCS1 | PCGEM1 | DCLK1 | RGS10 | ANGPTL2 | A3GALT2 | TIFAB | C1QTNF5 | INTS9 | POU4F1 | ARMC9 | TPTE2 | DCHS1 | H2AC11 | TMED9 |
| NRL | MIR103A1 | AVPR2 | PDHX | LINC02384 | MT-TG | SERPINA11 | ANKS3 | CDK11B | CADM3 | PPA1 | KCTD17 | KYAT3 | MTF2 | TTF1 | ATG101 | CAPS | CHMP7 | SEPTIN6 | ERMAP |
| MTERF3 | RAB3IL1 | ANKRD49 | CXCR2P1 | CIPC | ERICH5 | C8orf76 | C8orf44 | CHD3 | ADH1A | KCNAB1 | PHF21A | PRPS1L1 | HIC2 | SLC22A23 | PRPSAP2 | U2AF1L4 | MIR34B | LEPROTL1 | FAM120C |
| NDUFB9 | NIPAL2 | TPRN | MAGEA12 | ERAL1 | NCAPD2 | SLCO4A1 | TELO2 | RHOT2 | ATP5PD | DCP1A | NR2F6 | PPP1R18 | SUB1 | THADA | TRMT10C | UQCRH | BZW1 | SLC34A3 | TNK1 |
| YIPF3 | FGD4 | GAK | VPS53 | S100A16 | SLC22A17 | ELFN2 | HNRNPUL2 | SLC35E1 | TBRG4 | ZNF598 | LOC117134593 | APCDD1 | THRA | EEF2K | BYSL | UTP6 | LGALS14 | NEMF | PLP2 |
| SSTR1 | ATP5MF | MEAF6 | RANBP6 | CDK19 | COX4I1 | NDUFS8 | MTA1 | NDUFAF4 | ADGRL2 | BTAF1 | AGPAT4 | LRP1B | ATG10 | NDUFA5 | PTGFRN | FAM20B | IPO11 | GLUD1 | APOC3 |
| WTAP | BNIP3 | P2RY8 | SLC16A1 | LHX2 | ZNF175 | ZNF326 | ARFGEF3 | BET1L | EGFL8 | IPO9 | PNRC1 | COMMD5 | ECPAS | H2AC4 | NUFIP2 | EEF1AKMT3 | LINC00472 | NCAPG2 | USP34 |
| MIR181B2 | ARPC5L | CORO2A | ERP29 | WDR5 | CHRNA4 | DKK3 | TPX2 | ADRM1 | MT1E | MT1X | NSMCE1 | CKAP5 | NOP14 | LGALS17A | SCFV | ALDH6A1 | MAPRE1 | IRF1-AS1 | CSAG2 |
| PRELP | MIR218-1 | CSAG3 | KCNQ1 | MYCBP2 | ANKRD30A | KRT78 | QRICH2 | ZBTB49 | TDRP | HNRNPCL1 | EPOP | LOC102723878 | SLC1A2 | MCHR2 | MIR615 | TPPP2 | MIR662 | ETFB | OVGP1 |
| CACNB1 | SLC28A2 | SNTB1 | DR1 | ADAMTS7 | FDX1 | PI15 | PLK4 | ID4 | HTR7 | SPRY2 | SARS1 | DDAH1 | NUDC | RARS2 | FREM1 | PIGK | POU3F2 | PABPN1 | MAK |
| SF1 | MYO18B | C20orf203 | GRK3 | USP33 | STXBP3 | VTI1A | ELF3 | OBSL1 | CAND2 | ESRP1 | NAPA | MKX | MRPS10 | NXPH4 | SEC62 | TBC1D15 | TMTC2 | EDN2 | GPM6A |
| SPECC1 | TFCP2 | TMEM59 | TPST2 | CPNE8 | LNPK | SNAP47 | SPATS2 | USE1 | BRI3BP | GTPBP10 | RETREG3 | H2AC14 | MRPL45 | PSMG3 | TBC1D9B | TTC36 | HNRNPA1L2 | TUT7 | MED7 |
| CSNK2A3 | STX18 | NMRAL1 | CNOT6L | PROK1 | LHB | DNAH14 | TNR | ATP2C2 | MGAT4B | GPR83 | PARD3 | NFE2 | HOXA9 | MGAT5B | NUB1 | PFDN1 | KLHL6 | C8orf33 | LRRC47 |
| EMG1 | GYPE | MIR103A2 | ABCC3 | ANGPT4 | NDUFAF1 | TOB1 | CDKN2D | MIR758 | H2AC17 | MIR422A | MIR494 | CALB2 | RBM33 | RPL21 | RBMX | BAAT | NUDT1 | SLC43A2 | ZFP91 |
| DCUN1D1 | TBL3 | UTP14A | RRP36 | ZNF503 | OR1E2 | MAP4K4 | CILK1 | IMPA2 | MRPS16 | FTSJ1 | KIF14 | PPFIBP1 | APLP1 | ARFGEF1 | CDIPT | ELAVL2 | KIF21A | NSUN5 | PPP1R8 |
| ATF6B | PLEKHA5 | SLU7 | RSAD1 | CHCHD2 | EIF1 | IPO13 | APOL2 | CEP72 | CHI3L2 | CLN6 | LDB1 | LPGAT1 | NIP7 | PACSIN3 | SLC25A28 | XPO4 | FCHO2 | TRAPPC6B | UBAP1 |
| RAB13 | EHD1 | ING5 | REPS1 | SIVA1 | SSBP4 | TRAPPC5 | ARHGAP11A | MRPS11 | DXO | KLHL26 | MED29 | MON2 | NFXL1 | NUDT19 | PSD4 | TXLNB | VWA8 | GEMIN6 | GYPB |
| MRPL9 | CCDC93 | MED21 | MRPS6 | H2AC7 | PHLDB3 | CRACD | KIAA1671 | TRIM52 | CTAG1A | NEU2 | TMBIM6 | TPD52L2 | MRPL24 | RIPOR2 | TPTE | NUCKS1 | ZDHHC20 | CRACR2B | MAGEB2 |
| KRT18P39 | MYO19 | STX10 | C1orf112 | PAK1IP1 | SKA3 | WDR46 | TEX10 | UTP20 | EPIC1 | SLC12A2 | GJB1 | KCNA5 | CLIC5 | PLXNA2 | STK26 | ASIC2 | RGS2 | KRT80 | MRPL47 |
| RNF40 | RAET1M | DMTN | DDX18 | TKTL1 | C1QTNF6 | EPC1 | ARID4B | RIOX2 | EIF2D | MT1H | RETNLB | ACOXL | CSH2 | NKG7 | IL17RD | TBC1D5 | VANGL2 | BRAP | PTP4A1 |
| POU6F2 | ELL2 | KLF13 | RGS16 | ALDH3A1 | ADGRA2 | HOXA11 | IGLV2-5 | GNAL | UCN2 | VEGFB | PEX10 | OXCT1 | ACAA2 | NDUFA12 | FKBP10 | NDUFA10 | SUCLG2 | MMP21 | HYLS1 |
| LOXL3 | CCDC32 | MYEOV | SEMA5A | GFM2 | GUK1 | NUBPL | PLA1A | TUBGCP4 | ART4 | ATP5PF | DPYSL3 | FIGNL1 | MOB1A | RDH13 | SBF2 | DUSP13 | GTSE1 | ATP5F1E | FHL3 |
| DENND1A | SIRT4 | SMYD3 | ATP5F1D | HIGD1A | IFT74 | MAB21L2 | NAPG | NOM1 | PIR | RMND1 | RMND5A | TMOD1 | TNPO2 | APMAP | ATP5ME | CDKN2AIP | DHX32 | MCM9 | PIGH |
| GTF3C1 | DYNLRB1 | EEFSEC | FAM3C | PXMP2 | RSL24D1 | TACC2 | VAMP5 | ZNF219 | CMTM3 | DCBLD1 | EI24 | ERCC6L | GPKOW | HENMT1 | IFRD2 | ISL2 | KNTC1 | DNAJC1 | FADS3 |
| MRPL50 | LAPTM4A | PLEKHB2 | POLR2G | PLEKHG1 | RRP9 | SNRPC | SNX32 | ST7 | TOM1L1 | TOR3A | TXLNG | ZNF300 | ZNF346 | ANGEL2 | CCDC62 | HSPB11 | ISM1 | LANCL2 | LMBRD2 |
| WDFY1 | MRPS18C | MRPS33 | NUDT3 | FBXO33 | MRPL30 | MRPS21 | NAP1L2 | SPAG17 | TRAPPC14 | ANKRD34B | CARD19 | DNAJC16 | EMC7 | FHIP2A | ISOC2 | MRPL51 | SYT16 | MTPN | RCSD1 |
| BICRAL | ZNF446 | CEP44 | EVI5L | C19orf25 | TMEM183A | AK6 | ANXA2R | C6orf141 | MAGED4 | SMIM11 | S100A7L2 | HSP90AB2P | C11orf98 | MIR1-1 | LINC00632 | LINC00649 | MIR519A1 | TCAF1 | FAM241B |
| HNRNPCP2 | FLYWCH2 | MCRIP1 | PGAP4 | NOXO1 | LSM11 | UPRT | VN1R1 | NIPAL1 | MIR345 | ENSG00000276376 | ZHX2 | NVL | WDR12 | DDX51 | URB2 | SIRPB2 | GBP1P1 | USP17L9P | STAG3L5P |
| ACVR1B | GRIA2 | NR0B1 | SLC41A2 | ACSL6 | CCN3 | CERS6 | HIC1 | MAP4K3 | TP53BP2 | XPNPEP1 | ADGRF5 | GALNT1 | PANX2 | PATZ1 | PHACTR1 | ARHGAP30 | TAF7 | GVINP2 | ENSG00000244255 |
| RTP4 | CDC7 | GGPS1 | ARHGEF6 | CDH13 | DTNA | DHRS9 | ANKLE1 | DCHS2 | CA4 | H2AC16 | C1orf141 | CYP2A6 | PLCH2 | ATPAF2 | CAMKMT | CEP97 | WDFY4 | CMIP | LMBR1L |
| KRT6B | SLFN5 | MIR4435-2HG | CHRM2 | RNA18SN5 | PCK1 | SLC1A7 | CDH7 | SETD1B | CDH19 | CDH20 | CRYBB1 | KCNF1 | CNTLN | MIEF1 | MIEF2 | CES4A | FAM124B | PPP1R3A | EPHA4 |
| CLLU1 | APPBP2 | KRT77 | ANXA2P2 | LINC00309 | MIR502 | TRERNA1 | MIR1245B | DGCR | ERGIC3 | RBM19 | DCAF13 | NIFK | MAK16 | WDR43 | DNTTIP2 | NOL9 | RPF1 | RNF112 | GSC2 |
| TRIM49 | MIR190A | MIR542 | GATA6-AS1 | CDH4 | CLK1 | LAMC3 | PPP2R2C | RHOT1 | UCK2 | ACOX3 | GUCY1A2 | IQSEC1 | LSR | SAR1A | SPAST | STRN | CDYL | ERVK-18 | LCOR |
| LIN28A | IDH3A | MAPKAPK5 | KCNB2 | PRDM5 | SLC27A3 | TSPAN5 | CHAD | KCNG3 | MYCBP | PBX3 | PREB | CDC42BPG | EXOC8 | FOXK2 | LSM1 | MPRIP | RTN4RL1 | CYB5R4 | EMC10 |
| CNRIP1 | MAPK15 | MYOZ2 | PCOLCE | RAB30 | RASGEF1B | TMEM38A | YAF2 | ZNF23 | GPR171 | NREP | ORMDL1 | PCBP3 | SLC16A6 | TCEAL1 | ITPRIP | PALM | PLEKHB1 | TMEFF1 | AP5Z1 |
| HEXIM2 | DACH2 | OLIG1 | PMF1 | SERTAD1 | SLC35A5 | CEP57L1 | DACT3 | DYNLT2 | JSRP1 | MRPL41 | PRPF40B | TAS2R43 | ZACN | AMER2 | DCAF4 | GTF3C6 | H2BC26 | AFG1L | CACUL1 |
| TSPAN17 | HOXC11 | PKD1L2 | RASGEF1C | DNAH6 | FAM222B | TMEM185A | ZBTB41 | ZNF395 | ZNF567 | C4orf33 | CCDC30 | DENND6B | TRIM16L | FBXO46 | LRRC27 | ZNF584 | C2orf16 | KIAA2013 | TRPT1 |
| UMAD1 | TVP23B | URM1 | ZBTB3 | MIR302B | GAPLINC | SLC25A5-AS1 | MIR4500HG | HLA-L | CD99P1 | KRT18P15 | PHKA1P1 | DOT1L | DYRK2 | PPP5C | DVL3 | GFM1 | SFRP4 | WFDC6 | UBALD2 |
| TREH | MIR337 | HOXA-AS2 | PANTR1 | NUCB2 | CD320 | PERP | SH3PXD2A | HEPH | PLAAT3 | SLC39A3 | SOX13 | CLSTN1 | GPR34 | MXD1 | NAA25 | VKORC1L1 | BNC1 | SLC20A1 | SLC16A3 |
| MRGPRX1 | CYB561 | FMOD | MEP1A | CROCC | POLR1G | TECPR1 | ZNF85 | MT1G | PRDM13 | TUSC2 | CHRFAM7A | PCED1A | SF3B4 | CYP2C8 | SSR3 | FGF17 | MACIR | GUCA2A | LANCL1 |
| PCSK7 | MT1F | PLEK2 | SYNPO2 | SRP19 | COPS7A | REXO4 | PWP2 | KLF9 | NOL11 | TNFAIP8L3 | UTP15 | WDR75 | AHSA2P | LINC01736 | PKD3 | KCNJ2 | DAZAP2 | PSORS1C1 | ADRB3 |
| ZFAS1 | ALG2 | NLGN4X | ATXN7 | DRC3 | PHF13 | SLC6A1 | KCNJ3 | TEAD1 | PIP4K2A | PPIF | SLC25A20 | NDUFV2 | PLK3 | AUH | CDS1 | EPHA10 | GJA3 | TTYH2 | BCORL1 |
| TBC1D1 | DPEP2 | DRG2 | MYO15A | UNC5A | ACYP2 | BFAR | EIF5A2 | INCENP | LRRTM4 | MCOLN3 | PAFAH1B3 | PAOX | SPTLC3 | STRADB | YBX2 | FN3K | LIN7B | GLYCTK | SLC27A5 |
| SYT14 | ASIC4 | CADM2 | SH3GL3 | BAIAP3 | CA13 | FOXA3 | GAL3ST1 | METTL21A | MSL3 | PCDH17 | SLC7A3 | SPG21 | USP44 | ABI3 | ADAMTS20 | AMIGO1 | ATPAF1 | SLC4A8 | SSH3 |
| EPB41L5 | ABHD6 | ACSBG2 | ARSK | HIKESHI | NHS | NUDT12 | OSBPL7 | PHF7 | PKD1L1 | PPP1R3F | RAB34 | SHROOM2 | SPNS1 | TCEA3 | THAP5 | BCDIN3D | CABLES2 | CEP70 | CHCHD4 |
| EMID1 | EXOG | FUT10 | GZF1 | KLHL24 | LCORL | NDUFB5 | NIM1K | OLFML2B | PIGZ | RBP7 | RND1 | SLC25A44 | SPATA18 | SPATA6 | SYT3 | TBCCD1 | TMEM87A | DISP2 | DUOXA1 |
| ZNF77 | ERMN | IQSEC3 | KCNG1 | PHOSPHO2 | PLEKHA4 | SLC25A51 | SNX25 | TCP11L2 | VSTM4 | WFIKKN1 | ACP4 | ADAL | ARMC3 | CATSPERG | CCDC113 | CDC37L1 | CDK2AP2 | TTC39B | ZNF544 |
| FBXO24 | HS6ST3 | MMRN2 | OLFML2A | LIN52 | LYSMD4 | MAL2 | NUDT11 | PLAC1 | PLEKHM3 | PRM2 | RGS9BP | SLC66A1 | ZFAND4 | ZNF16 | ZNF516 | ARHGEF37 | CCDC82 | CMYA5 | FAM131B |
| GMEB1 | GARNL3 | KBTBD4 | LDAH | TBC1D10B | TMEM104 | TMEM44 | TRUB2 | ZCCHC17 | ZNF585B | ZNF764 | ZSCAN5A | C3orf18 | CCDC13 | CHCHD5 | DENND2A | EMILIN3 | FAM184A | FAM117B | FNDC7 |
| LCA5L | NECAB2 | OSCP1 | RBM12B | SREK1IP1 | TAS2R3 | TMEM169 | TMEM19 | TMEM218 | TMEM263 | TMEM39B | ZNF18 | ZNF221 | ZNF227 | ZNF568 | ZNG1A | C3orf14 | CLHC1 | KANK3 | KCTD21 |
| LIN37 | MRPS36 | RWDD4 | SLC10A3 | ZNF155 | ZNF225 | ZNF417 | ZNF653 | ZNF83 | ARMC7 | C1orf174 | CMTR2 | IQCN | LNP1 | METTL18 | MINDY4 | MRPL57 | PPP1R35 | HYCC2 | KATNBL1 |
| TMEM223 | RIBC2 | SPACA9 | WDR5B | VPS9D1 | ZNF256 | ZNF322 | ZNF470 | ZNF606 | ZNF671 | ZXDB | AKR7L | ARMCX5 | BRME1 | CRYBG3 | SERP2 | TMEM144 | ZNF530 | PRELID3A | RHNO1 |
| BRICD5 | TMEM74B | TMEM81 | TSACC | INKA2 | MBLAC1 | NUTM2A | PRR22 | TMEM150C | ZNF772 | ZNF837 | ARL17A | C1orf167 | CCDC191 | MIR9-1HG | PABIR3 | ZNF660 | SPRR2F | ZNF654 | ZNF766 |
| KCNJ5-AS1 | C11orf96 | CCDC142 | FAM43B | IER5L | TMSB15B | ZCCHC18 | C10orf95 | ZNF487 | ZNF66 | STAG3L4 | ADM5 | C21orf62-AS1 | FAM157A | GOLGA8N | LINC00311 | SMIM30 | DIRC3 | TMEM267 | TRIQK |
| NACA4P | NPIPB3 | OST4 | FNDC10 | HSP90AB3P | TMEM262 | ANKRD20A5P | SOX21-AS1 | DICER1-AS1 | CST9LP1 | LINC00308 | LINC01234 | MALINC1 | NR2F2-AS1 | STARD7-AS1 | TRIM52-AS1 | CASP16P | HSD11B1-AS1 | FAM229A | LINC01547 |
| ZNF407-AS1 | SMIM10L2A | SMIM26 | TSPEAR-AS2 | NUTM2A-AS1 | PRKAR2A-AS1 | PRKCQ-AS1 | SCOC-AS1 | ZSCAN16-AS1 | ABCA17P | GPR50-AS1 | PCOLCE-AS1 | CKMT2-AS1 | DPY19L2P3 | KRT8P12 | LINC00537 | LOC729867 | NFYC-AS1 | MIR1468 | USP2-AS1 |
| TMCC1-DT | DNAJC3-DT | LINC00938 | MATN1-AS1 | ZNRF2P1 | CYP2T1P | FAUP1 | MIR4512 | LOC100289473 | PHC1P1 | TNRC18P1 | AK4P1 | KRT18P31 | LOC100996437 | SERBP1P5 | EIF3FP1 | ENPP7P4 | HNRNPH1P1 | PCDH10-DT | PSMD6-AS2 |
| LOC643201 | TRAM2-AS1 | TUBBP5 | CENPBD2P | SPR | SLC29A1 | AMPD2 | HTR2C | PFAS | SGK3 | UGT8 | ARHGEF12 | DLG3 | NOL3 | CCNA1 | PTP4A2 | RGS4 | ADD2 | DGKZP1 | KRT43P |
| BIRC6 | SIK3-IT1 | EGID-100124696 | PEA15 | HSPA13 | MTCH2 | PARG | POLM | RFK | RNF43 | SLC38A2 | WDR37 | AFF1 | EXOC6 | KLHL1 | RASGRF2 | RNF10 | NSRP1 | ASPSCR1 | BHLHE41 |
| ZBTB32 | MEP1B | PZP | FAIM | EMC8 | FNDC3A | HOXD3 | PNLIPRP2 | RBM47 | ZFAND3 | MINDY1 | RAB43 | SHCBP1 | ZNF131 | CWC22 | GPSM3 | RAB20 | C6orf58 | RAPGEF6 | TENT5A |
| C1orf43 | ZC3H11A | ATG16L2 | BBX | ZNF732 | ZSWIM1 | CT55 | RNASE12 | TACSTD2 | MOCS1 | GLO1 | EML4 | KRT15 | MIR191 | MIR27B | APOM | AKAP5 | MYOM1 | EMC4 | PRSS33 |
| VPS13D | NEMP1 | ZC3H12B | RNASE13 | MYH8 | MYH15 | MYL7 | MYL5 | MYH16 | MIR223HG | LRAT | STRADA | STYX | NGF-AS1 | BCAR1 | RNASE1 | SLC39A1 | HADH | PIGC | CCDC8 |
| TXN2 | SVOPL | LOC113748420 | ANGPTL1 | NUDT2 | BBOX1 | LMAN2L | PKIA | AASDHPPT | SLMAP | APOL6 | BSN | CAVIN2 | CBX7 | DUSP12 | RAB3B | VTA1 | ZMYM4 | NUMB | CRYM |
| DEPDC7 | FMO1 | EPB41L3 | PI4K2A | TMUB2 | TROAP | ZNF385A | BROX | CRISPLD1 | PYM1 | PRH1 | CCDC168 | GPR33 | HSPA7 | MIR372 | TOB1-AS1 | MIR122HG | BNIP3P6 | LSG1 | JRK |
| ATP1A4 | HERPUD2 | HILPDA | RNFT1 | MYOD1 | CPT1B | TPH2 | SLC20A2 | ADCYAP1R1 | EFEMP1 | ABCG5 | EZH1 | NPY2R | CHSY1 | DFFB | MINK1 | SLC33A1 | ACP2 | AKR1C2 | DSC3 |
| ITGA11 | NKX3-1 | ANO7 | POU5F1B | KIF1C | MCAT | NDUFB8 | RIOK2 | BTG1 | C1QTNF1 | GREM2 | GUCY1B1 | LRFN2 | MFAP2 | PHLPP1 | QPRT | RND3 | ST8SIA1 | ARHGAP1 | CERS1 |
| ARHGAP15 | LRIG1 | CRABP1 | GLIS2 | HSD17B13 | IMPG1 | PHC3 | PITPNM1 | PITX3 | PPP1R15A | PRDM14 | SERPINI2 | SRPX | TIGAR | USP45 | ABCC10 | DTX2 | PALS1 | ADGRD1 | AKAP11 |
| ITM2C | BIK | CUL9 | GSTK1 | PPFIBP2 | RAB17 | RHOBTB3 | SLC37A2 | SLC9A8 | WBP11 | ZDHHC2 | ZFX | APOC4 | ARHGAP18 | ARHGAP21 | CHORDC1 | CLSTN3 | MOGAT2 | C1QTNF3 | CNPY2 |
| QRICH1 | MED28 | MRS2 | NEIL3 | CABP1 | CBX6 | CD99L2 | CIDEB | CPNE5 | DNAH2 | GALNT18 | KCTD9 | NIPAL3 | POPDC3 | SPINK7 | TMEM115 | ZKSCAN4 | FOXQ1 | PDXP | PODXL2 |
| CRYBG1 | RPL38 | TP53INP1 | WDR59 | H2BC5 | MMS22L | MRFAP1 | RHBDD3 | SOX12 | SRXN1 | ABHD17C | FBXL18 | KLHL29 | MT1M | TBC1D32 | TMEM258 | ZKSCAN3 | ZKSCAN7 | TMCC2 | ABTB2 |
| AWAT2 | CSHL1 | DEDD | FOXJ3 | RADIL | ADTRP | RUFY4 | TAAR8 | ADGB | TOMM6 | BNIP5 | BTN2A3P | BORCS8-MEF2B | GJE1 | MIR216A | USP17L24 | USP17L25 | USP17L29 | ZNHIT2 | ARMH4 |
| USP17L27 | FAM81A | RNASE11 | CRIP3 | COA8 | RGL2 | LATS1 | MCFD2 | REG4 | PCYOX1 | ZBTB25 | C16orf95 | MIR1275 | MIR561 | CYP46A1 | LETM2 | CNTN1 | KLK13 | USP17L30 | USP17L26 |
| SHISA6 | USP17L28 | UOX | IGBP1C | KCND2 | FFAR4 | OGA | SH2D3A | SEPTIN8 | DNAJB14 | TTPA | PEX16 | P3H3 | SNORD3A | TTK | PTPRU | FSCN2 | NYX | CATSPER1 | OTOA |
| RNF166 | SPATA46 | MIR518B | ENSG00000235775 | GPR39 | PLEKHA8 | FRMD4B | CALML4 | RGS21 | SNURF | PIRAT1 | MROH3P | CD44-AS1 | LINC00944 | MIR4422HG | LILRB1-AS1 | SPON1-AS1 | LINC02238 | FOXD1 | MIR30C1 |
| ABAT | TMEM106A | ENSG00000288684 | HAL | OPN4 | RPL18A | CELF2 | IGFBP4 | DNAJC19 | MYO1A | PPY | GOLGA5 | HIRIP3 | PDIA5 | ATP2A3 | RNF187 | SLC34A2 | HOMER1 | RPS2P19 | KCND3 |
| AGAP2 | CCNK | PODXL | ERC1 | ING3 | MYT1 | CHMP2A | CHMP3 | ZBTB40 | MAGOH | MRPL38 | CEP192 | RPL22L1 | ZBTB11 | DNASE2B | MAP6 | NAA80 | PIP4P2 | HSD17B6 | HTR5A |
| SLX4IP | CHMP1A | CHST12 | CAPN7 | SNHG14 | MIR485 | MIR3142HG | COX6CP1 | LOC102723403 | TXNDC16 | DMPK | WNK1 | KCNJ5 | COQ5 | MIR92B | MIR608 | MIR658 | ROBO1 | TTC9C | LRRC42 |
| KCNE3 | PRH2 | RBMXL1 | PAGE5 | GATM | GNMT | MAT1A | NDST1 | TNIK | GRIK3 | MARK4 | ADAMTS18 | CACNA2D2 | CHST11 | PDHA2 | XPR1 | CCM2 | INPP1 | PDE2A | AVPR1A |
| PDK2 | SLC6A8 | SLC9A6 | ACOX1 | BOC | CACNB3 | GPRC5A | IDH3G | ULK3 | ASF1B | CHST15 | E2F6 | EDC3 | FARP2 | NAGK | NMNAT2 | PCSK5 | PHF6 | KDM3A | NADSYN1 |
| SLC52A1 | TWIST2 | ARHGAP5 | ARHGEF4 | ARHGAP17 | CBY1 | CDS2 | CLIC4 | CRIM1 | DIDO1 | GNPDA1 | L3MBTL1 | MATN4 | PANK1 | PIP4K2C | RASSF2 | SGMS1 | SHPRH | PRSS21 | RBP1 |
| WBP2 | ST3GAL2 | WWC1 | ALDH1L2 | PDE7B | PLEKHA7 | RAPH1 | RHBDF1 | SRRT | SS18L1 | YY1AP1 | BTBD3 | CDKL2 | CEMIP | CTRB1 | DDX19B | GATA5 | HOXB3 | SLC26A1 | ULK4 |
| LRRC8B | IQCE | NAA50 | NEIL1 | SAMD8 | SCMH1 | SETD5 | SLC25A17 | TAF9B | TNS3 | TOB2 | TSPAN33 | ZDHHC13 | ZNF644 | AMDHD1 | ANKRD2 | CMTM7 | DENND5B | KLF3 | LRRC4C |
| GABPB1 | MMP28 | NAT8 | PANK3 | NIPSNAP3B | OCIAD1 | RFFL | SAP130 | SNX13 | STAM2 | TLX3 | TPCN1 | VPS54 | ZNF292 | ARMC2 | BTBD2 | CLNK | DACT2 | DHX35 | DUSP8 |
| FKBP11 | HNRNPLL | KIAA1549 | NDRG3 | PSTPIP2 | RASAL3 | SIDT1 | TRIM55 | ZFAND2B | ZFAND6 | DCAF12 | ELOVL3 | KLF16 | KRT76 | PCGF3 | PRSS35 | RNPEPL1 | TENM4 | DPH6 | ELAPOR1 |
| ZNF106 | GON4L | PCDHA2 | PCOLCE2 | DISP3 | GPR21 | MFSD10 | MREG | PGBD1 | POLD4 | PRAP1 | PRDM4 | RFX7 | RPAP3 | SAMD3 | SCML4 | ZG16 | BEAN1 | TESPA1 | TEX2 |
| GGTLC2 | ANKRD52 | ANXA8 | C12orf4 | SPZ1 | ZNF467 | C12orf43 | DENND1C | HDDC2 | HMGN5 | IFTAP | LRRC3 | MILR1 | PLPPR5 | SUSD6 | TEX261 | C1orf21 | FAM219A | DMTF1 | FBRSL1 |
| C1orf198 | ITFG2 | ITLN2 | MORC4 | NFKBID | PLET1 | RETREG2 | SERTAD2 | C1orf226 | DBNDD2 | DEXI | NAT8B | NEMP2 | OCSTAMP | SERF1A | SHISAL1 | ZNF658 | CPLANE2 | IFIT1B | ANXA8L1 |
| NSUN5P2 | FAM47E | IFI27L2 | LGALS9C | USP17L5 | NCF1B | FAM47E-STBD1 | AFG3L1P | USP17L13 | USP17L18 | USP17L20 | GGTA1 | MIR181D | USP17L17 | USP17L22 | USP17L10 | USP17L11 | USP17L12 | CYP2D7 | RPEL1 |
| MIR216B | SPINK14 | ZNF726 | TDGF1P3 | LAMA1 | GDF10 | HDGF | MSC | CNGB3 | AKT1S1 | SNAPIN | SRD5A1 | TDRD6 | SLC16A2 | GPR119 | SLCO1C1 | KALRN | KLF7 | USP17L15 | USP17L19 |
| RPS4X | OR1E3 | USP17L21 | USP17L6P | ORC4 | SORL1 | GTF2H1 | HES4 | ENO3 | RAB11FIP2 | CHCHD3 | MICALL1 | MYO7B | LATS2 | BAMBI | CDCA3 | C16orf54 | TTLL5 | SIRPG | SLTM |
| NEUROG3 | KDM4B | CPD | FENDRR | DENND4A | FMNL2 | ZNF436 | BLTP3A | OLAH | MIR133B | MIR484 | NAT1 | LINC00221 | NPHP1 | NPHP4 | GCKR | WDR48 | CYC1 | SLC5A1 | CHKA |
| GTF2H4 | SCARF2 | TAF6 | TGM4 | PRPF40A | EVA1C | MIR9-2 | MIR340 | LINC00887 | KIRREL2 | POC1A | QSOX2 | SLITRK3 | ODR4 | MIR370 | PLPP1 | SLC45A3 | DPP6 | PCDH15 | GBA2 |
| EIF1AX | GRIN2D | PRC1 | YKT6 | FOXN4 | PHF24 | ZNF280B | LRRC74B | CASC15 | MIR584 | SNORA40 | ST3GAL6-AS1 | CCAT1 | LAMA5-AS1 | MIR599 | PDIA3P1 | LINC02258 | MIR664A | GJA4 | GMPPB |
| GLDC | FOXE3 | FOXL1 | OPALIN | APOC1 | PNMT | DGKQ | HMGCS1 | LASP1 | RCL1 | API5 | DHX37 | DTL | ITGBL1 | IPMK | EVA1B | WFDC21P | RPL23A | SKAP1-AS1 | ENSG00000266919 |
| DVL1 | TBCA | GPHA2 | SPATA7 | MIR1285-1 | CASQ2 | TMEM38B | B4GALT7 | TRDN | GLDN | WDR45B | ZNF44 | ZCCHC7 | WDR76 | THAP7 | ZNF396 | ZNF501 | MIR601 | SNAI1 | RER1 |
| MIR637 | GAMT | PNPO | TMEM130 | TNFAIP2 | SPINT2 | SURF1 | SCGB1D2 | MIR503 | MIR361 | NDUFS7 | NDUFA4 | CEP55 | ACAP1 | SNX5 | CST4 | MEX3A | MEA1 | MIR638 | MIR382 |
| SERP1 | MIR622 | NPPC | RPS13 | GDF6 | KCNB1 | CA8 | LGR5 | BICD2 | ANO10 | SLC7A4 | CDK2AP1 | REEP4 | MTRFR | NLRP13 | EXTL2 | ZNF746 | TMEM39A | RABL6 | TASOR |
| MAMSTR | HSF5 | KRBOX4 | MIR625 | LINC01147 | LOC285626 | MASCRNA | MIR7113 | GABRA5 | SLC12A5 | CHRNB2 | CACNA1I | GRIK5 | SCN3B | ALS2 | FZD9 | NLGN3 | NNMT | ALPK2 | ZNF10 |
| CLDN18 | ZNF438 | NAT16 | SNORD19B | FBP2 | FDXR | GALR1 | GDA | KCNT1 | NPFFR2 | RXFP2 | SLC13A3 | SLC13A5 | ZDHHC9 | ACSL5 | AGTPBP1 | CNDP2 | STK17B | ACOX2 | ARSG |
| CBX2 | CPT1C | DIAPH3 | EPHA6 | HOXB1 | KCNH8 | KERA | MFSD2A | PHKA1 | RPE | ABCD2 | BAZ2B | CLN8 | COL27A1 | CYP2F1 | ETNK1 | FOXRED1 | KCNIP1 | TLE4 | AADAC |
| LPAR4 | CHRD | DOCK3 | GALR2 | PHC1 | PIGV | SLC17A8 | SPG11 | TRAK2 | ACBD5 | ACOT11 | CADPS2 | CHST2 | COLGALT2 | DOCK9 | GALNT13 | INMT | KCNK13 | KCNJ9 | LARGE1 |
| LIMD1 | MPG | MYO3B | NEK3 | PCDH7 | PHC2 | PKN3 | POLE3 | RIF1 | SEMA5B | SIN3B | SLITRK5 | SP4 | SRPK3 | STARD13 | SUGCT | TM7SF2 | UHRF2 | KDELR1 | KLF15 |
| CELF4 | MAST4 | ONECUT1 | PARVB | LMOD3 | SCYL3 | SNRK | TSPAN9 | AVIL | BCAS1 | BMF | CMBL | CPXM1 | CSMD3 | EPS8L3 | ERN2 | FAM3D | G2E3 | ACAD10 | B4GALNT3 |
| GPM6B | CHCHD10 | DOCK11 | KCNAB3 | KLHL12 | NCAPH2 | NDOR1 | NIBAN1 | NSL1 | OXGR1 | PBLD | PRSS27 | RAB39B | RANBP17 | RASA3 | RASA4 | RXFP4 | SEMA6C | GDPD5 | GJC3 |
| SPO11 | HOXB4 | INPP4B | KDM8 | YEATS4 | A1CF | AMER1 | ARHGAP28 | ARL6IP5 | ATL2 | B3GALT2 | B3GNT6 | B3GNT7 | BDP1 | BIN3 | BOD1 | C1QTNF7 | CCNB1IP1 | SIDT2 | SLCO1A2 |
| DNAJC15 | STARD7 | TNS2 | TRPM5 | GPR155 | HOXB8 | HS3ST3A1 | IGDCC4 | KATNAL2 | LPIN3 | MARCKSL1 | METTL5 | MEX3B | MFSD8 | MPPED2 | MYOZ1 | NDST3 | NMNAT3 | CFAP53 | CTU2 |
| PHLDB2 | EXPH5 | FAM124A | GLT8D2 | RCBTB2 | RHOBTB1 | RND2 | SLC22A9 | SLITRK4 | SOX14 | SPRED3 | SYNGR3 | TDRD7 | THOC5 | TWSG1 | USP42 | ZNF521 | ZRANB1 | NUDT7 | PAN2 |
| APLF | PKNOX2 | RAB26 | RABGAP1 | ATP13A5 | CNN3 | COX7A1 | CSRNP2 | CTDSPL2 | CTTNBP2 | DHX40 | DNTTIP1 | DPCD | ETV2 | FITM2 | FRRS1L | GPCPD1 | GSTCD | A4GNT | ABLIM3 |
| HSPBAP1 | ARAP2 | ARFIP1 | ARHGAP25 | KRT31 | LCMT1 | LIN9 | MBOAT2 | MESP1 | MXRA8 | MZF1 | NIPSNAP3A | PAN3 | PXYLP1 | RDH16 | RFTN1 | RNF169 | SLC35F6 | HEG1 | HSF2BP |
| STOML1 | ING2 | KIF9 | KMT5C | TTC30B | UPK1B | ZNF160 | CGREF1 | CHMP4C | CLEC4F | EAF1 | EGR4 | FAM83G | FBXO25 | GJD4 | HSD17B14 | JMJD4 | KIF27 | SLC9B1 | STK32A |
| PPP2R2D | TM4SF20 | TMEM184B | TMEM8B | ZC3H8 | ZFP30 | ZNF142 | AMZ2 | AQP11 | CCDC106 | CHCHD1 | DHFR2 | ELMOD1 | ENKUR | FAM110B | GCNT4 | GLB1L2 | KBTBD11 | KRT33A | MEIS3 |
| LDLRAD4 | SNAI3 | SNX8 | VEZF1 | NABP2 | NEURL2 | NPAS4 | NXNL1 | PDIK1L | PLGRKT | PRSS50 | PRSS53 | PRSS54 | SCAI | SH3TC1 | SLC22A10 | SPIC | SPNS3 | KRT28 | LDLRAD3 |
| TOMM5 | LHX5 | MED18 | MRFAP1L1 | ZNF432 | ZSCAN10 | C1QTNF4 | C1orf127 | CAMSAP3 | CCDC127 | CCDC57 | CFAP91 | COX19 | CTRB2 | CYB561D2 | ELAPOR2 | FRYL | FSD2 | SPRYD4 | TENT5C |
| KLHL30 | WWC2 | YIPF6 | ZCWPW1 | NEXMIF | OR1E1 | PGBD5 | PLAAT5 | PLEKHF1 | PRIMPOL | PRXL2A | PTTG2 | RAB19 | RIC8B | SH3YL1 | SLFN12 | SLFN13 | ST8SIA6 | GSX1 | KIAA0513 |
| TMEM178A | LRATD2 | MT1B | NAA38 | WDR89 | ZNF236 | ZNF347 | ZNF420 | ZNF639 | ZNF676 | AMTN | ARMCX1 | ATAD2B | BIRC8 | C10orf71 | C1orf159 | CCDC171 | CDC42EP5 | TARS3 | TEX29 |
| DPPA3 | TMEM248 | TMEM51 | TP53INP2 | FBF1 | FBXO36 | FOXO6 | GLMP | GPR146 | GRAMD2A | GSG1L | HMGXB3 | KASH5 | L3MBTL4 | LGALS9B | LIX1L | METTL21C | METTL25 | CSRNP1 | DAW1 |
| PPDPF | DRGX | EIF1AD | EXO5 | SINHCAF | SPINK13 | STYXL2 | TMEM74 | VSIG10 | ZNF234 | ZNF469 | ZNF493 | ZNF665 | AKR1B15 | ASCL4 | BICDL1 | DYTN | FAM166B | METTL4 | PABPC5 |
| LAMTOR4 | RPS19BP1 | SAXO1 | SFMBT2 | SYCN | TCEAL3 | TMEM102 | AQP12A | BEX4 | BTBD16 | BTBD17 | CDCP2 | CYREN | FAM151B | FAM210A | FAM221A | FAM72A | GARIN1B | HDDC3 | INCA1 |
| GTSF1L | NIBAN3 | PLAC9 | RPUSD2 | NNAT | PANX3 | PCDHB6 | PIERCE1 | SPATA24 | SPINK6 | TAS2R39 | TERB1 | TMEM45A | BBLN | BEX1 | BICDL2 | CYB5D1 | LRRC30 | GARIN2 | GGTLC1 |
| RNF208 | KLRG2 | MALRD1 | NHSL2 | XKRX | ZNF254 | ZNF260 | ZNF611 | ZNF615 | ZNF616 | ZNF681 | ZNF852 | AQP12B | C4orf45 | CFAP161 | CNPPD1 | EID2B | FAM185A | MS4A12 | OTUD6A |
| GARIN1A | TAS2R42 | TMEM234 | TMEM35A | RCC1L | SPDYE1 | TMEM60 | UBL4B | VSTM5 | ZNF488 | AMELY | EDDM3A | FNDC9 | LRP5L | MINAR1 | TMEM225 | TMEM86A | TRIM74 | FAM209A | FHIP1A |
| BOD1L2 | KLHDC7B | LYG1 | LYPD8 | INSYN2B | OR9G4 | SPDYE4 | TCEAL6 | TMEM116 | TRABD2B | VHLL | ALG1L1P | GRAPL | KRTAP8-1 | RASA4B | RNF223 | SERF1B | SKIDA1 | ZNF474 | ATP5MGL |
| PRAMEF8 | CFC1B | FAM209B | FBXW10B | ZNF724 | ZNF728 | CXorf65 | MGAT4D | MS4A4E | ALG1L2 | C16orf90 | FAM72B | MST1L | NDUFC2-KCTD14 | NME2P1 | C19orf67 | COMMD3-BMI1 | FABP5P3 | ZRSR2P1 | KRTAP9-1 |
| MT1L | TEDDM1 | TMEM88B | SMIM23 | SPDYE5 | ISY1-RAB43 | PTTG3P | SNHG15 | CYP2G1P | FAM72C | PRAMEF13 | BCORP1 | LY75-CD302 | MEIS3P1 | MYCLP1 | PDZK1P1 | PRSS47P | FOXO3B | GGTLC3 | HSP90B2P |
| ZC3H11B | NSUN5P1 | PMS2P1 | PRAMEF7 | THUMPD3-AS1 | SEC1P | SPDYE18 | SPDYE21 | SPDYE8 | USP17L23 | CYP2F2P | SPDYE9 | URAHP | DHFRP1 | SPDYE14 | PMS2P6 | SPDYE11 | TSSK5P | MEIS3P2 | MIR205HG |
| CYP2AB1P | PDPK2P | SPDYE7P | C13orf46 | HINT1 | ADCY8 | CYP2J2 | GRIN3B | CCDC88A | RNPEP | STEAP1 | CHFR | ASTN1 | CKMT1B | FUT5 | HOXC10 | PDLIM3 | SPACA3 | SLC23A4P | SPDYE13 |
| LEPROT | STRA6LP | ART2BP | CA12 | GPR151 | VPREB3 | SDHAF4 | KAAG1 | UGT2B17 | TMEFF2 | SERPINB13 | MDFI | MYG1 | PSORS1C2 | MIR215 | GNRH2 | RIMS1 | WNT7B | TOR1AIP2 | ISX |
| CHAF1A | RPL36A-HNRNPH2 | ERVE-4 | SP2 | SDHAF2 | GSTA1 | LINC02605 | DDAH2 | MAN1A1 | NASP | GRIN2C | CMPK1 | HSF2 | EIF5 | BRD1 | HOMER3 | TEAD4 | EPS15L1 | NIPA1 | SLC5A3 |
| FHOD1 | GTF2H2 | ELF5 | GET1 | HTATSF1 | SUGP2 | GINS3 | HOXC8 | MND1 | PPHLN1 | WDR74 | PCNP | GPATCH4 | LENG8 | PNISR | TM4SF5 | UBFD1 | LRRC18 | GRIN3A | ASMT |
| RELL2 | NIBAN2 | CLIC3 | CMTM8 | MIR194-2 | MIR147B | MIR194-1 | ATP2B1-AS1 | RPL3P2 | BMP3 | KCNQ3 | KISS1 | MAFA | VPS11 | KRT12 | ZBTB18 | THAP1 | DENND3 | PPP1R3G | RDM1 |
| LRMDA | ZNF311 | SPANXA1 | SNHG12 | LMLN | RPS23 | CPEB4 | AMH | CLK2 | TIAM2 | INTS6 | SETD6 | MMP26 | STARD3NL | BCL7A | NKAIN2 | OTULINL | TSPY1 | SQOR | AP3M2 |
| MKKS | FMR1-AS1 | HSD3BP4 | CCDC50 | DSCAM | TRPM3 | FGGY | INTS11 | MCUR1 | MACROD2 | PGAP6 | HMGN4 | CCDC122 | HPCAL4 | SYCP2 | ARHGEF10L | SYCE1 | ZBTB4 | MIR92A2 | LDLRAP1 |
| BEND7 | RAX2 | UQCC3 | MAOB | MIR548AA1 | KCNJ1 | PDE6B | DHDDS | ALDH1A1 | GNL1 | TRIML2 | BPIFC | SHLD2 | ENSG00000248993 | MAN1B1 | ALDH1A3 | EXT2 | CDK20 | PCDHB1 | SEPTIN12 |
| CERS5 | TEX12 | TMEM151A | SEPTIN14 | OBSCN | ABCA8 | SOBP | DNAJC9 | DEDD2 | ISG20L2 | VGLL4 | CIAO2A | SYNJ2BP | MEAK7 | ZNF165 | EBF4 | ZBTB44 | POM121C | NR2C1 | ARHGAP35 |
| MIR124-2 | EPB41L2 | NEK6 | NPY4R | LINC00973 | MIR448 | MIR7109 | CLCP2 | LOC100290036 | RPL10A | RPS3A | ZP2 | RNASE9 | ITIH3 | RPL19 | U2AF2 | DRD5 | NDUFAF2 | TINCR | MIR128-2 |
| ASAP1 | MIR128-1 | LINC01123 | MIR636 | SLC43A1 | CYBRD1 | IRX5 | ELAVL3 | GREB1 | PKIB | PHF21B | SUPT20H | BCL7C | PRICKLE4 | ZMAT3 | PAGE4 | PATE1 | VNN3P | PKN2 | MNAT1 |
| PPP2R3A | USP1 | ADAMTS10 | DPM3 | BBS2 | ZNF423 | ATF4 | F2RL2 | GOLGA4 | PDE3A | WNT11 | ARL2 | MIPEP | SLC47A1 | ZFPM2 | LIMA1 | NDUFAF3 | LRRC7 | PCA3 | MIR3940 |
| TADA3 | HEXA | RDH12 | KCNJ13 | PMF1-BGLAP | SPAG6 | IGFBP7 | CLCN1 | GK2 | SLC45A2 | BFSP2 | PMM1 | SLC51A | RPH3AL | CENPS | DRAP1 | CPEB2 | PCDHGB7 | OPN5 | FILIP1 |
| ZNF628 | EFHC2 | FAM234A | THAP3 | MIR433 | HULC | CBR3-AS1 | MIR208A | MIR629 | MIR654 | MIR1207 | MIR575 | MIR612 | MIR642A | MIR611 | MIR769 | MIR583 | MIR596 | TCHP | UTF1 |
| MIR325 | MORN1 | CEMP1 | PRAMEF12 | SLEH1 | SLEN1 | SLEN2 | SLEN3 | SLC5A5 | MAPK8IP1 | SLC36A2 | SLC6A12 | CLCN4 | CLN5 | SMUG1 | TFAP2C | ANGPTL7 | GPR4 | MIR600 | MIR657 |
| SAP18 | MIR602 | LOC107648851 | AGMX2 | ZBTB33 | HJURP | SKA1 | SKA2 | HMG20A | MAGEA10 | POLDIP2 | PPIL4 | TENT4B | ZSCAN25 | ELP5 | PRPF38A | VGLL3 | IMP4 | SLC13A4 | ATAD2 |
| OR2AT4 | BOLA3 | GINS2 | LYPLA2 | KLF14 | ZFC3H1 | ZNF444 | RELL1 | RIPOR3 | SAMD1 | LINC00313 | PPAN-P2RY11 | MEG8 | MIR31HG | GATA3-AS1 | MIR4521 | MIR3682 | MIR1269A | MRGBP | NKX2-4 |
| RPL4P4 | RGL4 | UTP11 | FAM83A | ACO1 | RPL13 | RTN3 | FIBCD1 | QRSL1 | KDM2B | TINAG | GPS2 | QKI | CABYR | PNMA1 | GFRA3 | RPS9 | AHSA1 | GREB1L-DT | MIR4458 |
| PXDNL | FAM207BP | MAGEA4 | MAPK6 | HTR6 | HAS3 | SNCG | SVIL | AADAT | KCNK2 | RRP7A | ECH1 | HBP1 | NUDT5 | UHMK1 | XAB2 | ZNF711 | ADAM2 | RPS8 | GATAD2A |
| DDX31 | LINC02551 | ACACB | NFIB | ZNF24 | BCL9L | CASC3 | NOL8 | ESF1 | NLGN4Y | OSGIN1 | PPP4R2 | ZNF574 | FAM114A2 | HELZ | TIPRL | CEP162 | KIAA1586 | CLIC6 | CRNKL1 |
| ZBTB39 | QTRT1 | CXXC4 | TXNDC9 | ZNF428 | C5orf24 | ZNF579 | RGPD3 | MIR219A1 | MIR431 | MIR409 | OIP5-AS1 | EGFR-AS1 | LINC00184 | LINC01088 | AGAP2-AS1 | ELF3-AS1 | MIR1236 | RGPD8 | SPOUT1 |
| MIR1299 | ZNF646 | ISOC1 | SNRNP27 | PCBD1 | KCNA4 | P2RX6 | FPGS | KLB | RAB33B | SEMA4F | DHX34 | KLHL15 | PLA2G4D | PLB1 | PRUNE2 | PROM2 | ZNF490 | MIR300 | TMEM198B |
| DCAF5 | NKILA | CDB2 | DPAGT1 | SHISA7 | NWD1 | SCGB1D1 | FAAP20 | SPANXD | CASC2 | DANCR | SNHG28 | TDRG1 | PCAT18 | MIR328 | MIR501 | MIR362 | CTBP1-AS | TP53AIP1 | CTAGE1 |
| RP1 | MACROD1 | FAAP100 | OR51E2 | MIR499A | ALDH7A1 | PHF8 | HIBCH | NDUFS6 | BACE2 | MCEE | AGAP1 | CEP63 | STARD3 | DSCAML1 | FAM50A | SDCBP2 | FAM193A | SUZ12P1 | GGCX |
| TRMO | CFAP418 | DDT | DRG1 | ZNF771 | ZNF783 | UQCRHL | LBX1-AS1 | MIR802 | HBA-LCR | LOC107988021 | LOC107988022 | SYT1 | NRF1 | YME1L1 | GCLM | RPL8 | NECTIN3 | FAM76B | KLHL17 |
| LYSMD3 | ATAD3C | BORCS5 | FREM3 | H4C14 | FBN2 | TUBGCP3 | DTNB | TM2D3 | TSC22D2 | FBXL2 | AMN1 | SLC6A2 | SLC22A1 | GTF2B | CPSF4 | VPS18 | MIR363 | RPL9 | OTUB2 |
| ADAM18 | ARL16 | RHOB | HLX | GRM3 | PRKAR2A | CPA6 | SH3BP4 | MCRS1 | MIRLET7F1 | ADSS1 | SRMS | REG1B | TRIML1 | TRIM60 | SSPOP | CACNA1C-IT2 | SHLD3 | GRB7 | ICMT |
| ENSG00000254979 | RUNX1T1 | SCAF11 | HGH1 | ENSG00000281179 | ENSG00000288796 | FUT9 | IRX2 | BAGE | KHK | GALR3 | SLK | RAPGEF5 | ADH6 | ELP4 | TACC1 | PPP1R2 | RANBP3 | CCL15-CCL14 | ENSG00000239920 |
| WDR44 | ENSG00000285082 | ENSG00000261884 | ENSG00000278263 | SPATC1L | SYAP1 | TENT2 | ANKRD42 | MXRA5 | PRADC1 | DBF4B | SS18L2 | CRAMP1 | PPP1R37 | ZNF670 | DCAF4L2 | PRM1 | RTL8C | ALDH16A1 | HEMGN |
| ZNF430 | MIIP | RAB29 | JPT1 | TEX41 | MIR760 | ZFHX4-AS1 | MIR570 | MIR1303 | LINC01934 | MIR1287 | MIR365B | DUX4L1 | SLC25A38P1 | FLT1P1 | UQCRC2P1 | BTF3L4P3 | LOC101927840 | REXO5 | TCEAL9 |
| AFD1 | ANKRD36C | ABHD14A-ACY1 | SATB2-AS1 | ACO2 | FSHB | ATXN1 | MGAT1 | ZIC1 | CELF1 | SLC17A3 | CREB3 | NBPF1 | SPACA1 | VNN2 | EGR3 | CARD16 | ENTR1 | LOC111674463 | LOC111674475 |
| NDUFA13 | LOC111674476 | LOC111674478 | SPON1 | TMOD3 | APOL3 | LY6H | STXBP6 | PCGF6 | HSPBP1 | HSP90AA2P | ATG2B | DEFB117 | ELF2 | IK | GOLGA1 | AURKB | HIPK2 | NECTIN4 | TLE1 |
| RPL7A | FOXO4 | FOXL2 | RPS11 | AKIRIN1 | TRMT61A | CAV2 | NEUROD1 | PLAGL1 | SERPINA4 | PGK2 | MBNL1 | IL6-AS1 | CSNK1D | EPHA7 | GNA14 | NRG3 | CSNK1G3 | USP2 | CALB1 |
| PPBPP2 | TNRC6A | DOCK5 | NRGN | MIR1297 | LOC102724971 | TRA2B | RPS21 | ZYG11B | LURAP1 | CARS2 | GATC | ALPK3 | CSRP1 | STAB2 | RHOBTB2 | RPL28 | CLNS1A | PLPP3 | PPIH |
| GULP1 | KCNE4 | GOLGA3 | SCGB2A2 | MTNR1B | APOA5 | MMP17 | FABP3 | ASAH2 | PDSS1 | ASRGL1 | USP11 | OGDHL | B3GLCT | CLPS | RYBP | SLC17A1 | PDC | KIF20B | TAF5 |
| PCGF1 | DNAJB4 | GPSM1 | MROCKI | PRAF2 | UCMA | MIR3678 | MIR513A1 | TSBP1-AS1 | POMGNT1 | WNK4 | GIPR | UGT2B15 | DDX11 | SLC5A8 | SSTR5 | RAD18 | CYP8B1 | CDHR3 | MSH4 |
| AIFM3 | ZNF263 | ANGPTL8 | DNAJC14 | ALKBH3 | ARSD | COX6C | FAT2 | OPA3 | REPS2 | SH3BP5 | TNS4 | AGR3 | ASZ1 | CREB3L4 | PIF1 | RHEBL1 | SDR16C5 | LHX3 | NELL2 |
| CPLX3 | KLK10 | VPS29 | WDR11 | IZUMO4 | MIEN1 | RSPH14 | ANAPC16 | ZNF334 | CAPN14 | SLC25A34 | CNPY1 | GAB4 | LELP1 | ENO4 | POTED | SLX1B | ZNF280A | CCDC68 | CDYL2 |
| OR6C76 | SLC25A30 | ANKRD50 | DOLPP1 | TBC1D3B | CTAGE4 | RIMBP3B | SMKR1 | FBXL19-AS1 | PCAT4 | PRAC2 | EGOT | OBSCN-AS1 | HMGN2P46 | MIR497 | PTENP1 | HCG11 | MIR202 | SLX1A | C22orf15 |
| FALEC | PRAC1 | TBC1D3 | PCOTH | TBC1D3D | LINC00963 | LINC01564 | MIR498 | PCAT2 | PCAT29 | PCAT6 | RNY3 | TRPM2-AS | CTAGE3P | DRAIC | LINC01138 | NBEAP1 | PCAT7 | SNORA1 | MIR379 |
| DDX19A-DT | PCAT19 | SOCS2-AS1 | SPECC1L-ADORA2A | FAM230B | LL22NC03-63E9.3 | PCAT5 | PCSEAT | POTEF-AS1 | DUXAP9 | XLOC_008559 | XLOC_009911 | XLOC_007697 | TCL4 | USH1G | PGAM2 | ACTN3 | WNT7A | ROR1-AS1 | SPRY4-IT1 |
| GPT2 | PCAT14 | WARS2-IT1 | CCAT2 | PCDH19 | SMYD2 | WNT5B | WNT9A | CPB1 | GCSH | TBX3 | CACNG3 | METTL1 | TUBGCP6 | WNT8B | CCP110 | HHAT | MOCOS | GABRA1 | GRIK1 |
| WNT8A | SHMT1 | MECR | FZD8 | LLGL1 | PRDM6 | RCBTB1 | SETMAR | TMC1 | ARHGEF16 | SNTB2 | TTC3 | UFSP2 | ACSS3 | GALNT16 | KRT83 | NAALADL2 | NIF3L1 | NCOA6 | WNT16 |
| TEX14 | ACP6 | AMY2B | KRT85 | UVSSA | WDR82 | CAVIN3 | CEP89 | CES5A | GREB1L | HES5 | KIF24 | MED9 | NT5M | SH3BGR | TMC2 | ZNF592 | ZWILCH | OSGEPL1 | PAXIP1 |
| RNF212 | CEP104 | SPAG16 | TSSK2 | ACTRT2 | DTWD1 | KCP | PCDHGA5 | PCP4 | PRDM15 | SCRN3 | WTIP | YPEL1 | YRDC | AJAP1 | MANEAL | MRPL36 | PHF23 | FILIP1L | NOA1 |
| GASK1A | TBX10 | TM2D1 | TMC4 | ZNF181 | ZNF30 | ZNF302 | ZNF599 | LENG1 | HES3 | NKX1-1 | OR5H6 | PGBD2 | ZNF287 | ZNF672 | NXF5 | PIFO | TMEM187 | TSPEAR | ZNF141 |
| C4orf48 | NYAP1 | TMC3 | ZBTB47 | DSCR8 | DSCR9 | C9orf57 | NUPR2 | DSCR10 | SMCR5 | AIRN | MTRNR2L3 | LINC00243 | SNORD30 | MIR1825 | MIR676 | SMCR2 | DVL1P1 | FAM180B | TMEM52 |
| KRT16P3 | DSCR4 | SCGB2B2 | C19orf81 | RNR4 | SRP68P1 | ENSG00000273948 | ENSG00000274836 | ENSG00000278817 | LOC100421446 | ENSG00000255174 | ENSG00000224410 | SMCR6 | ENSG00000233708 | LOC106020709 | LOC106020710 | LOC106020711 | LOC106020712 | MIR3944 | LOC105374344 |
| LOC107988025 | WHSC1L2P | COTL1P1 | EEF1A1P43 | DEL1P36 | HBHR | LOC108449888 | EGID-123670537 | NFRKB | CASP8AP2 | RXFP1 | NRXN3 | STC2 | RPS5 | JPH1 | SRSF4 | INA | COL20A1 | LOC107133509 | LOC107988024 |
| GTF2A1 | LOC108745275 | LOC108745276 | WHCR | STIMATE-MUSTN1 | BAIAP2-DT | MYOCD | AZIN2 | ITIH1 | DCAF11 | THTPA | FGF13 | LEFTY1 | MAPK4 | SEMA4G | LMTK2 | NRG4 | FGF20 | RPS25 | TMTC3 |
| SPAM1 | MEPCE | CCDC85C | GSKIP | GRM5 | PYGM | GRID2 | ACAD8 | DMRT1 | MCF2L | SERPINA10 | ARHGAP32 | EN2 | HFM1 | POLR2J | TMPRSS11D | MAML2 | SLC16A9 | CLMP | NPTX1 |
| FAM107A | MAN2A2 | UQCRQ | PNLIP | CDH26 | ZBTB48 | CAGE1 | CARMIL1 | NBPF3 | APOOL | H2AC1 | MT-ND4L | SPINK9 | NBPF14 | HAND2-AS1 | MICOS10-NBL1 | HCG14 | BANCR | STXBP5 | ZFP57 |
| STK39 | FNDC5 | GOLIM4 | MRAP2 | CTCFL | RAB9A | THUMPD1 | EMP1 | NUTF2 | SF3B6 | SYNE3 | DHRS7 | ARL6IP4 | PLEKHA2 | NRDE2 | TSNARE1 | LINC00862 | DEFB109B | PANDAR | ADRA2B |
| RB1-DT | PRIM2 | STK16 | TAF2 | DDIT4-AS1 | EPS8 | SLC8A1 | LDHC | VRK2 | SPARCL1 | GPRC5B | ZNF282 | LINC00301 | SNORD105 | MIR3609 | MIR1184-1 | IGLV1-41 | IGLV2-34 | WAKMAR2 | NRAV |
| LOC111674464 | LINC02560 | LINC02562 | LINC02561 | LOC111674479 | LOC113523647 | LOC113633875 | DYNC2H1 | RRM2 | PIK3C2B | LGR6 | SLC4A3 | EFHD1 | MED6 | SFMBT1 | PPP1R3B | ZBTB10 | ABRA | LOC111674477 | LOC113633877 |
| BCL2L15 | LOC111674466 | LOC111674470 | LOC111674473 | MPHOSPH6 | PLBD1 | ZSCAN26 | KDM3B | DARS1 | HNF4G | PCSK6 | RHOQ | CNTRL | RIN3 | SGPP1 | AAR2 | CEMIP2 | GCM1 | PLEKHO1 | IMPACT |
| ZSWIM6 | GFRA2 | GNPAT | MIP | SPRR2A | TMEM50A | ZER1 | EPPK1 | CEP170B | RSRP1 | POTEM | RNF212B | GYS1 | SLC1A3 | KCNK9 | CACNA1E | SLC18A2 | ADRA2C | NGB | PCNX1 |
| SPG7 | INTS1 | USP35 | ANKRD9 | CNDP1 | NPY5R | UGT2B7 | ASPN | SLC39A13 | B3GALNT2 | CLEC3B | DEPDC5 | GFPT2 | SLC22A7 | ST8SIA2 | KRT3 | SLC22A11 | TIMM17A | LINGO1 | SLC1A6 |
| POFUT2 | SPRY4 | TTBK2 | ALG8 | KLHL8 | NDNF | PM20D1 | FCHSD1 | KATNAL1 | POU4F2 | RTF1 | SLC38A8 | SOX21 | ZZZ3 | CDC123 | RBMS2 | TAMM41 | CCDC180 | TSPAN8 | OLFM1 |
| CAMTA2 | IFT57 | RBMS1 | TOR2A | PRSS58 | TAF1L | TRABD2A | ARMC12 | CYP27C1 | ASCL3 | OCM | C4orf3 | CCZ1B | NOTCH2NLA | GLRA4 | CLPSL2 | KRTAP16-1 | SNHG8 | SLC35D3 | BLOC1S4 |
| MIR374A | MINDY3 | MT-ND3 | OSER1 | SACS-AS1 | NBPF19 | MIR3945HG | MME-AS1 | CSMD2-AS1 | NBPF22P | MIR548D1 | NBPF25P | GH-LCR | LOC106146150 | LOC106146151 | LOC106146152 | ADRA1A | AGRP | MIR100HG | MIR520A |
| TAOK2 | FGD5-AS1 | MIR614 | NBPF26 | RGMB | AMBRA1 | HRK | SGTA | NKTR | TSPAN4 | CAB39L | H4C11 | H4C2 | H4C8 | GSDMC | H4C12 | BLTP2 | H4C4 | MELTF | HAS2 |
| H4C6 | BPHL | RPL29 | LSM4 | CEP164 | TULP1 | KIF7 | DRAM2 | UQCC2 | OPN1LW | CARHSP1 | CHN1 | CRADD | APOD | ARHGEF7 | NEO1 | NFYA | PDIA2 | MMD2 | H4C13 |
| DOK5 | H4C15 | IFITM4P | DEFT1P2 | GCC2 | FKBP15 | MED30 | SMG7 | SPOCK3 | ACTR5 | CDCA4 | ACRBP | MON1B | SARAF | SERPINA2 | LUCAT1 | VTRNA2-1 | DEFB109A | DNER | AMOT |
| CKMT2 | GTF2F2 | TGS1 | DDX42 | CYTH3 | DACT1 | VSX2 | DLGAP2 | FOXN3 | KCNU1 | PYCR3 | VASH1 | ACYP1 | CLMN | IGSF10 | TCEA1 | DDX19A | RBM23 | CRYZ | CA3 |
| ANGEL1 | GSTM2 | NIN | AFM | STON2 | HSBP1 | MAPK1IP1L | TTC7B | ERG28 | FAM98A | MICU3 | GPHB5 | NUDT14 | SCX | ZNF596 | NOXRED1 | RFPL4A | TMED8 | TMEM63C | ADCK5 |
| CLBA1 | CASS4 | FAM91A1 | RPS6KL1 | HCG22 | OR11H7 | ZNF705B | LINC00596 | FAM90A20P | OR4K3 | LINC01140 | FAM90A12P | SCAT1 | RDUR | LINC02224 | FAM90A17 | RRAGAP1 | LOC105371348 | TMEM179 | IGIP |
| ROBO2 | PLEKHD1 | CCDC175 | ZNF705G | PEX3 | TBX18 | GUCA1A | GUCA1B | KLHL7 | LCA5 | PEX11B | BBS9 | MUCL3 | COA3 | CDK5R1 | DHX38 | PKP2 | APH1A | GUCY2D | GRIP1 |
| CHN2 | AIPL1 | NR2E3 | P3H1 | GALNT10 | SMOX | TSNAX | VPS26A | USP3 | CIRBP | NCOA7 | PSD3 | VSIG2 | ANKS1A | ANXA13 | CNTNAP4 | NOSIP | U2SURP | GSTA3 | MICAL1 |
| SELENOS | PITRM1 | TAOK3 | ELOA | C1QL1 | OR2H1 | TM9SF1 | ZC3H15 | EXD2 | RBM15B | ZNF827 | NCCRP1 | PAGE1 | FRMD1 | C1orf68 | SELENOH | LRRC9 | TARP | GSTA5 | RMDN3 |
| TRAJ1 | STK35 | PBX4 | RLN1 | TRAJ25 | TRAJ29 | LY6S | EPHB3 | CSNK1G2 | EPHA8 | GPC6 | SLCO2B1 | FGF12 | INSRR | FGF18 | FGF16 | FGF6 | ANGPTL6 | TRAJ2 | RANP1 |
| GAS2 | TRAJ12 | TRAJ22 | TRAJ24 | PAM | SRI | CYP2C18 | NAA20 | PLCZ1 | ESCO1 | CDCA5 | TPD52L1 | PCMTD1 | S100A13 | SOX7 | TCFL5 | GSTT2 | TNP1 | CER1 | FGF11 |
| RBMXL2 | LMTK3 | CIT | FBLN2 | ADRA1D | MADD | CES2 | NDUFA6 | TARS2 | ARHGAP4 | CNNM2 | DLGAP1 | MACF1 | NEK4 | DGKI | GORASP1 | NACC1 | SENP2 | NRBP2 | ZC3H18 |
| CRTC3 | DEFB109C | ACSL1 | PTH2R | PLCL1 | SFXN3 | CHPF | DDHD1 | OSBPL9 | ARHGAP44 | CENPT | COPS7B | FOXP4 | GALNT7 | SNAPC4 | TMTC1 | ANO9 | CENPK | SPOCK1 | TSPAN31 |
| FAHD1 | DIO3 | DLGAP5 | GLT8D1 | MS4A6A | MTCH1 | SAMD12 | TRO | CENPM | MRPS18A | ZNF536 | ABT1 | ANKRD44 | HASPIN | PCYOX1L | FNDC3B | KHNYN | KIF18B | CRIP2 | EXOC3 |
| TIMM21 | GDF7 | GPR162 | MRPL46 | TMEM71 | SPATA2L | TSNAXIP1 | GML | FBXL21P | SOX2-OT | AHI1-DT | LINC00624 | MIR576 | LINC00381 | SIGLEC17P | LOC109623489 | SLC12A6 | CNGB1 | MS4A14 | RPL26L1 |
| ADGRV1 | RAVER2 | XKR4 | POTEE | RAB28 | TTC19 | AAGAB | FREM2 | SIX5 | STN1 | TTC21B | WDPCP | BBS7 | BCO1 | KIZ | DYNC2I1 | IFT80 | ZNF513 | CRTAP | PEX6 |
| MIR302A | BBS10 | COL8A2 | PEX13 | TPK1 | GPR37 | TNNI2 | ADCY4 | ALAS1 | ARHGEF10 | FARS2 | KCNMB1 | MYT1L | SLC25A10 | SLC4A5 | BRD7 | GPR37L1 | LIMS2 | TESC | DPH3 |
| AS3MT | TMEM176A | AQP2 | MKNK1 | HEPACAM | HS6ST2 | ISYNA1 | KDM2A | MRPL44 | PUS7 | ATG13 | CCNL1 | CHDH | CLASP2 | E2F7 | FAM3B | GEMIN2 | PPP1R10 | MSRA | TESK1 |
| BCL2L13 | CEPT1 | GDF3 | GSTM5 | MRPS30 | NFE2L3 | POMK | SEC11A | SF3A2 | SNF8 | STARD5 | STRN3 | TGOLN2 | ACAP2 | CDX1 | CHERP | COL22A1 | HYAL3 | SRGAP3 | ALDH3B2 |
| MRPL37 | EYA3 | NECAP1 | SLITRK2 | SSU72 | TENM2 | UPK1A | VEPH1 | WDR33 | ATAT1 | BCAP29 | COL28A1 | COL6A5 | EFR3A | LRIT1 | MAU2 | MRPL2 | MSL2 | L3MBTL2 | LMAN1L |
| SH2D4A | MTHFD2L | RASSF3 | SPIN1 | NPFF | SERPINA9 | THEM5 | ARHGEF40 | C9orf78 | CCDC170 | CHADL | DNAH3 | ELAC1 | EPHX4 | IGSF9B | KLHDC3 | PUS7L | SPIDR | RLN2 | RPAIN |
| ZNF410 | CREBZF | GFOD2 | KCTD5 | DNAJB8 | EXOC3L4 | HAGHL | LRIT2 | MROH1 | RFTN2 | SBSPON | SOCS7 | TEX15 | CCDC138 | DNAAF10 | NECAB3 | SLC7A6OS | CMTM1 | TMEM167A | VPS13C |
| HEATR9 | ZNF839 | ZPLD1 | CCM2L | SPIN3 | SPINDOC | MS4A5 | DRICH1 | TIMM29 | TTC6 | C2orf83 | SNORC | C11orf40 | KIRREL3-AS3 | LINC00486 | MIR137 | SMIM27 | TMX2-CTNND1 | DNAJC4 | ENTHD1 |
| CYMP-AS1 | HPF1 | LRRC55 | RNF122 | LOC729968 | ATP5MC2P2 | ATP5MGP5 | LOC441996 | LOC100129725 | GABRA2 | ATP2B3 | SORD | KCNC1 | KCNC3 | MTMR2 | DTYMK | HDAC10 | NTHL1 | PCBP3-AS1 | CYMP |
| COQ7 | LYNX1-SLURP2 | ENO1P1 | UBBP4 | JPH3 | LIPG | AFF2 | ASIC3 | COQ9 | FRZB | PREPL | ADAMTS12 | ANO3 | DHRS3 | HKDC1 | KCNK18 | LDHD | MYOT | AVPR1B | COL9A2 |
| SLC24A5 | GPR143 | NDUFA1 | NPC1L1 | RBPMS | RRAD | SLC5A4 | SLC6A18 | ST6GALNAC1 | SULT4A1 | UNC13A | AATK | COQ4 | FEZF1 | KRT6C | MFF | SHOX | SLC22A13 | NKX6-1 | RAB8B |
| ANKK1 | BSND | CA10 | NBL1 | HSPB3 | KLF11 | MAPRE3 | PUS3 | SLC10A7 | SLC25A16 | SLC25A18 | SLC39A12 | TBC1D20 | TSSK1B | UFC1 | ADAMTSL5 | ANKRD27 | ARHGAP19 | UGT2B28 | ACY3 |
| PGGHG | AQP8 | DCAF17 | ENC1 | VPS16 | ACER2 | B3GALT1 | C19orf12 | MON1A | PGAP2 | DDIT4L | EPC2 | PRODH2 | ALX3 | ARMCX2 | BLOC1S2 | DUSP26 | GLYATL1 | KLHL40 | MBNL2 |
| MCF2L2 | PIGW | RXYLT1 | TEX11 | FAM118B | MAGEH1 | SLC16A13 | HOXC5 | MAMDC4 | MVB12A | RAB41 | SLC45A1 | WDR17 | ZNF582 | CIBAR2 | RPL39L | SYNE4 | TCF23 | KXD1 | MAP7D3 |
| EEF2KMT | RASL12 | ANKAR | CCZ1 | NBPF6 | RTP2 | STPG2 | VN1R4 | NBPF15 | OR2T35 | SMIM24 | TMSB15A | NBPF12 | AADACL3 | ASCL5 | FAM170B | NBPF10 | NBPF4 | VN1R2 | DBNDD1 |
| KCNJ18 | H2AB1 | MROH2A | NBPF11 | SPATA31A1 | IRX2-DT | NBPF8 | H2AB2 | H2AB3 | NBPF20 | CT45A10 | HYMAI | LINC01587 | NBPF7P | BDNF-AS | MIR211 | NPSR1-AS1 | PRSS3P2 | OOSP2 | CSKMT |
| SNHG16 | ATXN8OS | MYCNOS | NBPF9 | MIR95 | KANTR | LINC01013 | MIR1306 | MIR299 | NBAT1 | SNORA30 | TSIX | CD81-AS1 | KCNK15-AS1 | MIR889 | PAUPAR | SRD5A3-AS1 | B3GAT1-DT | DBH-AS1 | DNM3OS |
| MYCNUT | TMEM191B | HAGLR | MIR124-1HG | LUNAR1 | MIR4258 | MIR4761 | PERCC1 | NBPF2P | MIR3668 | MIR4675 | MT-TC | NBPF13P | NBPF18P | NBPF17P | NBPF21P | RNU12-2P | DALIR | NBPF5P | PTPRJ-AS1 |
| LOC100500719 | NHEG1 | PACERR | ETS1-AS1 | LOC106146143 | LOC106146144 | NIDDM3 | GRDX | TNDM | MIR101-1 | MAGEC2 | FDCSP | GABRG2 | HCN1 | OXTR | ADCY2 | BCAT2 | RPS6KB2 | HVBS7 | HVBS8 |
| CA14 | CNC2 | DGS2 | ENSG00000188078 | PMPCB | PRPH | RSPO2 | SLC32A1 | MARS2 | SLC18A1 | TAF15 | TFDP1 | TNNT3 | ADAM23 | CNTN4 | MLH3 | WIPI2 | ARTN | ANO1 | MTMR14 |
| PARD6A | KMT5A | NCAN | PMPCA | CEP135 | CEP250 | E2F5 | ERGIC1 | ERO1B | GRPEL1 | MSI1 | NGEF | RIMS2 | SLC25A27 | UGGT2 | BRD8 | CIAPIN1 | DOC2A | CNKSR2 | DHRS2 |
| PSKH1 | PLPP2 | REEP2 | CCDC88C | H3-4 | HOXC9 | PPP4R3A | RPL32 | SFXN2 | SGSM2 | ZNF331 | ASPG | CCN5 | COX7A2 | DNAJA4 | MAF1 | MRPL23 | THAP11 | LONP2 | POLD3 |
| ARHGAP39 | TDRD9 | TRMT5 | FEZ1 | FEM1C | INSM2 | KCNV1 | LSM6 | MRPL21 | NDUFA4L2 | PITPNC1 | PITPNM2 | R3HDM2 | RAB40C | RCN3 | SLC38A7 | SMPX | SRSF10 | TMF1 | TSPYL2 |
| TULP4 | CEP350 | DPF3 | FAAH2 | LRRCC1 | LUZP2 | OGFOD2 | VPS37C | YIF1A | ANKFN1 | ARL6IP6 | CCSER1 | CHMP4A | DPY19L1 | ERMP1 | FOXRED2 | HOMEZ | LINS1 | TSSK6 | TUBG2 |
| RABEP2 | ATAD5 | DPEP3 | HAUS1 | TLCD3B | YPEL3 | ZSCAN2 | C11orf58 | MIS18BP1 | MISP | PABPC1L | PRR12 | RTF2 | STK32B | TBC1D31 | TRANK1 | WBP2NL | ZNF609 | MED8 | MRPL20 |
| CCT8L2 | REM2 | SLCO5A1 | SYT8 | MUSTN1 | NAT14 | PRRG2 | SCAF1 | SDR39U1 | BORCS7 | DRAM1 | HEATR5A | INO80E | MSANTD2 | PHETA2 | ENKD1 | C4orf17 | FSBP | ASPHD1 | ATP5MJ |
| C11orf87 | FAM53C | H2BC17 | HDX | LRRC69 | OCM2 | SHISA8 | ANKRD63 | C16orf86 | UQCC5 | CCDC26 | BCL2L2-PABPN1 | GOLGA6L4 | TDH | C3orf49 | FAM30A | FRMD6-AS1 | TXLNGY | NRN1L | TM6SF2 |
| MIR137HG | C12orf42 | C16orf92 | C1orf54 | BORCS7-ASMT | EP300-AS1 | TMC3-AS1 | LINC01470 | MIR650 | TRAJ34 | MIR548AJ2 | MIR6891 | TRAJ14 | TRAJ9 | MTCO2P12 | ENSG00000288661 | BPIFB5P | IGAD1 | BAGE2 | LINC01619 |
| NAAA | MIR29B2CHG | MIR7-1 | KLKP1 | CAPN9 | INPP5A | ITIH2 | SNRPG | ETV3 | CAPSL | TMEM158 | LINC01100 | RPL24P5 | RPL13P6 | IMPA1 | RDH5 | DBI | DLC1 | ALKBH5 | LOC110283621 |
| CHRNA6 | CCDC134 | CYP4B1 | GLUD2 | KCNV2 | SULT1A2 | TOP1MT | ADAM11 | AKAP6 | EPS8L2 | FLAD1 | KCNK10 | LRP12 | POLA2 | PTPN21 | SALL2 | TSPAN1 | TTC5 | KCNH5 | ENDOG |
| CDKL1 | HEY1 | GAPDHS | HCCS | ISCA2 | OXR1 | PARP10 | PDE6H | PREX2 | SLC25A29 | SORBS3 | SPART | TMEM106B | ABHD4 | ARL5B | CDCA2 | EDEM3 | FKBP2 | ZFYVE26 | ATE1 |
| KRT81 | COX5B | DDHD2 | FEM1B | THSD4 | AP4S1 | CPNE6 | DAZAP1 | ENOX2 | IGSF11 | LSM7 | SIX4 | SLC39A9 | TLE5 | TRIM44 | ADAM32 | DEGS2 | EMCN | HTRA3 | ITM2A |
| JAKMIP1 | MTMR9 | PAPLN | STMN2 | MRPL17 | MRPL39 | MRPL58 | MTFR1 | MTMR7 | ZFHX4 | ABCF3 | ADAM20 | ADAM21 | ARMC1 | ASIC5 | BRMS1L | COX16 | KRT25 | FBXO43 | GMPR2 |
| MTBP | LSM5 | MRPL13 | MRPL16 | PLBD2 | PLEKHF2 | POF1B | PRPF39 | RLF | RP1L1 | RPRD2 | SHTN1 | SNX16 | STMN4 | TXNDC17 | UNC79 | UNK | ZC3H7A | MBIP | MCMBP |
| CNTNAP3 | NDUFAF6 | NDUFB1 | OSR2 | NPM2 | RALYL | RGS22 | TOX4 | ZNF397 | ZNF652 | BTBD7 | C14orf39 | CLXN | EML5 | FSCB | GPR135 | HAUS4 | HHIPL1 | ZFHX2 | CINP |
| ISM2 | FAM135B | FER1L6 | GRIPAP1 | NYNRIN | PRSS55 | PTGR3 | SEMG2 | SLC38A6 | SLC45A4 | SPDL1 | TMEM260 | TSC22D4 | UBAP2 | UTP23 | VCPKMT | WDR20 | ZC3H3 | HTRA4 | IER2 |
| ABHD12B | KCTD2 | KIFC2 | KLHL38 | RMDN1 | SPATC1 | SPSB3 | TC2N | TOGARAM1 | WDR25 | ZNF251 | CCDC198 | CFAP44 | ETFBKMT | FBXO16 | GOT1L1 | KRT84 | L3HYPDH | ZNF703 | ZNF707 |
| MTFR1L | ETAA1 | FAM168A | GPATCH2L | RPS4Y1 | SMIM19 | TMEM65 | TRMT9B | ZC2HC1A | ZFAND1 | ZSWIM4 | DTD2 | HHLA1 | LRRC74A | MARF1 | PROX2 | PUSL1 | RBM41 | LRRC23 | LRRC24 |
| C14orf93 | NUDT18 | OR4D2 | PRAG1 | HEATR4 | LYSET | MBOAT4 | NKX6-3 | PPP1R42 | R3HCC1 | RBIS | SLC35A4 | TIGD5 | ZNF517 | ZNF623 | C8orf34 | C8orf74 | FAM181A | ACOT6 | BBOF1 |
| MCMDC2 | C8orf48 | CCDC87 | GPR137C | PCNX4 | SPINT4 | SYNDIG1L | ALKAL1 | FAM86B2 | LRRC10B | OR5AU1 | POTEA | POTEG | SLC35F4 | VXN | FAM86B1 | OR11G2 | TMEM253 | KLHL33 | KRBA2 |
| RD3L | MROH6 | OC90 | OR4K15 | TCF24 | TEX22 | TIMM23B | XKR5 | ZNF705D | C14orf132 | CCDC177 | MROH5 | OR4F21 | PPDPFL | SMIM18 | EIF2S3B | LINC02912 | TMEM249 | WDR97 | LINC02914 |
| LINC01551 | ZFTRAF1 | C14orf178 | OR4K17 | RBM12B-AS1 | TPT1P8 | CCDC196 | CCDC197 | FER1L6-AS2 | PRSS51 | REXO1L1P | RHPN1-AS1 | BAALC-AS2 | LINC00588 | ZNF252P-AS1 | FAM87A | FAM90A2P | LY6L | ADAM5 | FAM167A-AS1 |
| C8orf89 | C8orf88 | DHRS4-AS1 | LINC01599 | LINC02906 | NEDD8-MDP1 | OR4Q2 | PRR23D1 | ZHX1-C8orf76 | CIROP | FAM90A5P | LBHD2 | PRR23D2 | CHMP4BP1 | FAM90A13P | FAM90A9 | FOXN3-AS2 | SEC11B | OR4E1 | TUNAR |
| ID2-AS1 | FAM90A10 | FAM90A7 | LINC00511 | C8orf90 | FAM90A14 | FAM90A22 | FAM90A23 | FAM90A8 | HRES1 | IGHJ2 | ENSG00000256500 | REXO1L2P | TRAJ23 | TRAJ37 | TRAJ39 | TRAJ6 | TRAV8-5 | SYNJ2BP-COX16 | FAM90A15P |
| ENSG00000248235 | SDR16C6P | NAMPTP1 | TRAJ17 | IGHJ3 | TRAJ10 | TRAJ27 | TRAJ32 | TRAJ38 | TRAJ4 | TRAJ44 | TRAJ49 | ENSG00000257520 | ENSG00000258989 | ENSG00000259132 | ENSG00000276418 | FAM90A16 | FAM90A19 | TRIM53AP | UGT1A |
| IGHJ6 | FAM90A18 | FAM90A24P | HRURF | TRAJ16 | TRAJ19 | TRAJ20 | TRAJ21 | TRAJ26 | TRAJ28 | TRAJ30 | TRAJ35 | TRAJ36 | TRAJ40 | TRAJ41 | TRAJ43 | TRAJ45 | TRAJ46 | IGHJ4 | IGHJ5 |
| TRAJ5 | TMEM276 | TRAJ11 | TRAJ13 | TRAJ54 | TRAJ56 | TRAJ57 | TRAJ59 | TRAJ61 | TRAJ7 | TRBV21-1 | TRDJ3 | ENSG00000203546 | ENSG00000254692 | ENSG00000257341 | ENSG00000258653 | ENSG00000259060 | ENSG00000259066 | TRAJ47 | TRAJ48 |
| TRAV8-7 | TRAJ50 | TRAJ52 | TRAJ53 | ENSG00000259171 | ENSG00000259522 | ENSG00000259529 | ENSG00000264668 | MAGOH3P | NF1P4 | NF1P6 | TRIM51JP | TRIM64DP | TRIM64EP | TRIM64FP | ENSG00000258417 | ENSG00000258466 | ENSG00000258677 | ENSG00000259164 | IGHD3-3 |
| TRDJ4 | TRDJ2 | TRIM53CP | ENSG00000258691 | ENSG00000285655 | ENSG00000285982 | ENSG00000286131 | IGHD3-10 | IGHD3-9 | IGHD5-12 | IGHD6-13 | IGHD6-19 | ENSG00000271698 | ENSG00000285880 | LOC100132229 | LOC642414 | LOC642579 | LOC106029312 | ENSG00000259371 | ENSG00000273259 |
| LOC112637023 | TRIM51CP | ENSG00000254673 | ENSG00000284956 | KANK2 | GNAS-AS1 | GRM6 | PDE6A | CNGA1 | GNAT1 | ITGA8 | CACNG2 | PDE6D | XPNPEP2 | YARS2 | BEST1 | CACNA2D4 | DLX5 | LOC646506 | LOC102725023 |
| MAP3K6 | PLXNA3 | CRISP1 | TMEM176B | AGGF1 | CYP4V2 | PPM1K | RP2 | BPNT2 | CDHR1 | COX20 | PORCN | RGR | SLC7A14 | TACO1 | CERKL | PEX12 | PITPNM3 | EHHADH | GRK1 |
| LRIT3 | MYO7A | PDE6G | SLC24A1 | IFT27 | LYRM7 | POC1B | RD3 | VSX1 | HMX1 | KCNE5 | GPR179 | OPN1MW | BBIP1 | PET100 | ABCB5 | NCS1 | UBQLN4 | SLITRK6 | AMMECR1 |
| TSBP1 | TRAPPC2 | CABP4 | COX14 | FBXO5 | TM9SF4 | FUNDC1 | OTOL1 | ATG4B | NR2E1 | AGPAT1 | HCAR2 | IQGAP3 | CKS2 | SEMA4C | KCTD3 | LY6G6C | LDOC1 | TAAR1 | PTPDC1 |
| LY6G6E | TH2-LCR | BLVRB | STAP1 | SLC7A1 | HDLBP | ISCA1 | RAB11FIP5 | CSTF3 | HMCN1 | PLXNB2 | CREB5 | CORO7 | GANC | MIA | CALCOCO1 | CST1 | LNX2 | LYZL6 | ADGRE4P |
| ZG16B | MIR101-2 | LINC01198 | ENTPD2 | C5orf64 | HCG9 | HEIH | ADGRB3-DT | SYBU-AS1 | LINC01727 | PRR11-AS1 | GRM1 | NR2F1 | SDC3 | MAN1A2 | MAN1C1 | NDUFA2 | SLC44A4 | POLR1H | TFPT |
| USP32 | PAAF1 | ZC3H13 | LY6G5B | RGN | FBXO3 | HOXA6 | RBM5 | TMSB10 | UBL3 | IRAG1 | L1TD1 | RBM27 | QPCTL | GBA3 | MAGEA6 | C10orf67 | KIAA0040 | ACBD3 | GNPNAT1 |
| MIR378D2 | BTG2 | EMP3 | OPN3 | DIAPH2 | FXYD2 | HSD17B7 | SLC16A7 | SULT1E1 | SV2A | ALPG | INPP4A | SDS | TRMT1 | MAT2B | MTFMT | SCG3 | SULT1B1 | MIR22HG | CARMN |
| RPH3A | ADRA2A | ATP2B2 | AOX1 | NINL | TRHDE | CCNY | CDH22 | FKBP1B | PTOV1 | REEP6 | SPRR3 | TIMM9 | ATF7IP | ATOH1 | BHLHA15 | CEP112 | FBN3 | TSC22D1 | CRYGS |
| JOSD2 | SNIP1 | VPS39 | LBX1 | CDC42EP1 | DNAJC17 | GCC1 | MFAP1 | RTKN2 | SH3BP1 | TIMM10B | USP46 | STRIP1 | TYSND1 | FAM162A | KLHDC1 | MIPOL1 | RALGPS2 | FKBP7 | INTS4 |
| WSB2 | TBC1D10A | TIMM10 | AKAP4 | ICE2 | POGLUT3 | SPATA9 | ZSWIM8 | FSD1L | LRRC28 | LRRC58 | MTARC1 | PRLH | TMEM40 | C1orf50 | CISD3 | METTL25B | MIR744 | TNRC6C | WASHC4 |
| MIR649 | ARSH | CLIP4 | DCUN1D3 | IFIT1P1 | RPL36P16 | EGID-109951028 | HNP1 | NTN1 | MGLL | HTR2B | MED1 | RAD23A | FUT6 | HOXA10 | LIMS1 | MANF | NAPSA | APOA1-AS | MIR579 |
| GTF2F1 | MIR1291 | ENSG00000280987 | ENSG00000198211 | SMPDL3A | SNX3 | TEAD3 | FKBP3 | HLF | KCNIP3 | PDLIM4 | SYNJ2 | CCNG2 | CSTF2 | GCHFR | PPP2R3B | SYMPK | TCF19 | NDUFV3 | THBS3 |
| CEP83 | MGAT4A | MGAT4C | SIPA1L3 | STARD10 | GET4 | KLHDC8A | MAPK8IP2 | ZNF608 | COMMD6 | C1QTNF9B | SANBR | FMR1NB | ADGRF2 | LY6G5C | FAM153A | FAM215A | MIR135B | TMEM98 | ZIC4 |
| MIR26A2 | PHF3 | VPS52 | INTS7 | NAPSB | NRIR | KAZN-AS1 | FAM83A-AS2 | IFNNP1 | MSBP1 | LOC119266102 | ALDH5A1 | GABBR1 | NDUFV1 | PDE10A | PTPRA | ADGRG1 | ADGRG6 | POLR1HASP | HOTAIRM1 |
| MTMR3 | LINC00667 | LINC00342 | LINC01139 | PPP1R1B | ATG4A | BACH1 | BRD3 | KIDINS220 | NUAK2 | SMAD5 | ABR | ANKLE2 | CCNC | GGA3 | MESD | PDE8A | POLD2 | ROBO4 | ESD |
| ADGRA3 | POLR1A | S100A10 | GIT1 | ITSN2 | MTIF2 | PDCD5 | RBFOX2 | RTKN | SENP8 | SLC35B2 | TSHZ1 | WNK2 | OXER1 | STK17A | SUSD2 | TAF9 | CHML | SENP6 | SERINC1 |
| NRDC | BAIAP2L1 | CRISP2 | HSPB2 | RRAGB | SHROOM3 | TNN | TUT4 | ADGRF1 | AP1G2 | DDX55 | EMILIN2 | FHL5 | GTF3C3 | MED24 | MYEF2 | NOVA2 | NRIP3 | DAGLB | GUCA2B |
| SNX17 | PEG3 | PROKR1 | RDH10 | ADGRF4 | HAUS8 | NELFB | SLCO6A1 | CLASRP | ELOF1 | GFOD1 | ZBTB7C | ZNRF3 | CNPY4 | H2BC1 | H2BC13 | NOL7 | OVOL1 | PHOSPHO1 | PIWIL2 |
| ZBTB9 | TMPRSS13 | TXNDC12 | ZGPAT | HCG27 | HCG4 | GAGE12I | GAGE2C | LINC00665 | MAFTRR | SLC1A1 | ADCY3 | SERPIND1 | ECHS1 | METAP2 | PDHB | ARNT2 | DVL2 | SF3B5 | JMJD8 |
| PITX1 | AMY1A | C6orf89 | SFTA2 | TFAP2B | CHRNA9 | FABP7 | KCNH7 | PASK | SULT1A1 | USP24 | ADD3 | MED23 | MKNK2 | NDUFA8 | NME3 | RGMA | SERPINB8 | HMBS | PDE9A |
| LPCAT2 | RAC3 | SLC22A3 | SYN1 | OMG | ST8SIA4 | ARAP3 | ARF3 | DAZL | GALNT6 | GTF2E1 | MAGI1 | MED4 | NFIC | NFYB | SYN2 | TAF5L | TAS1R1 | DSTYK | HIP1R |
| RPL37 | PPM1F | ARFIP2 | ILKAP | TGIF2 | ATG4D | BRAT1 | KIF6 | LAPTM4B | NELL1 | PEAR1 | STAP2 | ZNF202 | BCCIP | BTBD10 | CAPN15 | GPC2 | GXYLT2 | KCNN1 | NAALAD2 |
| OIT3 | RSPO3 | SESN2 | SLC22A16 | SPCS3 | TBPL1 | TRMT112 | TRPC4AP | XPO6 | ZBTB2 | GTF3C5 | HEATR5B | KRT75 | MOAP1 | NPBWR1 | PDCD2 | PHACTR2 | PHTF1 | H2BC9 | MRPS14 |
| SNX18 | PMFBP1 | PRELID1 | SLC43A3 | ZSCAN21 | CTDNEP1 | MED16 | MRPL14 | NSMCE4A | RBM24 | ZNF451 | ELFN1 | KCTD20 | KLHL18 | NUPR1 | PLLP | PRPF38B | RBM4B | SARNP | SCAMP5 |
| CCDC14 | SPPL2C | TMEM214 | ZNF184 | INTS13 | LGSN | MROH7 | SPESP1 | YIPF4 | CLEC3A | H2AC15 | SUPT7L | CCDC34 | CCDC73 | CCDC18 | C5orf46 | C6orf118 | POM121L2 | TMEM123 | ADGRF3 |
| TNXA | CST2 | GSX2 | H2AC13 | GAGE12H | GAGE2E | MIR30C2 | MIR675 | SCARNA5 | AFAP1-AS1 | GAGE12B | GAGE12C | GAGE12D | GAGE12E | GAGE13 | MIR492 | SNHG17 | GAGE2D | SCGB1D4 | GAGE1 |
| GAGE7 | GAGE12J | GAGE10 | GAGE12F | HCG4B | MIR1908 | C5orf64-AS1 | LINC00638 | MIR19B2 | MIR4484 | MIR4505 | DEFB122 | CEACAM22P | MIR3679 | PPIEL | PPP1R3B-DT | SKINT1L | SMAD1-AS1 | GAGE5 | GAGE6 |
| MICD | MIR525 | GAGE2B | GAGE8 | HCG4P11 | HCG4P5 | MICG | RPL7AP7 | YTHDF2P1 | DEFB108D | DEFB108E | HCG4P7 | IFITM3P6 | RPS26P3 | HCG4P3 | HCG4P4 | HEAT2 | MICF | MIR1246 | HLA-W |
| LOC107963951 | MIR3135B | HCG9P5 | HCG4P8 | ACAT2 | CASQ1 | ENTPD3 | PDE1B | PTPRT | SALL1 | AKR1C1 | AMD1 | AQP7 | CRABP2 | DCXR | EFNA4 | LPAR3 | METAP1 | HCG4P9 | LOC113664106 |
| NPTX2 | GRIK2 | CEL | COASY | SCN7A | ATG4C | CNOT2 | CRTC2 | IDI1 | RAB11B | RTN2 | SCD5 | SH3GLB1 | SLC4A7 | SNAP91 | CHM | ECT2 | NLN | UBQLN2 | EARS2 |
| POLR2D | PGC | PIK3C2G | RNASEH1 | TPST1 | UCHL5 | AANAT | ANKS6 | BANP | CABLES1 | CDC42EP3 | CNN1 | COL8A1 | CTPS2 | EMILIN1 | GORASP2 | ITPKA | KIFC1 | NR1D2 | PDE1A |
| PIGF | PTBP2 | RIOK1 | SSH1 | SLC9A9 | STRN4 | SUMF2 | TAF10 | TES | USP48 | CELSR3 | ELOVL2 | ELP2 | INTS8 | NEURL1 | RNPS1 | TOE1 | ACOT13 | MAS1 | MAST2 |
| ANKRD6 | PTGR1 | RSRC1 | SAP30 | IRX1 | PDXDC1 | PFDN5 | PHF12 | PPM1L | RASAL2 | RIN1 | RUSC2 | SAP30BP | SLC35C2 | SYNGR2 | TAF11 | TSPAN3 | UST | ADO | AKAP7 |
| ASPM | APOF | CEP78 | DUS2 | ERGIC2 | GLTP | GMIP | GTF3C2 | L3MBTL3 | LZTS2 | MPP3 | NAT9 | NDFIP2 | NRBF2 | PATJ | PFDN6 | PLEKHA3 | PTBP3 | VCPIP1 | ADPRS |
| UBXN11 | BRMS1 | CRTAC1 | EIF4ENIF1 | ZDHHC19 | ACTL7A | BEGAIN | CUTA | DCP2 | FNBP4 | FNDC1 | GPATCH8 | GPR180 | LETMD1 | LRRC40 | POU3F1 | RAB33A | RBMS3 | RAB11FIP3 | TCF25 |
| RIDA | YEATS2 | ZBTB43 | ZDHHC14 | TMEM181 | TOMM22 | UBLCP1 | USP26 | ZBTB22 | ANGPTL5 | C1QL3 | MYADM | RABAC1 | RIC3 | SLC16A5 | SOX15 | TEX101 | TJAP1 | RDH14 | RIC8A |
| ZPBP2 | SCAMP4 | SHKBP1 | THOC3 | FAM171A2 | MED26 | OR2H2 | SRRM4 | TIMM23 | TRMT6 | VEZT | YOD1 | ZFPL1 | ADGRG4 | CWC15 | DIPK2A | DMXL1 | ERMARD | TMED7 | TMEM147 |
| GLIPR1L1 | CAMSAP1 | CFAP20 | CFAP251 | OARD1 | SCAND3 | THAP4 | UNC5CL | WDR90 | ZNF502 | ZNF804B | ATOSA | CMSS1 | CNOT11 | CPTP | EMC6 | GFRAL | GLYATL2 | FAM107B | FBXL6 |
| PITHD1 | METTL16 | NRM | NUDT16 | SAPCD2 | SFT2D1 | TMEM143 | TMEM242 | ZNF593 | ZSCAN31 | ZSCAN9 | C1orf109 | H2AC8 | NOMO3 | NPS | TFDP3 | TNRC18 | DCAF16 | KLHL23 | NKAPL |
| SERPINB11 | PLD6 | PRKRIP1 | RSBN1L | CLEC18C | POTEF | SPANXB1 | ARL9 | GLYATL3 | SMIM5 | RTL10 | GAGE2A | MIR193B | KIF25-AS1 | MCM3AP-AS1 | MIR206 | MIRLET7A2 | MIRLET7A3 | FAM133B | RFX8 |
| MIF-AS1 | TTC13 | C6orf136 | CCDC167 | FOXD2-AS1 | HCG18 | MIR1307 | MIR320E | IL21R-AS1 | LINC01090 | MIR320B1 | MIR320C1 | MIR378I | MIR411 | MIR4787 | MIR940 | CARD8-AS1 | MIR1203 | ATP6V1G2-DDX39B | LBX2-AS1 |
| MIR548D2 | MIR505 | MSH5-SAPCD1 | FGF10-AS1 | MIR320D1 | MIR4257 | MIR486-2 | MIR151B | MIR320C2 | MIR320D2 | MIR378E | MIR4324 | MIR4476 | MIR4732 | MIR513A2 | MIR588 | STAG3L5P-PVRIG2P-PILRB | HLA-DRB9 | MIR320B2 | MIR378C |
| MIR4638 | PPT2-EGFL8 | HCG26 | MIR1290 | LOC100506023 | MIR2113 | MIR2392 | MIR3616 | MIR378D1 | MIR4481 | MIR4749 | RPL13P2 | DEFA9P | MIR1973 | MIR3714 | IGHV4-55 | NM | LOC110384692 | MIR4467 | MIR4632 |
| LOC117152611 | MIR4741 | MIR921 | ABHD15-AS1 | PRKG2 | GRIK4 | GRM4 | SLIT1 | APBB1 | CHRM5 | DAO | HCRTR1 | HTR4 | MAP3K12 | PI4KB | PTPRG | SLC12A7 | TPP1 | AD10 | LOC117152610 |
| ASPA | ADCY5 | HMGCS2 | PDK1 | CYP4F2 | GABRR1 | KCNJ4 | MTNR1A | MYBPC1 | PICK1 | RPS6KA6 | SLC44A1 | SMOC2 | SNCB | UAP1 | CACNA2D3 | KCNQ5 | MTO1 | UGT1A9 | ACSF3 |
| VPS37A | B3GAT3 | BRSK2 | CDKL5 | ARAP1 | BRS3 | CCN6 | CHD1L | COP1 | COX15 | DGKD | EFHC1 | ELOVL1 | ELOVL6 | GJC1 | GMPR | GPSM2 | GSTO2 | UBIAD1 | VAC14 |
| KCNJ15 | ABCC11 | ABCG4 | ADAMTS8 | NDC80 | NLGN2 | NME4 | OLA1 | OTOF | PFKFB4 | PIP4K2B | PKMYT1 | RGS19 | SARDH | SCUBE3 | SLC38A1 | SLC8A2 | TLE2 | HIPK1 | KCNH6 |
| VARS2 | MAP3K13 | MEIS1 | MTA3 | BNIP2 | CBLN1 | CCT6B | CES3 | CNTN3 | CSTF2T | DENND5A | GABRR2 | GALNT12 | GGT7 | GOPC | IYD | KCND1 | KCNK6 | UGT1A10 | UNC5C |
| NAA15 | AKR7A2 | ARHGEF3 | B3GALT4 | PLXNA4 | PRDM2 | RABGGTB | SEMA3B | SLC6A11 | SLC6A15 | SLCO3A1 | TFB1M | UGT1A7 | WNK3 | ABCB10 | ADGRL3 | ARL6IP1 | BCL2L10 | KDELR2 | MED14 |
| CHRNA10 | NCALD | PCDH9 | PHF1 | DCP1B | DDO | ECE2 | ELL | FAIM2 | HEY2 | HOXA4 | HTR1E | KATNA1 | KCNIP2 | KIAA0319 | LMBR1 | LPAR5 | MAST3 | BRPF1 | CAPNS1 |
| MPZL1 | COPZ1 | CREB3L2 | CWF19L1 | NIPSNAP2 | NRN1 | OMD | P2RY4 | PINX1 | POU2F3 | RFX3 | RHPN2 | RIT2 | RTN4IP1 | SCAMP2 | SLC47A2 | SLC6A7 | SMNDC1 | MCU | MOXD1 |
| SSH2 | MRI1 | NDRG4 | NETO2 | ZFAND5 | ZNF143 | CACNG6 | FMO2 | GTF2H3 | IP6K1 | KCNK5 | LDB2 | MDGA1 | MKLN1 | NMUR2 | OAZ1 | OSBPL5 | RRAGD | SNX1 | SRM |
| SUPT3H | SULF2 | TTBK1 | WAPL | AFF3 | AIG1 | AMY2A | APBB3 | ASXL2 | ATG2A | ATP9A | CBR4 | CBX8 | CHMP1B | CLDND1 | CNOT8 | COL19A1 | COX11 | SIPA1L2 | SNX2 |
| E2F8 | SUPT4H1 | TBL1X | TENM3 | KCNK16 | KDM1B | KLF8 | LRRC15 | LVRN | LYRM4 | MED22 | MED27 | METTL13 | MRPS12 | MRRF | MTX1 | NCBP2 | NCDN | CPSF2 | CUTC |
| RBFOX3 | EGFL6 | GPR158 | GTF3C4 | SLC12A9 | SLC16A10 | SLC17A2 | SLC25A25 | SPOCK2 | SUPT6H | TECRL | TIMM17B | UGT1A3 | USP30 | ZDHHC17 | ZKSCAN5 | ZMYM3 | AKAP10 | NUF2 | PAPOLG |
| BRPF3 | RBM12 | RBM26 | SENP5 | CIR1 | CNOT6 | COX17 | CRPPA | DOK6 | ECHDC1 | ENOX1 | EPB41L4B | FNBP1L | FRMD4A | FXYD1 | GIPC2 | GPR6 | GPRASP2 | AMOTL1 | B3GAT2 |
| ITGB1BP1 | CDK5RAP3 | CENPQ | CEP85 | MOB4 | MRGPRD | NRSN2 | OR11A1 | PAIP1 | PARD3B | PCGF5 | PDZRN4 | PEBP4 | PHACTR3 | PHF10 | POLR2I | RGS8 | RTCA | HECW1 | ISLR |
| S100A3 | KCNJ14 | KLHDC4 | LRFN4 | SLC35B3 | SPRR1B | SSX2IP | SUSD1 | TAF8 | TAX1BP3 | TM4SF1 | TMPRSS11A | TOX2 | TRAM2 | TRERF1 | TRIM54 | TRMT11 | UBXN6 | RTN4RL2 | RUSC1 |
| ZRANB2 | SCEL | SELENOI | SLC26A11 | CACHD1 | CBLN4 | CCNB3 | CPEB3 | DALRD3 | DUSP15 | E4F1 | EHD3 | EIF1B | FGD6 | FRMPD2 | GPR63 | GTF2A2 | HACD2 | ZC3H7B | ZNF41 |
| KCNK17 | ADGRG7 | ANKZF1 | ARHGAP27 | LHX9 | MBOAT1 | MED10 | MRM3 | MTCL1 | MYLK4 | N4BP2 | NDUFA7 | NGRN | NMD3 | NOBOX | NT5DC1 | NXPH1 | PAQR8 | HOXD1 | HSPA12B |
| PIH1D1 | KIAA1217 | KIF26B | KRT24 | RBM6 | RNF145 | RPAP1 | RPRD1A | RPUSD4 | SFSWAP | SMG5 | SPATA5L1 | TBCEL | TMEM11 | TMEM119 | TMEM63B | TPRA1 | TSGA10 | PCDHA7 | PHLDB1 |
| DNPH1 | POLR1E | PRICKLE3 | RAB39A | H1-6 | H2BC14 | KLHL4 | LRRC3B | MAD2L1BP | MAP3K21 | MTHFSD | NRSN1 | PARP15 | PCDHA11 | PDZRN3 | SCAF8 | SLC35D2 | SRBD1 | COQ10A | DERL3 |
| TBCC | FYTTD1 | GSE1 | GSTT2B | USP40 | VPS36 | ZNF512B | ACTR6 | ARMT1 | ATOSB | C1QTNF9 | CDC42SE2 | CKMT1A | CNOT10 | DEF8 | DLK2 | DUS3L | FAM174B | TAAR2 | TBC1D22B |
| FUT11 | TMEM14C | TMEM47 | UNC93A | HUS1B | INTS2 | JKAMP | KLHL31 | LRP11 | MRPL48 | MTFR2 | MYZAP | NCKAP5 | NUDCD3 | OSBPL10 | PCDHA13 | PCDHA4 | PCDHA5 | FAM83B | FRS3 |
| PIMREG | GEMIN7 | HMGN3 | HSDL2 | PTCHD4 | RGP1 | SPATA17 | SPDYA | TAAR6 | TADA1 | TBC1D16 | TMEM131L | UBXN2A | UGT1A5 | UNC13C | VASH2 | VIRMA | VWA7 | PCDHA6 | PDCD2L |
| ZBTB8A | POLR2M | PPIL6 | PRR5L | ZXDC | AIDA | ATXN1L | CCDC174 | CDADC1 | CHP2 | CHTF8 | DENND4B | DMRT3 | ENTREP3 | FAM168B | FAM32A | FAXC | FEV | XXYLT1 | ZBED1 |
| HECA | ZIC5 | ZNF277 | ZNF461 | LMF2 | LRRC57 | LURAP1L | MAS1L | METRN | MLXIP | MOB2 | N4BP2L2 | NAF1 | NOL4L | OR2B6 | OR2W1 | PLEKHH3 | POLR1F | G0S2 | GPRIN3 |
| RNASEK | HEPACAM2 | INTS5 | LIMD2 | SNX4 | SPATA19 | SPC25 | SYF2 | TAAR5 | TCEANC2 | TMEM120B | TMEM25 | TSPYL4 | WDR70 | ZSCAN12 | ZUP1 | ABHD17A | ADAT2 | RAB5IF | RIPOR1 |
| CDC42SE1 | RNF121 | SCRG1 | SNRNP48 | EIF1AY | GPR32 | GTPBP8 | KHDC4 | KIAA1958 | KLHL35 | MOGAT3 | MPND | MTURN | MTX3 | NHSL1 | OBI1 | OR2A4 | OR2B2 | CCDC12 | CCDC83 |
| QSER1 | CST9 | CYLC1 | EBPL | RTP3 | RWDD2A | SELENOM | SMCP | SNN | SOGA3 | TAS2R50 | TCERG1L | TMEM14A | TRABD | TSSC4 | UNCX | ZKSCAN8 | ZNF266 | PRR3 | PRRT1 |
| ZNF831 | RAB40A | RBFA | RBMX2 | OR2B3 | OR2J2 | OR2J3 | RGSL1 | SHISA3 | SPATA33 | SRRM3 | ZSCAN16 | ABRACL | ARL14 | ARPIN | BLCAP | C1QTNF8 | CAAP1 | ZNF419 | ZNF713 |
| CCDC9 | C6orf62 | CCDC28A | H2AC21 | FAM162B | FAM217A | FMC1 | GINM1 | GJB7 | GLT1D1 | IZUMO1R | KIF25 | MROH2B | NXPE3 | OR12D2 | PABPC4L | RGPD5 | SAYSD1 | CCDC125 | CCDC43 |
| SERPINE3 | CWF19L2 | DGAT2L6 | DTHD1 | C12orf75 | C6orf47 | CALHM5 | CASTOR1 | CLEC18B | CXorf38 | DCAF4L1 | DUSP28 | GTPBP6 | KIAA0408 | LCE3D | METTL24 | OR10C1 | OR5V1 | SDE2 | SERF2 |
| SAPCD1 | TSPO2 | TVP23C | ZC2HC1B | ZNF793 | C2orf74 | C6orf163 | CT45A1 | FAM183A | LRRC73 | MCCD1 | MTRES1 | OOEP | OR12D3 | OR14J1 | SMIM8 | TMEM203 | ZNF84 | PTX4 | QRFP |
| MRGPRE | TEN1 | TMEM14B | TMEM217 | LCE3E | LINC03040 | LRRC3C | SMIM13 | CALHM4 | CFAP206 | LINC02693 | PAGE2 | PBOV1 | CFAP107 | PTGES3L-AARSD1 | CTAGE9 | LCE4A | SNHG32 | ZNF98 | ZSCAN23 |
| GAS8-AS1 | RAB40AL | SMIM29 | TRAPPC3L | LINC00574 | LINC02901 | ARPC4-TTLL3 | BVES-AS1 | LINC00242 | CYTOR | CCDC162P | FKBP1C | LINC-PINT | LINC00324 | PITX1-AS1 | RBM14-RBM4 | RPS10-NUDT3 | GAGE12G | MEI4 | TMEM244 |
| OR2B8P | LINC01565 | LINC01600 | AFDN-DT | RNASEK-C17orf49 | BCAR4 | DLEU7-AS1 | MIR124-3 | MIR432 | MIR885 | MMP24OS | HNRNPCL3 | LINC01387 | MIR129-1 | MIR490 | MOXD2P | NKX2-1-AS1 | SNORA55 | GUSBP1 | MT-RNR2 |
| AGPAT4-IT1 | SERPINA13P | SNHG3 | RBAK-RBAKDN | LINC00608 | LINC00707 | LINC00941 | LINC03033 | MIR1296 | MIR452 | MIR517A | MT-RNR1 | PRRT3-AS1 | RNF216P1 | SLC16A1-AS1 | TSPOAP1-AS1 | ADD3-AS1 | FAM111A-DT | ZFP91-CNTF | ZKSCAN8P1 |
| KDM7A-DT | CASC8 | FBXW7-AS1 | IL21-AS1 | MIR4516 | MIR4715 | MIR545 | MIR939 | MIR942 | PCDHA@ | RAB4B-EGLN2 | REL-DT | RNU1-1 | TRBV20OR9-2 | ZNF473CR | FAM85B | HORMAD2-AS1 | LINC01324 | FAM66C | HISLA |
| MUC8 | LINC02580 | MIR1178 | MIR1231 | CFTR-AS1 | ERVK3-1 | GAGE4 | H2AZ2-DT | LINC00278 | LINC02520 | LINC02575 | LINC02579 | LINC02932 | MAILR | MIR1294 | MIR1298 | MTRNR2L9 | PPP1R26-AS1 | LINC01518 | MIR663B |
| HLA-V | PDE7B-AS1 | SERPINB9P1 | VTRNA1-1 | LINC02574 | LINC02576 | LINC02577 | MIR1972-1 | MIR1972-2 | MIR4281 | MIR4311 | MIR548AC | NR4A1AS | RPSAP47 | DEFA7P | DEFA8P | HCP5B | HLA-T | TTTY1 | GOLGA6L5P |
| IGKJ4 | KIF26B-AS1 | LINC00297 | LINC02312 | HLA-U | LINC02578 | LINC02631 | LOC400867 | NOS2P1 | RNU4-5P | RPL23AP1 | DEFA11P | DNAJB1P1 | GAPDHP66 | GTF2IRD1P1 | IGKJ5 | KRT18P13 | KRT18P42 | ICAM4-AS1 | IGKJ3 |
| MIR7705 | LINC01230 | LINC02801 | LOC155060 | SERPINH1P1 | CRPP1 | HLA-P | LINC02967 | MIR7977 | NMBR-AS1 | C5-OT1 | ENSG00000271793 | IGKJ2 | KRT18P16 | LOC102724058 | MIR6089 | MSNP1 | PCDHA14 | LOC157273 | MIR6869 |
| ATP5MGP4 | NOS2P2 | PPBPP1 | PTMAP6 | VENTXP5 | CRIP1P2 | CRIP1P4 | ENSG00000282988 | ERV9-1 | PCDHACT | SERPINA15P | SERPINA7P1 | SERPINE4P | RNA5-8SP | FAM247A | HCGVIII-2 | LOC647132 | KCNJ17 | RPL21P33 | SERPINB8P1 |
| CDR3 | LOC100131395 | PAEPP1 | TRIM26BP | LOC353009 | OTSC2 | ADIPQTL3 | FOXP1-DT | LOC106783509 | LOC107832851 | LOC111188163 | NAXD-AS1 | BMPR1B | GABRB3 | DLG4 | HCN4 | SCN3A | GABRA3 | LOC107303340 | LOC353010 |
| NLGN1 | HPLH1 | LOC353007 | LOC353008 | ACY1 | ASIC1 | CHKB | CHRM4 | MVD | PISD | SCN1B | XYLT2 | CLDN14 | DCX | FZD10 | GLP2R | HTR3B | LARS2 | IHH | MELK |
| WARS2 | ST3GAL3 | ST3GAL5 | ACSS2 | CLCN6 | COX6A1 | FZD3 | KCNC4 | KCNQ4 | ME2 | MPDZ | NAE1 | PAPOLA | TSFM | ADAM22 | CHL1 | CHRNB3 | CLK3 | PDE1C | PTPRM |
| ETHE1 | WIF1 | CDC42BPA | CDT1 | MLC1 | MPC1 | OPHN1 | PRKAG3 | PRKX | PROZ | PRRX1 | SEPHS1 | UTS2R | ARHGEF11 | B3GALNT1 | CACNG1 | CCS | CDH12 | DCPS | DHTKD1 |
| D2HGDH | HIPK3 | KANK1 | LIPF | HAO1 | INPP5J | KIF5C | MAST1 | MPST | NAXE | NDUFAB1 | NTM | PADI3 | PARVA | PFKFB1 | PIGQ | PLCH1 | PPA2 | CDH6 | CTDP1 |
| SIX6 | EFNA2 | GPAA1 | GPC5 | AGXT2 | ARFGEF2 | ATP5MC3 | B4GALT3 | B4GALT6 | BCAM | CCDC6 | CDK17 | CDO1 | CEP41 | CETN2 | CTDSP1 | CXXC1 | DAPK2 | RRAGC | RXRG |
| DYNLRB2 | SLC26A8 | STAG3 | TAF4B | GMFB | GPAT3 | HIF3A | LLGL2 | MGST3 | NKX2-2 | NPRL2 | NT5C | NTSR2 | OSBP | OSBPL2 | PCDH8 | PCYT1B | PPP1R1A | DOCK4 | DUSP9 |
| RAB4A | EDEM2 | GALK2 | GLRA3 | SLC2A8 | SORBS2 | TBC1D7 | TBR1 | TXNL1 | UFM1 | AACS | ABHD2 | ADGRB3 | ANO2 | APBA1 | B4GALT4 | BICD1 | C2CD3 | PRPF4B | PUM2 |
| CRYBB2 | RBP2 | SLC2A12 | SLC2A13 | DLGAP4 | FLRT1 | GABRE | GAS7 | GLYAT | GPR161 | HOXD10 | HOXD9 | HPCAL1 | HTATIP2 | INSIG1 | LIN7A | LRP10 | LYPLA1 | CAPN6 | COX7A2L |
| MPC2 | CTNNBIP1 | CYP39A1 | DACH1 | OLFM2 | OSBP2 | P4HA3 | PADI1 | PCDH10 | PHYKPL | PKDCC | PRCC | PTER | RAD9A | RALGAPA2 | RAP2A | RPS6KC1 | SGSM3 | MAML3 | MED13 |
| SLC25A39 | NDUFB7 | NIPSNAP1 | NME6 | SS18 | SV2B | SYPL1 | VPS25 | ZPBP | ACOT9 | ACSF2 | AIFM2 | ALKBH8 | ALX4 | B4GALT5 | CALML3 | CAPN11 | CETN3 | SHC3 | SIX2 |
| DDX25 | SLC26A7 | SLC9A7 | SMARCD3 | GPR61 | HES6 | HS3ST2 | ITPK1 | LMO3 | LRFN1 | LZTS1 | NENF | NEUROG1 | NPNT | ORC5 | PARP6 | PFDN4 | PPME1 | COL16A1 | DBF4 |
| PRTG | EBF3 | ECI1 | FRMD7 | SEC16B | SFXN5 | SH3GLB2 | SHC2 | SHOX2 | SLC17A9 | SLC26A6 | SLC5A10 | SMYD4 | SORCS2 | TAF1C | TFEC | TFIP11 | TSHZ2 | PPP1R9A | PPP4R1 |
| ACOT2 | PTPRR | RETSAT | RNF144A | APBB2 | ARHGAP12 | ARHGAP22 | ARHGEF28 | ARL13B | ASTN2 | CA11 | CA7 | CALCB | CBLC | CBLL1 | CDH18 | CISD1 | COL21A1 | TSPAN15 | UGT3A2 |
| CTDSP2 | ADAM30 | ADAMTS19 | ADAT1 | DKK4 | DUSP23 | EIPR1 | ENPP5 | EXOC6B | FARP1 | FEZ2 | FN3KRP | FRMPD4 | GPAT4 | HEYL | HMGCLL1 | HOXD11 | KIFC3 | COMTD1 | CPA5 |
| LRRC1 | CTNNAL1 | CYTH4 | DCLK3 | MICU2 | MRM2 | NIT1 | NOSTRIN | PAM16 | PARD6G | PCDHA12 | PCDHA3 | PGLS | PNLDC1 | PPFIA3 | PPP1R7 | PPP6R2 | PSMC3IP | LPCAT3 | LRIG3 |
| RABEPK | LYPD3 | MED12L | METAP1D | SCPEP1 | SLC25A36 | SLC39A11 | SLC9A5 | TFDP2 | TUBE1 | TYW1 | UQCR10 | USP37 | USP47 | WEE2 | ZNF354A | ZNF687 | ABI3BP | RAB21 | RAB25 |
| AMOTL2 | RASD1 | RHOJ | SAV1 | ARPP19 | ART5 | ATP9B | BDH2 | BPNT1 | BUD13 | CDIP1 | CHAC1 | CHST7 | CHST9 | CKAP2 | DCAF6 | DNAH10 | DPPA2 | ADAP2 | ADGRG2 |
| ERI1 | AP3S2 | ARGLU1 | ARHGEF19 | GBGT1 | GGA1 | GJA10 | GLCCI1 | GPR3 | GRTP1 | HAPLN3 | HMG20B | INSM1 | IRX3 | JADE1 | KANSL3 | KAZALD1 | KDM4D | DZIP1 | ELMOD3 |
| KIF16B | FCHSD2 | FNIP2 | GAS2L1 | LXN | LYPD4 | LYPD6 | METTL7B | MIOS | MIXL1 | MRPL19 | NAA60 | NAGPA | NEU4 | NUDT9 | NXT1 | OAZ2 | OGFOD1 | KDM7A | KHDRBS2 |
| PCBD2 | KLF12 | KSR2 | LRRTM3 | PFDN2 | PGP | PIEZO2 | PLEKHA6 | POGK | PPP1R15B | RAB11FIP4 | RAD51AP1 | RPL36AL | SASS6 | SDK2 | SHISA5 | SLC13A1 | SLC2A4RG | PABPC3 | PAIP2 |
| SLC5A12 | PCDH11X | PCDHA9 | PEAK1 | SYNM | SYT5 | SZT2 | TANC2 | TATDN1 | TBC1D2B | TENT4A | TMEM132E | TMEM87B | TMX4 | TOMM34 | TRAPPC8 | TREX2 | TTYH1 | SLC30A3 | SLC37A3 |
| ZDHHC3 | SMAP2 | SNED1 | SRGAP2 | ZNF655 | ZNF79 | ACAP3 | ACBD6 | AFAP1L1 | AGAP3 | AGFG2 | ANKRD12 | ARHGAP42 | ATF7IP2 | ATRAID | B3GNT8 | BTBD9 | C12orf57 | TUBD1 | UQCC1 |
| CNNM1 | ZFP64 | ZNF12 | ZNF268 | DDX43 | ELMO3 | ELMOD2 | ENGASE | EPB41L4A | ERP27 | EVA1A | FAHD2A | FKBPL | FRMD5 | GADD45GIP1 | GALNT15 | GDAP2 | GDPD1 | CATSPER3 | CCNI |
| GPR52 | CSNK1A1L | CTTNBP2NL | CUEDC1 | INTS3 | IPCEF1 | IPPK | KIAA0825 | LAMP5 | LY6D | MAGOHB | MEI1 | MFSD12 | MNT | MOS | MRGPRF | MRPL42 | MRTFB | GEMIN8 | GPR19 |
| NAA40 | GSG1 | HHIPL2 | HOXD4 | OCIAD2 | OVOL2 | PCDH20 | PCDHA10 | PCDHAC2 | PGA5 | PPIP5K1 | PPP1R16A | PTTG1IP | RAB22A | RABGAP1L | RMND5B | RPTN | S100G | MTERF1 | MTRF1L |
| SERINC2 | NKD1 | NOMO1 | NTPCR | SLC23A3 | SMYD5 | SNAPC5 | SNPH | SP8 | SYBU | SYNPO2L | TAS2R16 | TCP11 | TFAP2D | THRSP | TLX2 | TM7SF3 | TMCO3 | SCAND1 | SDHAF1 |
| TMEM53 | SFI1 | SH3BGRL3 | SLC17A4 | TXNRD3 | UBXN2B | UBXN4 | VPS72 | XIRP1 | ZC3H4 | ZMIZ2 | ZNF182 | ZNF195 | ZNF597 | CEACAM16 | CENPO | CPSF7 | DMWD | TMEM161A | TMEM161B |
| FAM120B | TTC17 | TTLL4 | TXNDC11 | GTF2A1L | IZUMO1 | KCNMB2 | MDM1 | MFSD11 | MMGT1 | NDUFAF7 | ORMDL2 | PARP16 | PCDHA1 | PGPEP1 | PHTF2 | PIP5KL1 | PIWIL3 | DNAH17 | ECHDC2 |
| SBK1 | FOXF2 | GAL3ST2 | GIGYF1 | TMC5 | TMCC3 | TMEM132C | TMEM91 | TRIM16 | WIZ | ZCCHC2 | ZMYM1 | ZNF185 | ZNF407 | ZNF496 | ACTL9 | ANKEF1 | ANKRA2 | PLCXD2 | PWWP2A |
| ARL5A | SNX7 | SPIRE2 | SRFBP1 | CCDC91 | CEACAM21 | CEP131 | CLVS2 | CMC2 | CNKSR3 | CREG2 | DDX53 | DELE1 | DHRS13 | DPY19L4 | DUSP18 | ECT2L | FAM114A1 | ARHGAP8 | ARL4D |
| FBXO45 | BARHL1 | BTF3L4 | C1orf87 | GLI4 | GPATCH1 | GPR142 | HAUS5 | HMCES | HORMAD2 | HPDL | KANSL2 | KAZN | KRT36 | LGALSL | LIPT2 | MAGI3 | MALSU1 | FAM53B | FAT3 |
| NANOS2 | FOXS1 | FRMD8 | GAS2L3 | OR6A2 | PAQR4 | PCDHGB1 | PDZD3 | PDZK1IP1 | PGA3 | PNRC2 | PRDM7 | PRRT3 | R3HDM1 | RBM42 | RIMKLA | RIMS4 | RNF152 | MICOS10 | MZT2B |
| RPUSD3 | NSG2 | NUDT16L1 | OIP5 | SLC25A43 | SLC35F1 | SLC35F2 | SNAPC1 | SNX12 | SOGA1 | SPAG4 | SPTSSA | SRCIN1 | SRL | SYDE1 | TADA2B | TCTE1 | TICRR | RNF181 | RNF38 |
| TMEM240 | SAMD11 | SH3RF3 | SLC10A4 | TTC30A | TTYH3 | UBL5 | UBTD2 | UNC119B | USHBP1 | VOPP1 | VPS37B | WBP1 | WDTC1 | YPEL2 | ZDHHC16 | ZDHHC21 | ZDHHC23 | TMCO5A | TMEM184A |
| ZGRF1 | TMEM245 | TP53I11 | TRNAU1AP | ZNF212 | ZNF264 | ZNF445 | ZNF519 | ZNF668 | ZSCAN4 | ZSWIM5 | ANKRD53 | AP1AR | AP5B1 | APOBR | ASNSD1 | ATOH8 | ATPSCKMT | ZDHHC24 | ZFP90 |
| CCDC121 | ZNF124 | ZNF174 | ZNF200 | CYB561A3 | CYP4F8 | CZIB | DESI1 | DGLUCY | FAM43A | FANK1 | FATE1 | FBH1 | FOXD4 | FSIP2 | GAREM2 | GDAP1L1 | GOLGA7B | C2CD2L | CCDC112 |
| GRAMD2B | CCDC97 | CDV3 | CENPW | KLHL32 | KRT79 | LHFPL5 | LINGO3 | LTO1 | MARVELD3 | MED31 | MFSD4B | MIF4GD | MIGA2 | MOB3C | MRPL27 | MSL1 | NPEPL1 | GOLM2 | GPX6 |
| PCDHA8 | GTF2H2C | JMJD7 | JRKL | PM20D2 | POP7 | PRTFDC1 | R3HCC1L | RHOXF1 | ROPN1 | SCRT1 | SERHL2 | SLC16A11 | SLC18B1 | SMAGP | SNX22 | STYXL1 | TAS2R40 | NXPE4 | OVCA2 |
| TGFBRAP1 | PCDHAC1 | PHACTR4 | PID1 | TMEM17 | TMEM170A | TRIM66 | UBXN7 | VGLL1 | WDCP | WDR27 | WDR91 | ZCCHC24 | ZFP2 | ZMAT2 | ZNF33B | ZNF35 | ZNF385D | TBC1D17 | TCTA |
| ZNF571 | THNSL2 | TIGD4 | TMCC1 | ZNF92 | ZSCAN18 | ANKRD13C | ANKRD22 | APOLD1 | ATP1B4 | AXDND1 | BRINP2 | C18orf25 | C20orf27 | C4orf19 | CAPS2 | CCDC51 | CYRIA | ZNF460 | ZNF518A |
| DCUN1D4 | ZNF674 | ZNF778 | ZNF791 | DMRTC2 | DNAH12 | DTWD2 | EID2 | ERI2 | FAM13B | FAM50B | GDPD4 | GPR75 | HECTD4 | HINT3 | HMCN2 | IER5 | JPT2 | DCAF10 | DCDC1 |
| LYPD6B | DENND2D | DEUP1 | DIRAS3 | MXD3 | NACC2 | NCKAP5L | NXPE1 | OGFRL1 | OTOP1 | PCNX3 | PLEKHG7 | PRR14 | PRSS48 | PSMG4 | PURG | RAB6C | RASL10B | KIAA1191 | LEMD1 |
| SFT2D2 | MAP3K7CL | METTL8 | MIDN | SNRNP35 | SPRING1 | SPRYD3 | SRSF12 | TAF1D | TCP11L1 | THSD7B | THUMPD2 | TMEM101 | TMEM200A | TSTD1 | TTLL2 | UBXN8 | USPL1 | RNF183 | RSBN1 |
| VWC2L | SLC35G1 | SLC66A2 | SLF2 | ZBTB6 | ZNF226 | ZNF232 | ZNF273 | ZNF33A | ZNF354B | ZNF383 | ZNF440 | ZNF554 | ZNF581 | ZNF70 | ZNF785 | ZNF90 | BEND6 | UTY | VGLL2 |
| CCDC54 | XKR6 | ZBED2 | ZBTB5 | CEACAM19 | CEP295 | CEP95 | CFAP36 | CLEC2L | COX8C | DAOA | DNAAF9 | EEIG1 | EVX2 | HDGFL1 | KHDC1 | KIAA1549L | MAP6D1 | BMERB1 | CCDC38 |
| SCML1 | CCDC7 | CCDC77 | CDC20B | WDR13 | WDR24 | ZFY | ZNF484 | ZNF556 | ZNF580 | ZNF605 | ALKAL2 | ANKRD33B | ARMH3 | ATXN7L3B | C17orf49 | C19orf44 | C19orf48 | MSGN1 | MYCBPAP |
| CFAP47 | SLC26A10P | TMEM151B | TMEM205 | FHIP1B | GNRHR2 | GPBP1L1 | HSD11B1L | IFFO2 | IQUB | KIAA1614 | LGALS7B | PCED1B | PROSER1 | PRR14L | PXDC1 | RHOXF2 | RUSF1 | CCDC71L | CCNYL1 |
| SIGLECL1 | CITED4 | CNEP1R1 | CSTPP1 | SPATA21 | SPIN4 | SPX | STARD9 | SYS1 | SZRD1 | TMEM236 | TUSC1 | VWA3A | WASHC2C | ZGLP1 | ZNF296 | ZNF696 | ZNF747 | SEC22C | SH3BP5L |
| ZSCAN1 | SLC35E4 | SOWAHA | SPANXC | ASAH2B | C12orf76 | C1orf122 | C1orf162 | C1orf53 | CARD18 | CFAP100 | COPS9 | CRYGN | DDTL | DENND11 | DPPA5 | EDRF1 | EEIG2 | ZNF768 | ZNF787 |
| FAM120AOS | ZSCAN32 | ZSWIM3 | ARMCX4 | HIGD1C | KIAA1522 | LRRC61 | LRRC70 | MCRIP2 | MORN2 | MS4A6E | ONECUT3 | PGA4 | PIRT | PLAAT1 | PROSER2 | PRRT4 | RIMBP3 | ENHO | ERVMER34-1 |
| TMEM114 | FAM131A | FAM177B | H4C7 | TMEM241 | TRIR | ZNF391 | ZNF614 | ZNF714 | AMY1C | ANKRD65 | ARL5C | BAHCC1 | C9orf131 | CFAP46 | CGB1 | CGB2 | EOLA1 | RITA1 | SLX9 |
| KLHL34 | TMEM141 | TMEM164 | TMEM215 | MYRFL | ODF3L2 | OPRPN | PRR18 | PRR35 | PXT1 | RBBP8NL | REX1BD | SAMD5 | SLC49A3 | SMIM20 | SOWAHC | SPATA1 | TCEANC | FAM229B | FDXACB1 |
| TMEM170B | KRTAP2-4 | MFSD14B | MS4A15 | ZNF774 | ZSWIM9 | ARL17B | C10orf55 | C16orf87 | CCDC9B | CCER1 | CEACAM18 | CYSRT1 | FAM205A | KRBOX5 | RTL6 | SH2D7 | STEAP1B | TEX56P | TLDC2 |
| C12orf71 | XAGE2 | ZCWPW2 | ZNF594 | KRTAP2-1 | KRTAP4-11 | MAGEA2 | MYADML2 | NPIPA1 | OR10G7 | PNMA6A | POTEI | STMND1 | TGIF2LY | TMEM178B | TTC24 | WT1-AS | ZFP41 | ZNG1B | ARMH1 |
| C3orf80 | C6orf132 | CCDC169 | CFAP54 | FAM25A | FOXD4L3 | FREY1 | KRTAP4-8 | LEUTX | NOPCHAP1 | POTEJ | PTGES3L | RPL41 | STPG4 | TEX47 | TMEM35B | WASHC2A | ZNF587B | AMY1B | ANKUB1 |
| ZSCAN5B | CCDC159 | CENPBD1P | FAM200B | DCAF8L2 | DELEC1 | EFCAB9 | FAM228B | HSFX1 | KIAA0087 | KRBOX1 | KRTAP2-2 | PATE3 | SHISAL2A | SMLR1 | TMEM250 | C6orf52 | CLDN25 | ZNF737 | ZNF891 |
| PRR36 | C20orf173 | CEP295NL | CLPSL1 | KRTAP4-7 | RNF32-DT | SERHL | SLC22A20P | SMIM6 | SMIM9 | SPANXA2 | ST20 | TCP10L3 | ANKRD30BL | ANKRD66 | C11orf91 | C17orf100 | C2CD4D | KHDC1L | KRTAP2-3 |
| H2AC19 | ZNF316 | ANHX | INAFM1 | TCP10L2 | TRAPPC2B | ADARB2-AS1 | ARPIN-AP3S2 | ATP1A1-AS1 | IQCJ-SCHIP1 | LRRC53 | MUC22 | PART1 | PRAMEF18 | ANKHD1-EIF4EBP3 | ANKRD26P1 | C8orf17 | CORO7-PAM16 | GGNBP1 | GRIFIN |
| LINC00299 | IQCA1L | NBR2 | SLC22A31 | SNHG11 | SPDYE6 | TSTD3 | C10orf126 | CENPS-CORT | HOXA11-AS | INE1 | JPX | SLC7A5P2 | B3GALT5-AS1 | FMC1-LUC7L2 | HOXB-AS3 | LINC00310 | LINC01556 | DNAJC25-GNG10 | LINC00269 |
| ST13P5 | MKRN2OS | OR2J1 | PRAMEF19 | BCYRN1 | EGFEM1P | EPB41L4A-AS1 | HAS2-AS1 | HNF1A-AS1 | IQCM | LINC00312 | LINC00339 | LINC01549 | LINC01588 | LINC01590 | LINC02210 | MIR454 | MIR616 | LINC01558 | SCARNA6 |
| MYMX | TRPC5OS | ZNF137P | ARMH2 | SCARNA7 | SNORA73B | SNORD118 | TRIM39-RPP21 | TSNAX-DISC1 | WEE2-AS1 | ANKRD19P | BPIFA4P | C1GALT1C1L | CCDC192 | CLRN1-AS1 | CLUHP3 | DNMBP-AS1 | HNRNPCL2 | MIR9-3 | MIRLET7BHG |
| HSP90AB4P | NTM-AS1 | PPP1R2P1 | PRORSD1P | LINC02911 | MIR1224 | MIR129-2 | MIR589 | MIR877 | NR2F1-AS1 | RN7SL2 | SNORD17 | SNORD20 | SNORD24 | SSR4P1 | TMEM265 | BRD3OS | GLYCAM1 | HOXA-AS3 | HOXA10-AS |
| LINC00598 | LINC00092 | LINC01548 | LINC02871 | SCARNA2 | SNORA73A | SYS1-DBNDD2 | TGIF2-RAB5IF | TMEM191A | TPTEP2-CSNK1E | CCDC200 | CCDC28A-AS1 | EXOC1L | GACAT2 | GUSBP2 | HSPB2-C11orf52 | LCN1P1 | LINC00029 | HLA-J | LINC00240 |
| LINC00545 | MIR210HG | MIR487B | NDUFV1-DT | MIR133A1 | MIR1915 | MIR323A | MIR495 | MIR519B | MIR520D | MIR543 | MIR671 | MIRLET7F2 | MKRN4P | MRPL23-AS1 | RNU12 | SCARNA11 | SCARNA4 | LINC00239 | LINC00452 |
| SNORA52 | LINC02869 | LOC730098 | MIR1-2 | SNORD15B | SNORD55 | SNORD58B | SNORD69 | SNORD83A | SNORD83B | STX16-NPEPL1 | TTTY14 | UBE2F-SCLY | B4GALT1-AS1 | DISC1FP1 | DLGAP4-AS1 | FAM237A | FAM66A | SNORA13 | SNORA14A |
| HPN-AS1 | SNORA53 | SNORD104 | SNORD12B | LINC00910 | LINC00964 | LINC00977 | LINC01104 | LINC01433 | LINC02874 | LINC02981 | LIPT2-AS1 | LOC93622 | MBNL1-AS1 | MCF2L-AS1 | MCPH1-AS1 | MGAT3-AS1 | MIR1228 | FLNB-AS1 | HNRNPUL2-BSCL2 |
| MIR190B | INTS6-AS1 | LINC00273 | LINC00290 | MIR548A3 | MIR548C | MIR619 | MROH7-TTC4 | NNT-AS1 | OR12D1 | OR1F12P | PRKG1-AS1 | PTPRVP | REELD1 | RN7SL3 | RNF213-AS1 | SCGB1C2 | SNHG19 | MIR1301 | MIR1304 |
| SNORA80E | MIR23AHG | MIR383 | MIR526A1 | SNORD14C | SNORD15A | SNORD16 | SNORD28 | SNORD35B | SNORD42A | SNORD61 | SPEN-AS1 | TIPARP-AS1 | TTC41P | ZNF663P | ADPGK-AS1 | ATP8B1-AS1 | BHLHE40-AS1 | SNORA26 | SNORA3B |
| CCDC183-AS1 | SNORD10 | SNORD101 | SNORD13 | GAS5-AS1 | GAS6-DT | GCC2-AS1 | GOLGA6L7 | HDHD5-AS1 | HM13-AS1 | INO80B-WBP1 | LINC00583 | LINC00587 | LINC00907 | LINC01014 | LINC01094 | LINC01132 | LINC01355 | C2orf74-DT | CCDC144NL-AS1 |
| LNCARSR | CCNYL3 | FAM226B | FLJ40194 | MIR1238 | MIR1248 | MIR1260A | MIR1286 | MIR1292 | MIR2861 | MIR3188 | MIR376B | MIR487A | MIR5010 | MIR526A2 | MIR618 | MTUS2-AS1 | PRC1-AS1 | LINC01446 | LINP1 |
| RNU4-2 | LOC100130691 | LY6E-DT | MIR105-2 | SLCO4A1-AS1 | SNORA23 | SNORA7B | SNORD18B | SNORD18C | SNORD25 | SNORD26 | SNORD3B-1 | SNORD58A | SOX9-AS1 | UNC5B-AS1 | USP46-DT | WWTR1-AS1 | ZNF723 | PXN-AS1 | RNU4-1 |
| BALR6 | RPL13AP20 | SAP30L-AS1 | SLC8A1-AS1 | LINC00327 | LINC00410 | LINC00513 | LINC00865 | LINC00899 | LINC01419 | LINC01625 | LINC01704 | LINC02891 | LOC154449 | MGC16025 | MHRT | MIR105-1 | MIR3614 | ZNF840P | ARAP1-AS2 |
| MIR5196 | CARS1-AS1 | CRTC3-AS1 | GTF3C2-AS1 | MIR663AHG | MT-TV | PKN2-AS1 | RNA5S11 | RNA5S12 | RNA5S15 | RPL37AP8 | SH3TC2-DT | SNORA50C | SNORD1C | SNORD58C | THAP9-AS1 | TPI1P2 | TTC39A-AS1 | MIR4449 | MIR4687 |
| ADAM3A | MIR524 | MIR527 | MIR568 | DUSP5P1 | FAM230I | FOXCUT | LANCL1-AS1 | LINC00244 | LINC00294 | LINC00870 | LINC01276 | LINC01346 | LINC01473 | LINC01714 | LINC01963 | LINC02656 | LOC102723566 | VN1R10P | ZNF300P1 |
| MIA-RAB4B | CASC18 | CERS6-AS1 | CRYBB2P1 | MIR3181 | MIR3187 | MIR3591 | MIR585 | MIR642B | MMADHC-DT | MSH2-OT1 | MSTO2P | OR51C1P | P2RX5-TAX1BP3 | PISRT1 | PPP3CB-AS1 | PSMB8-AS1 | RAMACL | LOC642484 | LRRC37BP1 |
| RNA5S10 | MIR1245A | MIR1910 | MIR3130-2 | RNA5S2 | RNA5S3 | RNA5S4 | RNA5S5 | RNA5S6 | RNA5S7 | RNA5S8 | RNF5P1 | RNU5F-1 | RNU6-8 | RNVU1-18 | SDHAP1 | SENP3-EIF4A1 | SEPT5-GP1BB | RCAN3AS | RNA5S1 |
| SNORD3B-2 | RNA5S13 | RNA5S16 | RNA5S17 | ZNF37BP | ZNF433-AS1 | ADAM6 | BABAM2-AS1 | DNM1P35 | GNG14 | GOLGA2P7 | IATPR | KIAA2012-AS1 | LINC00492 | LINC01149 | LINC01475 | LINC01833 | LINC02218 | SLIT2-IT1 | SNORD14E |
| LOC730100 | SNORD3C | SNORD87 | ZBTB11-AS1 | MIR1268B | MIR3130-1 | MIR3652 | MIR3677 | MIR371B | MIR4443 | MIR4660 | MIR548K | MIR570HG | MIR630 | MKNK1-AS1 | MT-TE | OR2A1-AS1 | PGAM1P5 | LINC02499 | LOC728158 |
| RNU1-2 | LY6G6F-LY6G6D | MAN1B1-DT | MIR1234 | RPL17P39 | SDHAP2 | SLED1 | SMG1P3 | SMIM40 | SNORD76 | SNORD80 | SRP9P1 | SSU72P8 | TMA7B | UBE2Q2P2 | USP6NL-AS1 | ZNF236-DT | ZNF890P | RALY-AS1 | RNA5S9 |
| ENSG00000241489 | RNU1-3 | RNU1-4 | RNVU1-6 | HOXA10-HOXA9 | LINC01144 | LINC02347 | LINC02431 | LINC02667 | LINC02702 | LOC100129066 | LOC101059948 | LOC118142757 | LOC286059 | LOC442028 | MIR3156-2 | MIR4634 | MIR4788 | DHFRP2 | EEF1A1P6 |
| MIR7706 | FAM86B3P | FLJ38576 | FOSL2-AS1 | RNVU1-7 | RPL17P7 | RPL18AP3 | RPL7P23 | SDIM1 | SMG1P5 | SMIM28 | TBC1D3P1-DHX40P1 | TBC1D7-LOC100130357 | TDGF1P2 | WASIR1 | XBP1P1 | ALG9-IT1 | ARHGEF34P | MIR6125 | MIR6515 |
| EEF1DP1 | OR5BS1P | PTGES3P1 | RAP1BL | HNRNPA1P33 | LOC100289495 | LOC729254 | MGAT4FP | MIR5571 | MIR6797 | MIR6813 | MIR6821 | MTHFD1P1 | PIN4P1 | PRG1 | RNU1-11P | RPL17P6 | RPL23AP42 | CCT7P2 | CNTNAP5-DT |
| RPS2P40 | ELOBP2 | ENSG00000263020 | GUCA1ANB | BTF3L4P2 | ENSG00000249141 | ENSG00000254536 | ENSG00000257524 | ENSG00000261832 | H2BW4P | HFE-AS1 | HNRNPA1P21 | HSPD1P11 | KRT8P3 | KRT8P39 | LINC02078 | LOC100192426 | LOC286359 | RPL7AP47 | RPS15P4 |
| MIR4717 | SMG1P2 | ULK4P2 | ANKRD26P3 | MIR7704 | MIR7847 | PTBP1P | PTMAP2 | RAD51L3-RFFL | RNU1-27P | RNVU1-1 | RNVU1-17 | RPL21P126 | RPL22P3 | RPL26P30 | RPL26P32 | RPL32P18 | RPL36AP8 | MIR3648-1 | MIR4433A |
| RPS25P6 | MIR6737 | MIR6833 | MIR7702 | SPINT5P | SUB1P1 | SVIL2P | TFDP1P2 | TMEM217B | TUBAP2 | WDR82P1 | ARPP19P1 | ATP5F1CP1 | BMS1P21 | CALM2P1 | CHCHD2P8 | CNPY3-GNMT | DBF4P1 | RPLP1P6 | RPS20P14 |
| EIF3FP3 | RPS2P46 | RPS2P5 | SNORD3P1 | ENSG00000267618 | ENSG00000285708 | ENSG00000285723 | ERVK-9 | FKSG29 | FSCN1P1 | GAPDHP60 | HNRNPA1P8 | IFITM8P | IGKV2-26 | IMMTP1 | KRT8P34 | LOC100129617 | LOC100240728 | E2F6P4 | EEF1A1P3 |
| MIR5787 | ENSG00000173867 | ENSG00000206549 | ENSG00000260537 | MIR6800 | MIR6819 | MIR6832 | MIR6852 | MIR6870 | MIR6875 | MIR6889 | MIR7703 | MIR7975 | OR6K4P | PABPC1P1 | PCED1CP | PHF2P2 | PPP1R14BP3 | LOC100289511 | LOC102724560 |
| RAB42P1 | MIR6069 | MIR6126 | MIR6775 | RNA5SP103 | RNA5SP74 | RNU1-28P | RNU5E-6P | RNVU1-14 | RNVU1-19 | RPL12P33 | RPL13AP2 | RPL18AP4 | RPL21P7 | RPL23AP74 | RPL27AP6 | RPL31P57 | RPL36AP7 | PTGES3P3 | PTMAP4 |
| RPL6P27 | RN7SL4P | RN7SL5P | RN7SL7P | RPS3P7 | RPS4XP17 | RPSAP26 | SETDB2-PHF11 | SMIM11P1 | VN1R106P | AKR1B1P3 | ARPP19P2 | BRDTP1 | CROCCP4 | CYP4F23P | DRD5P2 | EEF1B2P3 | ENSG00000124593 | RPL39P3 | RPL4P5 |
| ENSG00000255330 | RPL7AP66 | RPL7P9 | RPS18P5 | ENSG00000283189 | ENSG00000284762 | ENSG00000284969 | ENSG00000285446 | FDPSP1 | HMGA1P3 | HPRT1P2 | HSPD1P16 | LARP4P | LOC100132356 | LOC339902 | LYPLA2P2 | MIR3648-2 | MIR6727 | ENSG00000243501 | ENSG00000249773 |
| PTPN2P1 | ENSG00000272162 | ENSG00000272442 | ENSG00000282218 | RNU1-18P | RNU1-19P | RPL18P11 | RPL23AP14 | RPL27AP2 | RPL31P45 | RPL32P19 | RPL32P36 | RPL35AP35 | RPL35P5 | RPL36P11 | RPS15AP34 | RPS15P5 | RPS26P15 | MYL6P5 | PTMAP3 |
| RPS3P6 | RNA5SP117 | RNA5SP145 | RNA5SP259 | TOMM22P3 | UBTFL7 | UQCRFS1P2 | WBP2P1 | CDKN2AIPNLP1 | DDX3P3 | EEF1B2P6 | ENSG00000214265 | ENSG00000256646 | ENSG00000257065 | ENSG00000269741 | ENSG00000281613 | ENSG00000282804 | ENSG00000285791 | RPS26P28 | RPS27P3 |
| GET1P1 | RPS5P5 | SERF2-C15ORF63 | SETP4 | HMGN1P37 | IGHV4-80 | KRT8P48 | LNCRNA-ATB | LOC101928697 | LOC102724159 | LSM12P1 | MIR6826 | NDUFAF2P1 | RNA5SP202 | RNA5SP359 | RNA5SP452 | RPL31P50 | RPL32P28 | ENSG00000285991 | FTH1P23 |
| RPSAP14 | GNL3LP1 | HIGD1AP1 | HMGN1P31 | EIF2S2P7 | ENSG00000280148 | ENSG00000283977 | ENSG00000284554 | ENSG00000285733 | ENSG00000285976 | ENSG00000288564 | ENSG00000288640 | ENSG00000288712 | ENSG00000288721 | ENSG00000289694 | ENSG00000289722 | GCRG224 | H2ACP1 | RPS15AP9 | RPS2P14 |
| KRT18P11 | SCDP1 | ACTG1P14 | CYP4F9P | LOC642696 | LOC728024 | LSP1P5 | MRPS35P1 | MSRB1P1 | NAIPP1 | OR10Q2P | RNA5SP115 | RNA5SP191 | RNA5SP352 | RNA5SP53 | RNY5P1 | RPL18AP16 | RPL28P1 | H3P44 | HMGB1P40 |
| TRA-TGC10-1 | LOC101927960 | LOC392196 | LOC642502 | ENSG00000276302 | ENSG00000284341 | ENSG00000285085 | ENSG00000288529 | ENSG00000288646 | ENSG00000288708 | ENSG00000289701 | LOC101928354 | LOC105369388 | LOC440084 | LOC642943 | LOC649352 | MIR8069-1 | PNPP1 | RPL36AP10 | TMEM161BP1 |
| RPL15P15 | TRAPPC13P1 | ABCD1P5 | AZF1 | WBSCR23 | AAA1 | ACF | C12orf29P3 | ENSG00000288614 | LOC100420746 | LOC100421094 | LOC105378085 | LOC392187 | LOC442293 | LOC643576 | LOC728688 | RNA28SN5 | XS | RNA5-8SP3 | RNA5SP506 |
| LOC100129274 | RPLP1P7 | USP17L14P | USP17L16P | LOC642969 | LOC644667 | OCTN3 | PRO2268 | AIR | HPT | LOC106728418 | LOC107126281 | LOC107197952 | LOC108511947 | LOC108863620 | LOC109611593 | LOC111099027 | LOC111162621 | CORD1 | CYAT1 |
| LOC642204 | LOC108167315 | LOC110631417 | LOC110806263 | RNA5SP162 | THM | IGSF6-DREV1 | LOC106128905 | LOC107105350 | LOC107963948 | LOC110013312 | LOC110386947 | LOC110599571 | LOC111089946 | LOC111099028 | LOC111258525 | LOC111413032 | LOC117204001 | LOC390846 | LOC402329 |
| LOC647481 | OTSC4 | RIEG2 | RCHTS |  |  |  |  |  |  |  |  |  |  |  |  |  |  |  |  |
